# Zfp296 negatively regulates H3K9 methylation in embryonic development as a component of heterochromatin

**DOI:** 10.1038/s41598-017-12772-y

**Published:** 2017-09-29

**Authors:** Takumi Matsuura, Satsuki Miyazaki, Tatsushi Miyazaki, Fumi Tashiro, Jun-ichi Miyazaki

**Affiliations:** Division of Stem Cell Regulation Research, Osaka University Graduate School of Medicine, 2-2 Yamadaoka, Suita, 565-0871 Osaka Japan

## Abstract

The Cys2/His2-type zinc finger protein Zfp296 has been implicated in stem cell pluripotency and tumor pathogenesis. However, its mechanisms remain elusive. Here, we demonstrated that a Zfp296 deficiency in mice impairs germ-cell development and embryonic growth. Zfp296 was intracellularly localized to heterochromatin in embryos. A GST-Zfp296 pull-down experiment using ES cell nuclear extract followed by LC-MS/MS showed that Zfp296 interacts with component proteins of heterochromatin (such as HP1, Dnmt1, Dnmt3b, and ATRX) and the NuRD complex. We focused on H3K9 methylation as a hallmark of heterochromatin, and found that *Zfp296* overexpression in cultured cells reduces the Suv39h1-mediated H3K9 methylation. Consistent with this finding, in *Zfp296* ^−/−^ mouse embryos, we observed a global increase in H3K9 methylation in a developmental stage-dependent manner, and showed, by ChIP-qPCR, that the H3K9me3 levels at major satellite repeats were elevated in *Zfp296* ^−/−^ embryos. Our results demonstrate that Zfp296 is a component of heterochromatin that affects embryonic development by negatively regulating H3K9 methylation.

## Introduction

The pericentromeric region of a chromosome is composed of tandem arrays referred to as satellite DNA repeats, compacted into heterochromatin, which has been traditionally viewed as a static structure that is gene-poor and transcriptionally silent. However, recent genome-wide profiles of chromatin modifications and proteomics suggest that the formation and regulation of mammalian heterochromatin is probably more plastic than originally thought. Histone lysine methylation, such as H3 lysine 9 di- and trimethylation (H3K9me2 and -me3), and H4 lysine 20 di- and trimethylation (H4K20me2 and -me3), is a hallmark of heterochromatin^1^. In particular, H3K9me3 is essential for the induction of H4K20me2 and me3 by suppressor of variegation (Suv) 4-20 homologs 1 and 2 (Suv420h1 and h2)^2,3^. Suv 3-9 homologs 1 and 2 (Suv39h1 and h2) induce H3K9me2 and me3 at the heterochromatic region, and H3K9me3 is recognized and bound by heterochromatin protein 1 (HP1) family members^4,5^. HP1 isoforms, HP1α and γ, preferentially interact with Suv39h1 and increase Suv39h1 protein stability^6^. In addition, HP1 interacts with various chromosomal factors and is regarded as a central component of heterochromatin with roles in its structure and function. This binding network is crucial for heterochromatin formation and maintenance^7–10^. H3K9 methylation at mammalian heterochromatin is thought to be regulated by distinct protein complexes during development and in disease states; however, little is known about these mechanisms.

*Zfp296* encodes a conserved mammalian factor belonging to the Cys2/His2-type zinc finger (C2H2-ZF) family. C2H2-ZF proteins are typically considered sequence-specific DNA-binding transcription factors. On the other hand, C2H2-ZF proteins also function as chromatin effectors. For instance, ZNF644 and ZNF803/WIZ physically bind G9a/GLP histone methyltransferase complexes and co-regulate H3K9 methylation^11–13^. KRAB zinc finger proteins recruit KAP1 and repress transposable elements through histone modifications^14^. *Zfp296* was originally discovered as an oncogene candidate from a leukemic mouse model^15–17^ and was also identified as the translocation breakpoint in t(17;19)(q23;q13.32) pediatric acute myeloid leukemia (AML)^18^. Zfp296 is overexpressed in AML and acute lymphoid leukemia (ALL), and is associated with decreased survival in Philadelphia chromosome (Ph)-negative ALL^18^. In contrast, *Zfp296* transcription is silenced by 5′ CpG island hypermethylation in oligodendroglioma^19^ and prostate carcinoma^20^. Thus, in some cases, an aberrant expression of *Zfp296* appears to be involved in tumorigenesis or tumor progression. *Zfp296* is also highly expressed in human and mouse embryonic stem (ES) cells and in induced pluripotent stem (iPS) cells, but its expression progressively decreases during differentiation^21–23^. In addition, the expression of *Zfp296* in combination with Yamanaka factors *Oct3/4*, *Sox2*, *Klf4*, and *c-Myc*, enhances the efficiency of iPS cell generation^24^. These observations suggest that Zfp296 regulates pluripotency in ES cells and iPS cells. Zfp296 has been proposed to function as a transcription factor in pluripotent cells^24^, but its roles in tumorigenesis and other biological processes remain undefined.

In the present study, we generated *Zfp296* knockout mice and found that Zfp296 is required for proper germ-cell development and embryonic growth. We also found that Zfp296 is localized to the DAPI (4′,6-diamidino-2-phenylindole)-dense heterochromatin foci in embryonic somatic cells. In addition, we showed that Zfp296 binds to components of heterochromatin and the nucleosome remodeling and deacetylase (NuRD) complex *in vitro* and that Zfp296 overexpression decreases Suv39h1-mediated H3K9 methylation in HEK293T cells. We observed that the H3K9 methylation levels in *Zfp296* ^−/−^ embryonic cells increase in a developmental stage-dependent manner and that Zfp296 deficiency affects the H3K9me3 levels at major satellite repeats. Based on these results, we discuss the roles of Zfp296 as a temporal repressor of H3K9 methylation during mammalian embryogenesis and as a chromatin effector at the H3K9me-dependent heterochromatin in embryonic somatic cells.

## Results

### *Zfp296*^−/−^ mice are healthy but partially infertile, with small testes and ovaries

**Figure 1:**
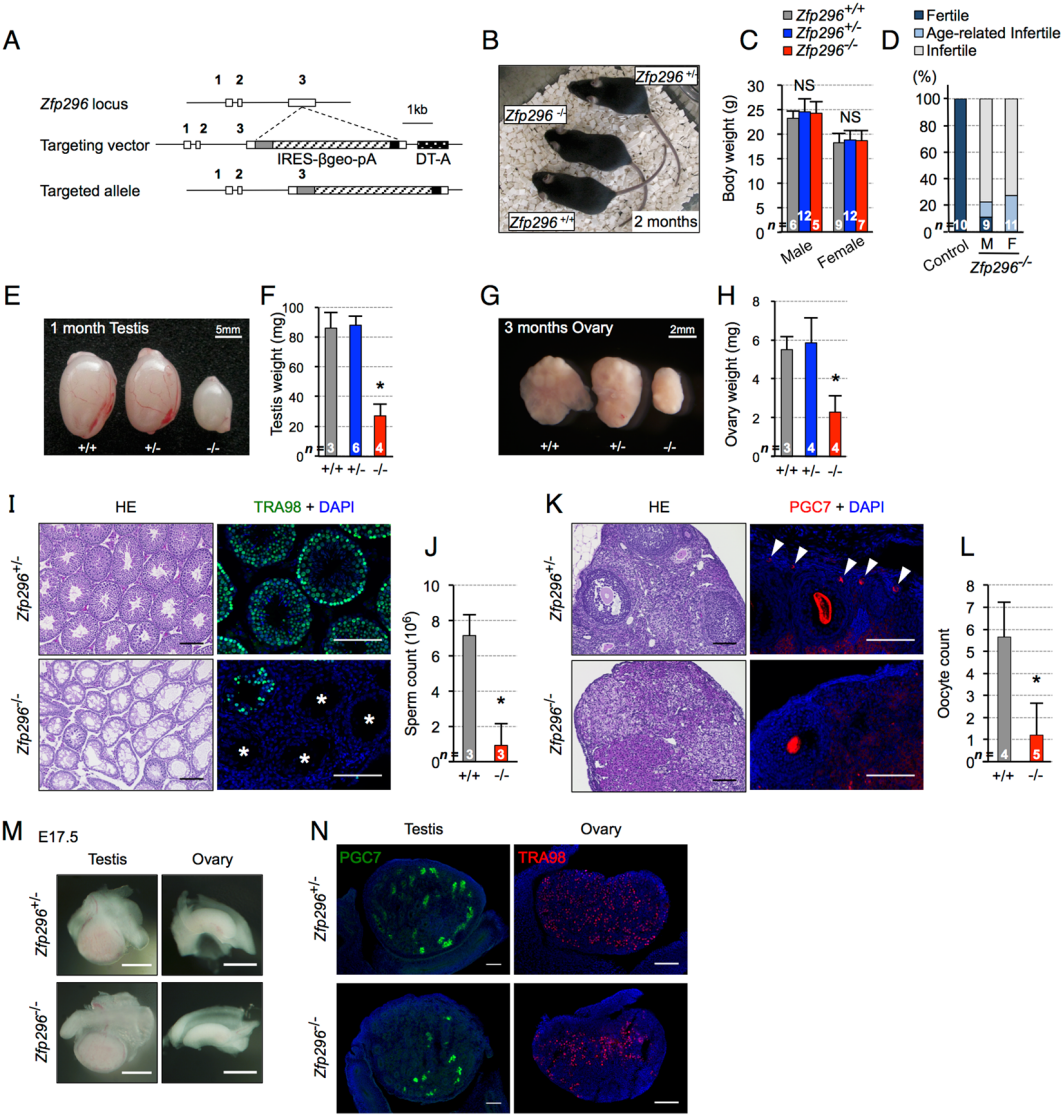
Generation of *Zfp296* ^−/−^ mice and their apparent phenotypes. (**A**) Schematic diagram of the *Zfp296* gene locus, the targeting vector, and the targeted allele. IRES: internal ribosome entry site. β-geo: β-garactosidase + neomycin phosphotransferase fusion gene. pA: polyadenylation signal. DT-A: diphtheria toxin A-fragment gene. (**B**) Gross appearance of adult *Zfp296*-deficient mice at 2 months of age. (**C**) Body weight of control and *Zfp296* ^−/−^ mice. Age ranged from 2 to 3 months. (**D**) Frequency analysis of infertility between Zfp296^−/−^ and control (Zfp296^+/+^ or Zfp296^+/−^) mice. Age ranged from 3 to 7 months. Age-related infertility is defined as an inability to become pregnant at 5 months of age. (**E** and **F**) Appearance and weight of the adult testes from littermate mice at 1 month of age. (**G** and **H**) Appearance and weight of the adult ovaries from littermate mice at 3 months of age. (**I**) Hematoxylin and eosin-stained sections of testes from the indicated genotypes (1 month-old) (left). Representative testis sections immunofluorescently stained for the germline marker TRA98 (right). DNA was counterstained with DAPI. Asterisks indicate seminiferous tubules showing germ cell loss. Scale bar = 100 μm. (**J**) Number of mature sperm contained in the epididymides of control and *Zfp296* ^−/−^ mice at 2 months of age. (**K**) Sections of adult ovaries (3 month-old) stained with hematoxylin and eosin (left). Representative ovary sections immunofluorescently stained for the germ-cell marker PGC7 (right). DNA was counterstained with DAPI. Arrowheads indicate primordial follicles. Scale bar = 100 μm. (**L**) Oocyte counts in fully grown control and *Zfp296* ^−/−^ mice at 3 months of age. Number of OOEP-positive oocytes per section was counted using immunofluorescence. (**M**) Gross images of the E17.5 embryonic testis and ovary from littermate mice. Scale bar = 500 μm. (**N**) Immunofluorescence staining of E17.5 *Zfp296* ^+/−^ and *Zfp296* ^−/−^ embryonic testis and ovary sections for germ-cell markers: PGC7 (left) and TRA98 (right). Data represent mean + SD. NS, not significant; *p < 0.01 by two-tailed Student’s *t*-test.

We generated *Zfp296* ^−/−^ mice from *Zfp296* ^+/−^ intercrosses, and confirmed the loss of Zfp296 protein in the testis by Western blotting (Fig. S1D; see Figs 1A and S1A–S1C). *Zfp296* ^−/−^ mice were viable and healthy with a normal body weight (Fig. 1B and C), and showed no signs of abnormal hematopoiesis or neurogenesis (data not shown). We also detected no changes in the frequency of spontaneous tumorigenesis (data not shown) or in lifespan (Fig. S1E). Bcl11a/Ctip1 and Bcl11b/Ctip2, putative paralogs of Zfp296 (see Fig. S1F for homology), are required for lymphoid development^25,26^. However, flow cytometry analysis of the thymocytes and peripheral blood cells from *Zfp296* ^−/−^ mice revealed no abnormalities in the expression of lymphocyte surface markers (Fig. S1F and S1G). This result indicated that Zfp296 may not be involved in lymphocyte development, and that its molecular role is probably distinct from that of its putative paralogs Bcl11a/Ctip1 and Bcl11b/Ctip2.

Previous studies suggested that Zfp296 is mainly expressed in germ-cell lineages, which include post-meiotic spermatids^15^ and germ-line stem cells^24^. We therefore investigated the effects of Zfp296 ablation on fertility. When *Zfp296* ^−/−^ mice were intercrossed (9 mating pairs), no progeny were obtained. In addition, mating experiments between *Zfp296* ^−/−^ and wild-type mice revealed that 70–80% of the male and female *Zfp296* ^−/−^ mice were infertile, and that fertile *Zfp296* ^−/−^ mice tended to lose their fertility at a younger age than control mice (Fig. 1D). The testis and ovary of *Zfp296* ^−/−^ mice were significantly smaller than those of *Zfp296* ^+/+^ and *Zfp296* ^+/−^ mice (Fig. 1E–H). In contrast, fertility defects were not detected in *Zfp296* ^+/−^ mice; we therefore used not only wild-type mice but also *Zfp296* ^+/−^ mice as controls. Histological and immunofluorescence analyses showed that the testis (2 months postnatal) of *Zfp296* ^−/−^ mice was mostly occupied by degenerated tubules lacking germ-line cells (Fig. 1I; see Fig. S1H for postnatal development of the testis). Such degeneration in the tubules is considered to represent an impairment in spermatogenesis, and in fact, the production of mature sperm was greatly reduced (Fig. 1J). Similarly, we observed that oocytes at all stages of development were decreased in the ovary (3 months postnatal) of *Zfp296* ^−/−^ mice (Fig. 1K and L; see Fig. S1I for postnatal development of the ovary). These results indicated that a severe reduction in the number of sperm and oocytes led to impaired fertility in *Zfp296* ^−/−^ mice. In contrast to adult mice, the embryonic gonads at E17.5 appeared to be the same size in *Zfp296* ^−/−^ and control mice (Fig. 1M). However, immunofluorescence analysis for the germ-cell markers PGC7 and TRA98 showed decreased numbers of germ cells in the E17.5 embryonic gonads of *Zfp296* ^−/−^ mice compared with control mice (Fig. 1N). Collectively, these findings demonstrated that a deficiency in Zfp296 caused a reduction in germ-cell numbers in the embryonic gonad, resulting in a degenerated testis and ovary during postnatal development.

### Loss of Zfp296 causes impaired primordial germ-cell development

**Figure 2:**
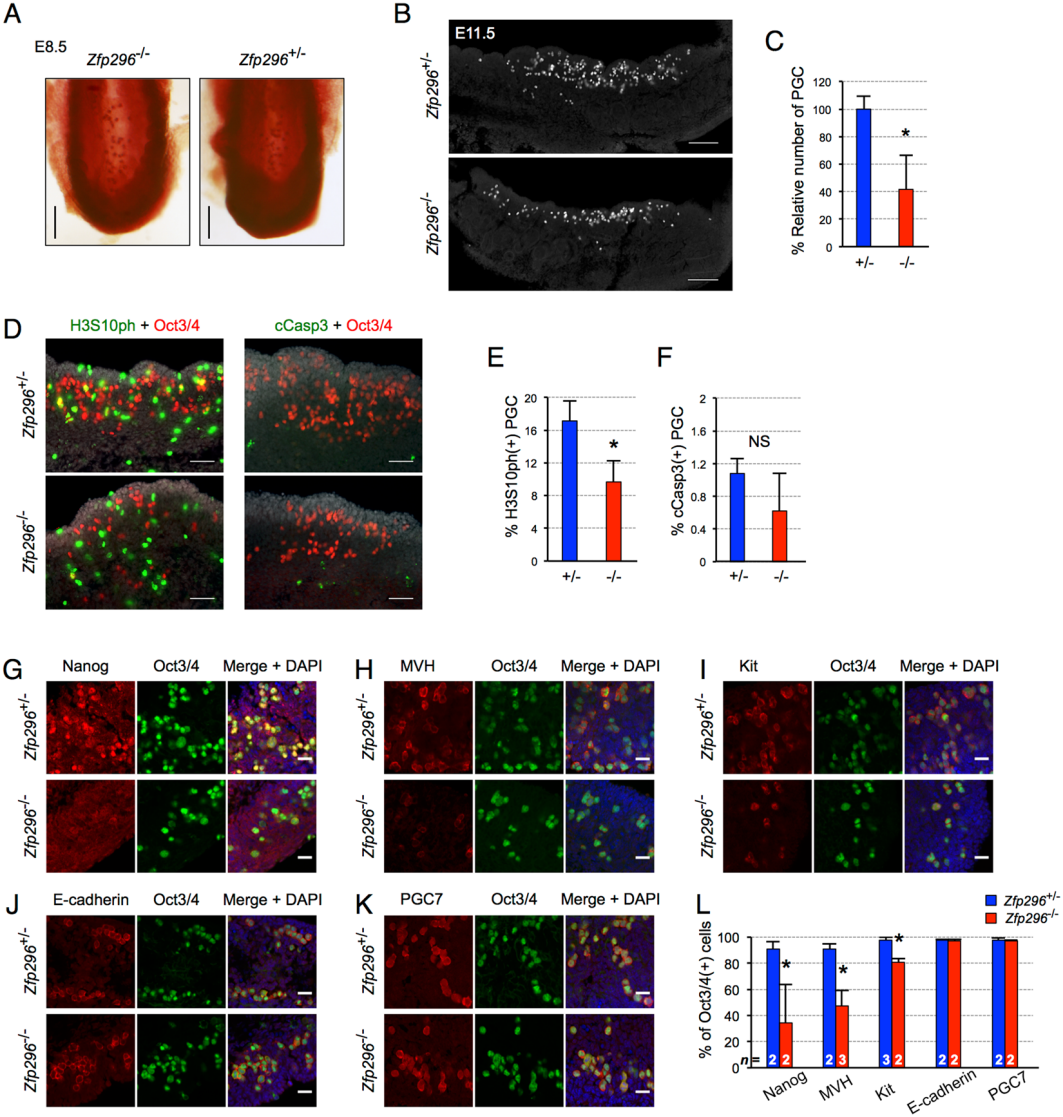
Impaired germ-cell development in *Zfp296* ^−/−^ mice. (**A**) Alkaline phosphatase staining of PGCs in the posterior part of E8.5 *Zfp296* ^+/−^ and *Zfp296* ^−/−^ embryos. (**B**) Immunofluorescence staining of E11.5 *Zfp296* ^+/−^ and *Zfp296* ^−/−^ genital ridge sections for Oct3/4. Oct3/4 served as a marker for PGCs. Scale bar = 100 μm. (**C**) Number of PGCs in E11.5 genital ridges of *Zfp296* ^−/−^ embryos relative to that in *Zfp296* ^+/−^ embryos. PGCs were counted in serial sections of genital ridges from four embryos for each genotype. Data represent mean + SD. **p < 0.01 by two-tailed Student’s *t*-test. (**D**) PGC proliferation and apoptosis in *Zfp296* ^−/−^ embryos. Representative immunofluorescence images for Oct3/4 (red) and phospho-histone H3 (H3S10ph, green, left) or cleaved caspase-3 (cCasp3, green, right) in E11.5 genital ridge sections. DNA was counterstained with DAPI (gray). Scale bar = 50 μm. (**E**) Percentage of Oct3/4-positive cells that were H3S10ph-positive in serial sections of genital ridges stained as shown in (**D**). Data represent mean + SD from four embryos for each genotype. *p < 0.05 by two-tailed Student’s *t*-test. (**F**) Percentage of Oct3/4-positive cells that were cCasp3-positive in serial sections of genital ridges stained as shown in (**D**). Data represent mean + SD from three embryos for each genotype. (**G**–**K**) Immunofluorescence staining of E12.5 *Zfp296* ^+/−^ and *Zfp296* ^−/−^ genital ridge sections for the indicated markers of PGC differentiation. DNA was counterstained with DAPI. Scale bar = 20 μm. (**L**) Percentage of Oct3/4-positive cells in (**G**–**K**) that were double-positive. Data represent mean + SD from the indicated number of embryos for each genotype. *p < 0.05, **p < 0.01 by two-tailed Student’s *t*-test.

We next assessed the effects of Zfp296 ablation on earlier germ-cell development. In the mouse, primordial germ cells (PGCs) are specified by E7.25, and then migrate into the developing hindgut at E8.5^27^. From E7.25, PGCs stain positively for alkaline phosphatase (AP)^27^. AP staining of E8.5 hindguts revealed no difference in the number of PGCs between *Zfp296* ^−/−^ and control mice (Fig. 2A). In addition, AP staining of *Zfp296* ^−/−^ versus control embryos at around E9.5 revealed no difference in PGC migration (Fig. S2A and S2B). These findings indicated that Zfp296 does not affect the specification and migration of PGCs. We next examined whether Zfp296 influences the post-migratory PGC biology. Immunostaining of E11.5 genital ridge sections for Oct3/4 showed that the number of PGCs was markedly decreased in *Zfp296* ^−/−^ embryos compared with controls (Fig. 2B and C). Analysis of the mitotic marker phospho-histone H3 (H3S10ph) showed that the proliferation of PGCs was significantly decreased in the E11.5 *Zfp296* ^−/−^ genital ridge (Fig. 2D and E). However, we observed no difference in the apoptosis of PGCs between *Zfp296* ^−/−^ and control embryos at E11.5 as assessed by cleaved caspase-3 staining (Fig. 2D and F).

We next assessed the differentiation status of the PGCs in E12.5 *Zfp296* ^−/−^ embryos. Nanog^28^, MVH^29^, and Kit^30^ begin to be expressed in PGCs soon after their specification and are highly expressed in almost all of the PGCs at E12.5. Notably, immunofluorescence analyses of E12.5 *Zfp296* ^−/−^ genital ridges revealed Oct3/4-positive cells that were negative for Nanog, MVH, or Kit (Fig. 2G–I), and the percentages of Oct3/4-positive cells that were positive for these proteins were significantly lower in the *Zfp296* ^−/−^ genital ridges than that in the controls (Fig. 2L). Considering the previous report that Zfp296 acts as a transcriptional activator of *Oct3/4* through its germ cell specific conserved region 4 and that overexpression of *Zfp296* in ES cells leads to *Nanog* upregulation^24^, *Oct3/4* and *Nanog* might be downregulated in the PGCs of *Zfp296* ^−/−^ mice. However, we detected no considerable changes in Oct3/4 expression in comparison with the other PGC markers such as E-cadherin and PGC7 by immunofluorescence (Fig. 2﻿J-L). On the other hand, Nanog was clearly downregulated in the PGCs of E12.5 *Zfp296* ^−/−^ embryos (Fig. 2L). However, immunostaining analyses of E13.5 embryonic gonads showed a partial recovery of PGC marker expression in *Zfp296* ^−/−^ mice (Fig. S2C), indicating that the repression of Nanog, MVH, and Kit expression observed in the PGCs of E12.5 *Zfp296* ^−/−^ embryos was developmental stage-dependent. Collectively, these results suggested that Zfp296 more strongly affects the differentiation status of PGCs in the post-specification phase than in the specification and migration phase.

### *Zfp296*^−/−^ mice exhibit partial embryonic lethality and growth retardation

**Figure 3:**
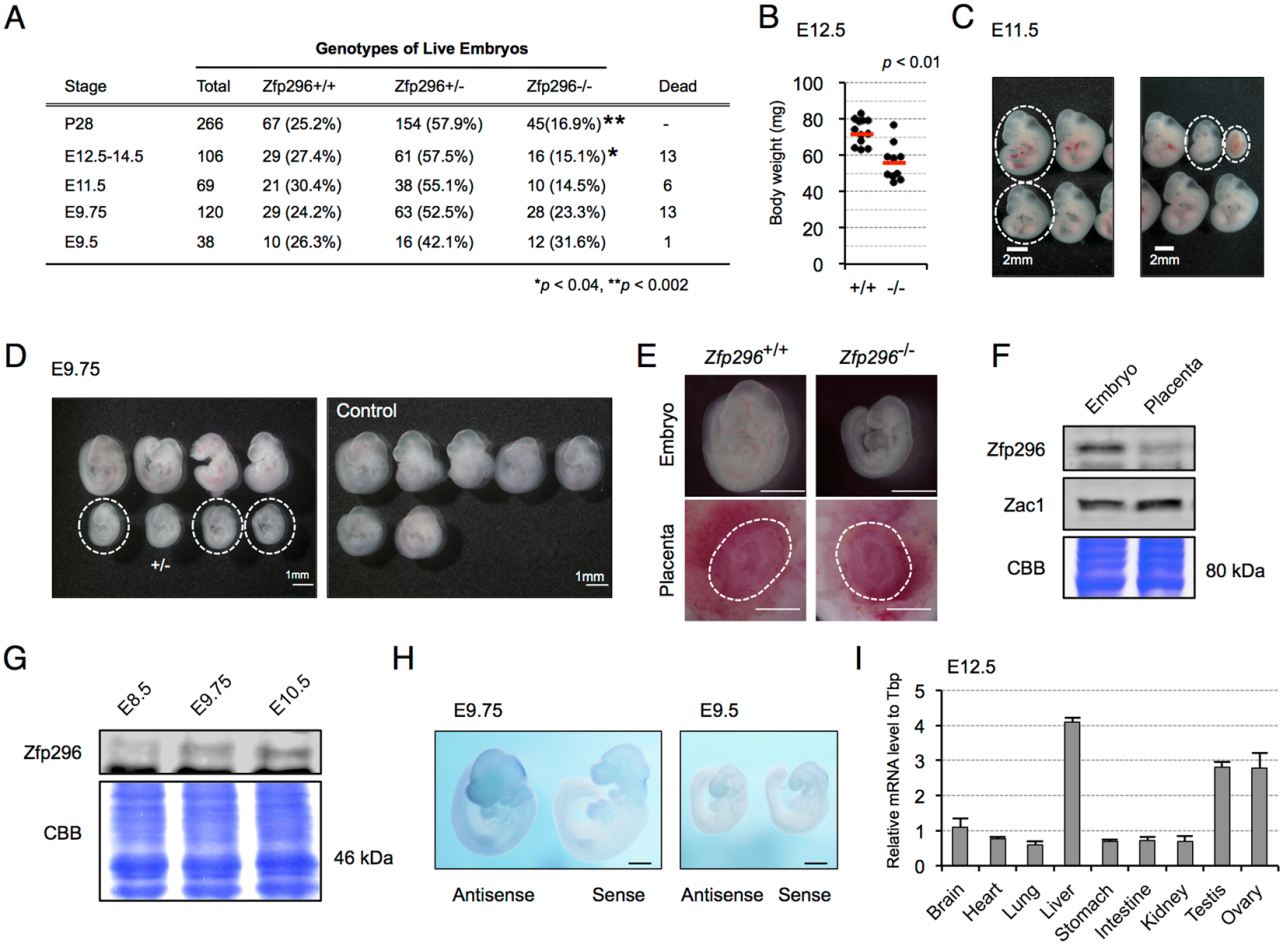
*Zfp296* ^−/−^ mice exhibit partial embryonic lethality and growth retardation. (**A**) Genotypes of offspring from *Zfp296* ^+/−^ intercrosses. Segregation ratio was analyzed with the chi-square test against the expected Mendelian ratio of 1:2:1. *p < 0.04, **p < 0.002. (**B**) Body weights of E12.5 wild-type and *Zfp296* ^−/−^ embryos. Red line indicates the average value. *p < 0.01 by two-tailed Student’s *t*-test. (**C**) E11.5 littermate embryos from *Zfp296* ^+/−^ intercrosses. Dashed circles indicate *Zfp296* ^−/−^ embryos. (**D**) E9.75 embryos from a single litter of wild-type crosses (control) and *Zfp296* ^+/−^ intercrosses. Dashed circles indicate *Zfp296* ^−/−^ embryos. (**E**) Representative whole-mount images of an E9.75 embryo and placenta of the indicated genotypes. Dashed circle indicates the placental region. Scale bar = 1 mm. (**F**) Western blot analysis of the lysates from an E9.75 wild-type whole embryo and placenta. Zac1 served as a marker protein of placenta. Coomassie brilliant blue (CBB) staining shows that equal protein amounts were loaded. (**G**) Western blot analysis for Zfp296 in the lysates from E8.5-E10.5 wild-type whole embryos. CBB staining was used as a loading control. (**H**) Whole-mount RNA *in situ* hybridization to detect Zfp296 mRNA in E9.5-E9.75 wild-type embryos. Scale bar = 500 μm. (**I**) Analysis of Zfp296 mRNA expression in various tissues of E12.5 wild-type embryos by qRT-PCR; mRNA levels were normalized to that of Tbp. Data represent mean + SD of three experiments with two samples.

While analyzing the role of Zfp296 in germ-cell development, we noticed that the *Zfp296* deficiency sometimes caused embryonic death at around E9.5-E14.5, and that from E12.5 on, the proportion of *Zfp296* ^−/−^ mice was significantly lower than that expected from Mendel’s rule (Fig. 3A). We also found a growth retardation of *Zfp296* ^−/−^ embryos during E9.75-E12.5, which was not apparent before E9.5 or after E14.5 (Fig. S3A and S3B). Although at E12.5 some of the *Zfp296* ^−/−^ embryos were similar in size to controls, the average body weight of the *Zfp296* ^−/−^ embryos was significantly lower than that of controls (Fig. 3B). At E11.5, the *Zfp296* ^−/−^ embryos exhibited various degrees of growth retardation (Fig. 3C). At E9.75, almost all of the *Zfp296* ^−/−^ and a few *Zfp296* ^+/−^ embryos exhibited slowed growth (Fig. 3D). We confirmed that placental defects were not seen with any of the growth-retarded *Zfp296* ^−/−^ embryos from intercrosses of *Zfp296* ^+/−^ mice (Fig. 3E). Western blot analysis revealed that, in wild-type at E9.75, the Zfp296 expression was much higher in the embryos than in the placenta (Fig. 3F), consistent with the finding that Zfp296 ablation did not affect placental development. We next analyzed the change in Zfp296 protein levels in wild-type embryos from E8.5-E10.5 by Western blotting, and found that the Zfp296 protein level began to increase from E9.75 (Fig. 3G). Consistent with this observation, whole-mount *in situ* hybridization analysis revealed that the *Zfp296* mRNA expression was ubiquitously increased in E9.75 embryos (Fig. 3H). These findings suggested that upregulation of *Zfp296* from E9.75 might be related to the growth defects seen in the *Zfp296* ^−/−^ embryos. On the other hand, qRT-PCR analysis of various tissues at E12.5 demonstrated that *Zfp296* was preferentially expressed in the liver, testis, and ovary (Fig. 3I). Although *Zfp296* ^−/−^ mice showed no abnormalities in the liver during pre- and postnatal development, high-level expression of *Zfp296* in the testis and ovary at E12.5 might be correlated with the aberrant germ-cell development phenotypes observed in the *Zfp296* ^−/−^ mice.

### Zfp296 interacts with components of heterochromatin *in vitro* and affects H3K9 methylation in cultured cells

**Figure 4:**
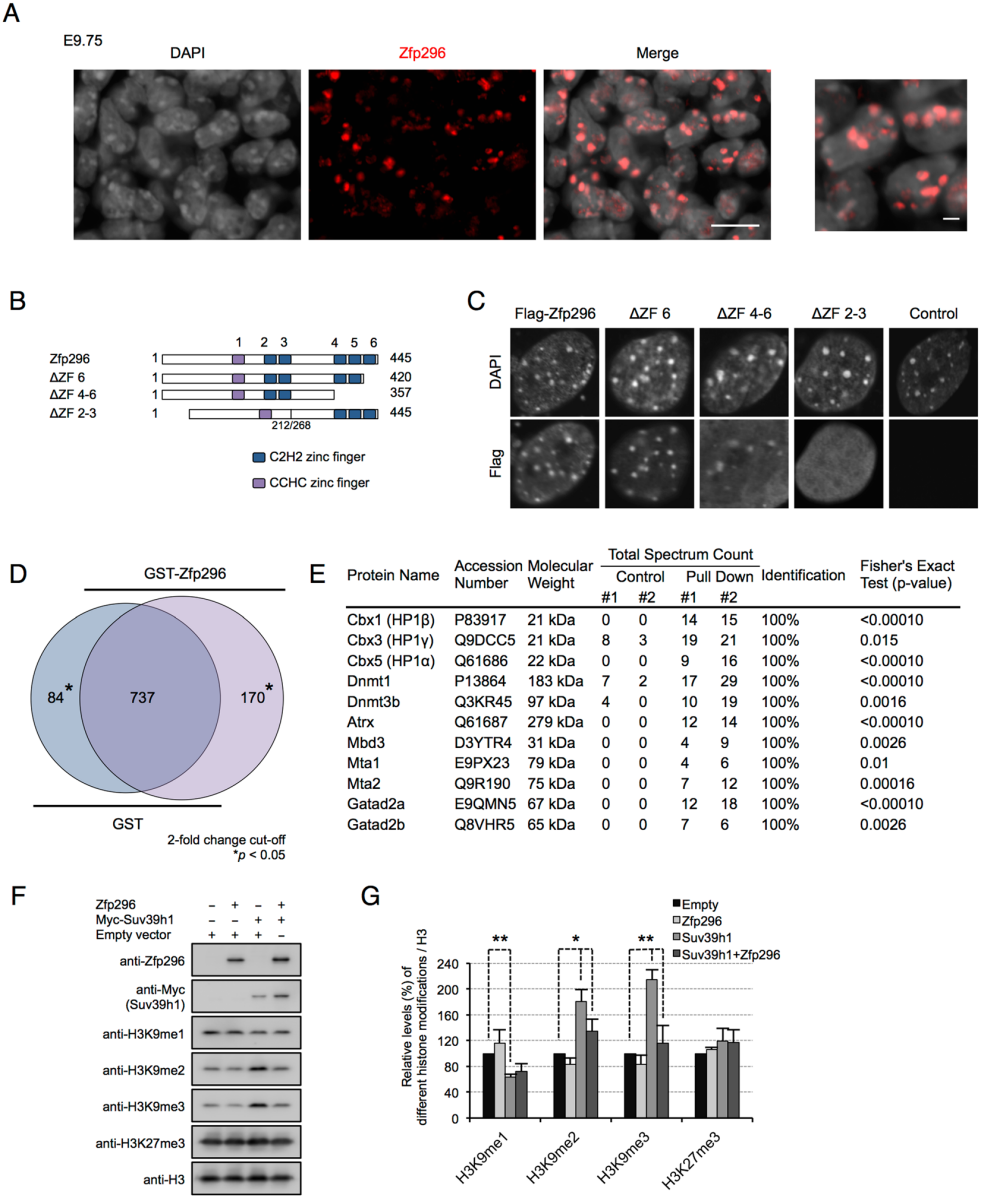
Zfp296 is a component of heterochromatin in mammalian cells. (**A**) Immunofluorescence staining of an E9.75 wild-type embryo for Zfp296. Nuclei were counterstained with DAPI. Scale bar = 10 μm. Right panel shows a magnified view. Scale bar = 2 μm. (**B**) Domain structure and deletion mutants of Zfp296 used in this study. Mouse Zfp296 consists of 445 amino acid residues and has one C2H2 zinc finger domain and five CCHC zinc finger domains. ΔZF 2–3 has an internal deletion of amino acid residues 213–267. (**C**) Immunofluorescence staining of primary MEFs transfected with a plasmid vector expressing Flag-tagged wild-type Zfp296 or its mutant constructs shown in (**B**). Nuclei were stained with an anti-Flag antibody followed by a fluorescein-labeled second antibody, counterstained with DAPI, and observed by fluorescence microscopy. (**D**) GST pull-down assays. Control GST protein or GST-Zfp296 fusion protein was incubated with ES cell nuclear extracts, and the pulled-down samples were subjected to LC-MS/MS shotgun proteomics. Venn diagram shows the number of proteins identified in two experiments (minimum value of total spectrum counts was normalized to 2, 2-fold change cut-off). *p < 0.05 by Fisher’s exact test. (**E**) Representative Zfp296-binding partners contained in heterochromatin and the NuRD complex. (**F**) Western blot analysis of total cell extracts from HEK293T cells expressing Zfp296 and/or Myc-Suv39h1. The indicated antibodies were used for detection. (**G**) Quantification of the data shown in (**F**). The density of each band relative to that obtained with anti-histone H3 was determined. The level of each histone modification was expressed relative to the level in HEK293T cells transfected with empty vector. Data represent mean + SD of three independent experiments. *p < 0.05, **p < 0.01 by two-tailed Student’s *t*-test.

To address the potential functions of Zfp296 in further depth, we analyzed the intracellular localization of Zfp296 by immunofluorescence analysis of E9.75 embryos using confocal laser scanning microscopy, and found that Zfp296 was localized to the DAPI-dense heterochromatin foci (Fig. 4A). Such Zfp296 staining was lost in the *Zfp296* ^−/−^ embryos (Fig. S4A). We next determined the domains responsible for its heterochromatin localization. We performed immunofluorescence studies in primary MEF cells (which were derived from E14.5 mouse embryos and showed very weak or no endogenous expression of Zfp296 protein) transfected with plasmid vectors expressing Flag-Zfp296 or its deletion mutants shown in Fig. 4B. Zfp296 contains six ZF domains composed of a single CCHC-ZF and five C2H2-ZF domains. The results revealed that the heterochromatin localization of Zfp296 was dependent on its second and third ZF domains (Fig. 4C). We also found that the fourth to sixth ZF domains of Zfp296 were required for its nucleus-specific localization (Fig. 4C).

Bcl11b/Ctip2, a putative paralog of Zfp296, physically recruits chromatin-modifying complexes and establishes the heterochromatic environment^31,32^. Thus, we hypothesized that Zfp296 also binds to heterochromatin components. To examine this possibility, we carried out GST-pull down experiments using ES cell nuclear extract and recombinant GST-Zfp296 or GST (as a control). The captured proteins were subsequently analyzed by liquid chromatography-tandem mass spectrometry (LC-MS/MS). We identified 170 proteins that showed significant binding to GST-Zfp296 compared with GST alone (2-fold change cut-off; p < 0.05, Fisher’s exact test) (Fig. 4D and Table S1). Among these Zfp296-interacting proteins, we found components of heterochromatin and the NuRD complex (Fig. 4E). These findings were consistent with a previous study indicating that ZNF296 (the human homolog of Zfp296) interacts with MBD3L1^33^. Interestingly, DNA repair proteins such as H2AX, MSH6, DDB1, LIG3, and MDC1 were also shown to significantly bind to GST-Zfp296 (Table S1). In addition, GST-Zfp296 appeared to pull down endogenous Zfp296 (Table S1). Another coimmunoprecipitation analysis in human embryonic kidney (HEK) 293 T cells revealed that Zfp296 might form oligomers (Fig. S4B and S4C). Similarly, Bcl11a/Ctip1 is reported to form oligomers *in vivo* ^34^.

H3K9 methylation is involved in heterochromatin formation and heterochromatic gene silencing. Suv39h is known to induce H3K9 trimethylation at pericentromeric heterochromatin^35^. Therefore, we next assessed the effect of *Zfp296* expression on Suv39h-dependent H3K9’s methylation. The transient transfection of HEK293T cells with a plasmid vector expressing Myc-Suv39h1 induced an increase in H3K9me2 and H3K9me3, which was inhibited by the coexpression of Zfp296 (Fig. 4F and G). We also observed by live-cell imaging that Zfp296-GFP colocalized with Suv39h1-DsRed at heterochromatin foci in HEK293T cells (Fig. S4D). Taken together, these findings indicated that Zfp296 targets heterochromatin and represses Suv39h-dependent H3K9 di- and trimethylation.

### *Zfp296*^−/−^ embryos exhibit whole-body H3K9 hypermethylation

**Figure 5:**
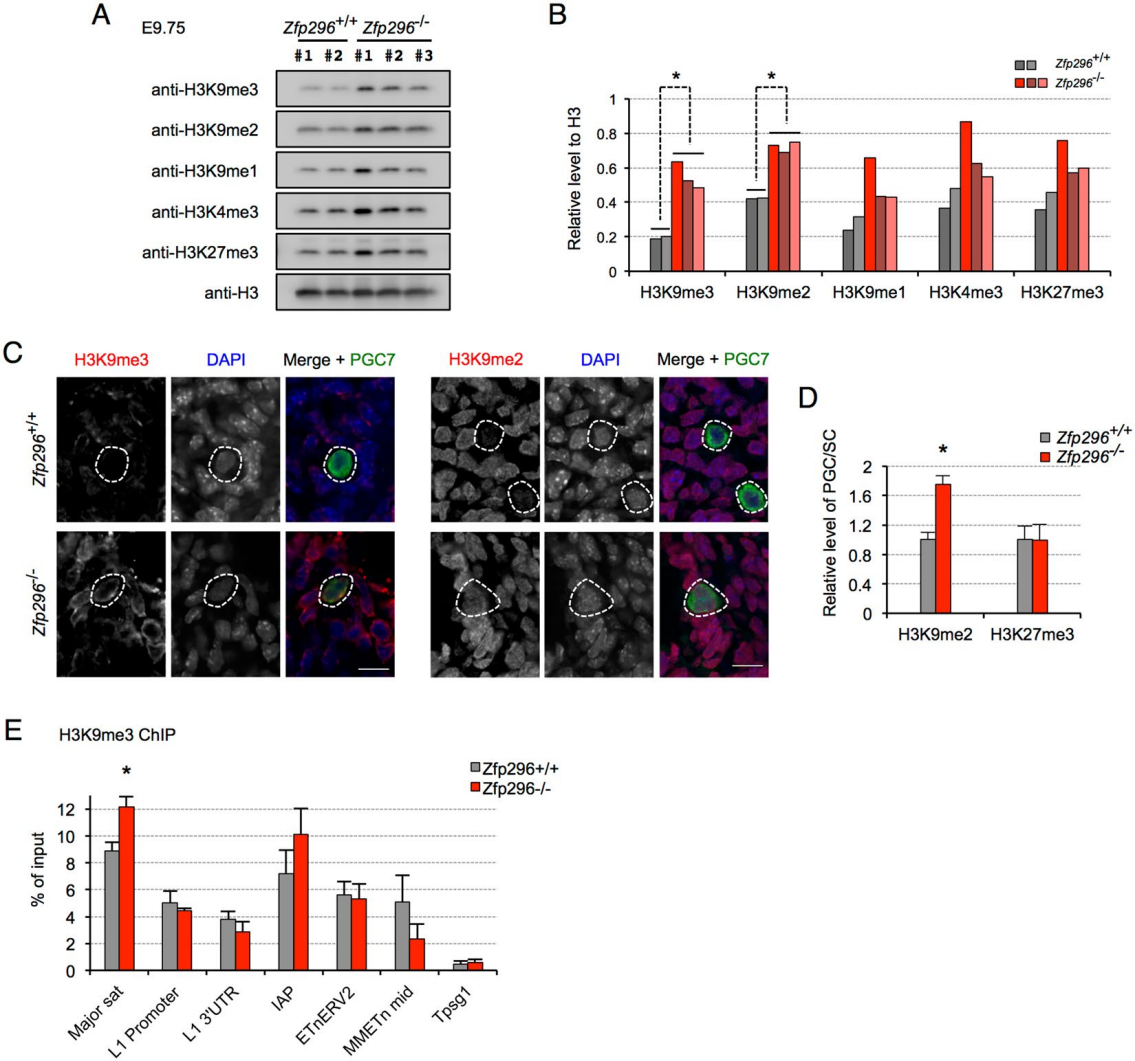
H3K9 hypermethylation of *Zfp296* ^−/−^ mice in embryonic development. (**A**) Western blot analysis of whole-cell lysates from E9.75 wild-type and *Zfp296* ^−/−^ embryos. Histone H3 served as a loading control. (**B**) Quantification of the data shown in (A). The level of each histone modification normalized to that of H3 is shown for individual embryos. *p < 0.01 by two-tailed Student’s *t*-test. (**C**) Immunofluorescence staining of genital ridge sections from E9.75 wild-type and *Zfp296* ^−/−^ embryos for H3K9me3 (left) or H3K9me2 (right). PGC7 served as a marker for PGCs. DNA was counterstained with DAPI. Scale bar = 10 μm. (**D**) Signal intensity of H3K9me2 and H3K27me3 in PGCs relative to somatic cells. Signal intensity in PGCs was measured with software and normalized to that of three neighboring somatic cells. Data represent mean + SD of 20-25 PGCs from two embryos for each genotype. *p < 0.01 by two-tailed Student’s *t*-test. (**E**) H3K9me3 ChIP-qPCR at the indicated loci in E9.75 wild-type and *Zfp296* ^−/−^ whole embryos. Chromatin was prepared from a pool of four embryos for each genotype and subjected to ChIP assay using anti-H3K9me3 antibody. The resulting DNA samples were assayed by qPCR in triplicate. Data represent mean + SD. *p < 0.05 by two-tailed Student’s *t*-test. The *Tpsg1* gene locus was chosen as a non-target control locus, as previously described^55^.

Having found that heterochromatin-localized Zfp296 could repress H3K9 methylation in cultured cells, we next examined whether a Zfp296 deficiency could affect H3K9me3 levels during embryogenesis. We first compared the H3K9 methylation levels in E9.75 *Zfp296* ^−/−^ versus control embryos by quantitative Western blotting, and notably, we detected a global increase in H3K9me2 and H3K9me3 (Fig. 5A and B). To confirm this result, we immunostained hindgut sections of E9.75 *Zfp296* ^−/−^ and control embryos for H3K9me1, H3K9me2, and H3K9me3. Consistent with the Western blotting results, we found that H3K9me3 was elevated in both the somatic cells and the PGCs, that H3K9me2 was elevated, particularly in the PGCs, and that no considerable changes were observed for H3K9me1, in the *Zfp296* ^−/−^ embryos compared to wild-type (Figs 5C and S5A). PGCs are known to exhibit decreased H3K9me2 and increased H3K27me3 during their migration phase^36^. By measuring the immunofluorescence intensity, we found that the Zfp296 deficiency caused an upregulation of H3K9me2 but did not affect the H3K27me3 level in PGCs (Figs 5D and S5B). This finding suggested that the Zfp296 loss could disturb the epigenetic regulation in PGC development. On the other hand, after E11.5, we found no difference in the H3K9 methylation levels between *Zfp296* ^−/−^ and the control embryos (data not shown). These observation appeared to be correlated with the appearance of the Zfp296-deficient phenotypes.

Zfp296 has been regarded as a transcription factor in previous studies^21–23^. To explore the cause of the increased H3K9 methylation observed in *Zfp296* ^−/−^ embryos, we analyzed the expression of several key genes involved in H3K9 methylation in E9.75 *Zfp296* ^−/−^ and control embryos, by qRT-PCR. We detected significant transcriptional differences in *Eset* and *Jmjd2c* between the *Zfp296* ^−/−^ and control embryos (Fig. S5C). However, these changes were relatively small (within 2-fold). These findings suggested that Zfp296 suppresses H3K9 methylation in the developing embryos by a mechanism other than the direct transcriptional regulation of H3K9-modifying enzymes.

The pericentromeric region is enriched with large clusters of repetitive satellite sequences and transposable elements, which are characterized by highly methylated H3K9. We next examined whether Zfp296 ablation affected the H3K9me3 levels at repetitive DNA elements. For this analysis, we performed a chromatin immunoprecipitation (ChIP) assay of E9.75 embryos with anti-H3K9me3, followed by qPCR. The H3K9me3 levels at major satellite repeats were significantly elevated in the *Zfp296* ^−/−^ embryos compared with controls (Fig. 5E). Taken together, these results indicate that Zfp296 temporally modulates H3K9 methylation levels at heterochromatin during embryogenesis.

## Discussion

Zfp296 has been described as a DNA-binding transcription factor^24^, but here we showed that it can directly bind to heterochromatin-related proteins. Similarly, Bcl11b/Ctip2, a putative paralog of Zfp296, interacts with HP1 and Suv39h1. A Bcl11b/Ctip2-containing complex was reported to promote H3K9 methylation^31,32^. In contrast, here we observed that Zfp296 decreased the H3K9 methylation levels in HEK293T cells. This finding was supported by the observation that the H3K9me3 levels in *Zfp296* ^−/−^ embryos were globally elevated. Together, these results suggest that Zfp296 and Bcl11b/Ctip2 may play opposing roles in the H3K9 methylation of heterochromatin.

H3K9 modification appears to have critical roles in embryogenesis; this importance is highlighted by the finding that mutations in H3K9-modifying enzymes cause severe embryonic growth defects^37^. Consistent with this point, we found that Zfp296 ablation in mice resulted in partial embryonic lethality and growth retardation at E9.5-E14.5. Recent genome-wide profiles of chromatin modifications in various embryonic cells and tissues have revealed that these modifications are temporally regulated during development^38^. In *Zfp296* ^−/−^ embryonic cells, elevated levels of H3K9 were seen at heterochromatin, but not after E13.5 (data not shown), and the defective phenotypes of *Zfp296* ^−/−^ embryos coincided with the temporal expression pattern and levels of Zfp296. This finding leads to the hypothesis that Zfp296 serves as a temporal repressor of H3K9 methylation during embryogenesis. Epigenetic reprogramming in PGCs involves a widespread loss of both H3K9 methylation^36,39,40^. We showed that the H3K9me2, H3K9me3 were elevated in the *Zfp296* ^−/−^ PGCs. In addition, we observed that the expressions of Nanog, MVH, and Kit were reduced in *Zfp296* ^−/−^ PGCs compared with those in control PGCs. The expression of these genes in PGCs is known to be upregulated through epigenetic reprogramming^39,41^. Therefore, it is likely that the inappropriate differentiation of PGCs seen in *Zfp296* ^−/−^ mice resulted from H3K9 hypermethylation and incomplete epigenetic reprogramming.

Chromosome instability is linked to tumor initiation and progression. Suv39h deficiency impairs H3K9 methylation at pericentromeric heterochromatin and leads to chromosome instability. For instance, Suv39h-deficient mice exhibit spontaneous B cell lymphoma and meiosis defects^35^. We found that the overexpression of Zfp296 protein reduced Suv39h1-mediated H3K9 di- and trimethylation *in vitro*. Therefore, Zfp296 appears to act as a negative regulator of chromosome stability, which may explain the tumorigenic potential of Zfp296 in acute leukemia. This possibility is supported by our finding that Zfp296 deficiency did not affect the frequency of spontaneous tumorigenesis and lifespan; however, the role of Zfp296 loss in the pathogenesis of oligodendroglioma and prostate carcinoma in which *Zfp296* is epigenetically silenced is still not known^19,20^. On the other hand, DNA double strand breaks (DSBs) can cause genome rearrangements and impair genomic stability. γH2AX, the phosphorylated form of H2AX, is regarded as a central component of the damaged chromatin^42^, and DNA repair protein MDC1 is known to directly bind γH2AX to regulate responses to DBSs^43^. HP1 also accumulates at DNA damage sites^44–46^. In this study, we showed significant binding of Zfp296 to H2AX, MDC1, and HP1 (Table S1). Its binding to other DNA repair proteins such as MSH6, DDB1, and LIG3 was also shown. These interactions may suggest a possible role of Zfp296 in DNA repair and shed light on the mechanisms of *Zfp296*-associated tumorigenesis. The suppression of H3K9 methyltransferases, such as Suv39h1, Eset, and G9a, promotes transcription factor accessibility and increases reprogramming efficiency^47–49^. Thus, Zfp296 might accelerate iPS reprogramming through the repression of H3K9 methylation.

In conclusion, we demonstrated that Zfp296 has a pivotal role in germ-cell development and embryonic growth. We also showed that Zfp296 functions as a chromatin effector. The epigenetic regulation mediated by Zfp296 is also likely to play important roles in cancer pathogenesis and the induction of pluripotency as well as in embryogenesis.

## Methods

### Targeted disruption of the *Zfp296* locus

The genomic region containing the *Zfp296* gene was amplified by long PCR and cloned into a plasmid. The targeting vector was designed to insert an IRES-βgeo-pA cassette into exon 3 of the *Zfp296* gene, which encodes all of the zinc-finger domains. The *Zfp296* targeting vector was linearized and electroporated into EB3 embryonic stem cells (129/Sv)^50^, and selection was performed with G418. Genomic DNA from G418-resistant colonies was analyzed for targeted disruption of the *Zfp296* gene by long PCR. The targeted ES cells were injected into C57BL/6 blastocysts to generate chimeras. The resulting chimeric mice were bred with C57BL/6 mice, and germline transmission of the knockout allele was confirmed by PCR of the genomic DNA from tail tips. Heterozygous mice were backcrossed to C57BL/6 mice. Our studies used mice obtained after backcrossing for at least six generations. To stage the embryonic age, noon on the day of vaginal plug detection was defined as embryonic day 0.5 (E0.5). Mice were housed and maintained in a controlled environment according to the institutional guidelines. All animal experiments were performed in accordance with the institutional guidelines (protocols #21–089 and #26–066), which were reviewed and approved by the Animal Care and Use Committee of the Osaka University Graduate School of Medicine. Mice were euthanized with an intraperitoneal injection of pentobarbital sodium at 180 mg/kg body weight.

### Genotyping PCR

Genotypes were determined by PCR analysis of the genomic DNA isolated from tail-biopsies or embryonic fragments (yolk sac, head, or tail). The gene-specific primers are listed in Table S2. The PCR conditions were as follows: initial denaturation 5 min at 95 °C, followed by 30–35 cycles of denaturation 30 sec at 94 °C, annealing 30 sec at 64 °C, and extension 1 min at 72 °C. PCR products were separated by electrophoresis in a 2% agarose gel and visualized by ethidium bromide staining.

### Antibody generation

Rabbits were immunized with a synthetic peptide corresponding to the C-terminal region of Zfp296 (amino acid residues 432 to 445: TLDKHLRQKHPEMA). Antisera were collected and then affinity-purified with an agarose column coupled with the immunizing peptide.

### Western blot analysis

Samples (cultured cells or whole embryos) were lysed in lysis buffer containing 50 mM Tris-HCl (pH 6.8), 2% SDS, and 10% glycerol supplemented with protease inhibitor cocktail (Calbiochem), followed by sonication. After centrifugation and protein quantification, the lysates were boiled for 5 min, loaded onto a 10-15% polyacrylamide Tris-glycine gel, separated by electrophoresis, and transferred onto a polyvinylidene difluoride (PVDF) membrane (Millipore). After washing, the membranes were blocked with 3% (w/v) skim milk in Tris-buffered saline (TBS) with 0.1% Tween-20 (TBST) or Blocking One (Nacalai) for 1 h at room temperature, and then incubated with primary antibodies in TBST for 1 h at room temperature. Primary antibodies were detected with horseradish peroxidase-conjugated secondary antibodies for 1 h at room temperature. The blot was developed using ECL or ECL Prime detection reagents (GE Healthcare), and visualized using a luminescent image analyzer LAS-4000 (Fujifilm) or X-ray film. For quantification, signal intensities on Western blots were measured with an LAS4000 imager and the instrument’s software. Antibodies and their dilutions are listed in Table S3.

### Immunofluorescence and histological analysis

Embryonic tissues (whole embryo and genital ridge) were fixed with 2% paraformaldehyde (PFA) in PBS for 2 h at 4 °C. Postnatal tissues (testis and ovary) were fixed with 4% PFA in PBS overnight at 4 °C. After washing, the tissues were incubated in 10% (w/w) sucrose in PBS for 1 h at 4 °C, and then transferred to 20% sucrose in PBS and incubated overnight at 4 °C. The tissues were frozen in OCT compound (Sakura), and then 10-μm-thick sections were cut, adhered onto MAS-coated slides (Matsunami), and rehydrated before staining. For immunofluorescence staining with anti-Zfp296 antibody, antigen retrieval was performed using Target Retrieval Solution pH 6.1 (DAKO). The sections were blocked in 10% goat or donkey serum plus 3% bovine serum albumin (BSA) in PBS for 1 h at room temperature, and then incubated with primary antibodies overnight at 4 °C. The sections were then incubated with Alexa Fluor-conjugated secondary antibodies for 1 h at room temperature. The slides were counterstained with 1 μg/ml DAPI (Molecular Probes) for 1 h at 4 °C prior to mounting. For histological analysis, tissues were fixed in 4% PFA overnight at 4 °C and then embedded in paraffin. The tissues were cut into 5-μm-thick sections and stained with hematoxylin and eosin. The antibodies and their dilutions are listed in Table S3. All imaging was performed using an FV1000 confocal microscope (Olympus) or a BZ-9000 multifunctional microscope (Keyence).

### Alkaline phosphatase staining and whole-mount *in situ* hybridization

Embryos were fixed with 4% PFA in PBS and, after washing three times with PBS, were stained using an alkaline phosphatase detection kit (Sigma) according to the manufacturer’s instructions. Whole-mount *in situ* hybridization was performed as described^51^. Digoxigenin-UTP-labeled riboprobes for the 3′UTR sequence of *Zfp296* were synthesized according to the manufacturer’s instructions (Roche).

### qRT-PCR

RNA was extracted from whole embryos using an RNeasy Mini kit (Qiagen) according to the manufacturer’s instructions. The RNA was then used for cDNA synthesis using ReverTraAce-α (Toyobo) and dT_20_ primer. Quantitative PCR analyses were performed using a DNA Master SYBR Green kit (Roche) and transcript-specific primer pairs (Table S2). Reactions were run on a Step One Plus real-time PCR system (Applied Biosystems). Each sample was analyzed in triplicate. Expression levels were determined by a standard curve for each primer pair and normalized to the level of the housekeeping gene *Tbp*.

### Plasmids, cell culture, and transfection

To construct expression plasmids, cDNAs were amplified by RT-PCR and cloned into the pCAG-IRES-puro vector^52^. The cDNA inserts were verified by sequencing. The resulting constructs were as follows: pCAG-Flag-Zfp296, pCAG-Flag-Zfp296 ΔZinc6, pCAG-Flag-Zfp296 ΔZinc4-6, pCAG-Flag-Zfp296 ΔZinc2-3, pCAG-Zfp296-GFP, pCAG-ZNF296, pCAG-ZNF296-GFP, pCAG-Myc-SUV39H1, pCAG-SUV39H1-DsRed, and pCAG-EGFP. HEK293T cells and primary MEF cells were maintained in high glucose DMEM (Sigma) supplemented with 10% FBS. Cells were grown to 80% confluency and transfected using Polyethylenimine Max (Polysciences) or Lipofectamine 2000 (Invitrogen) following the manufacturer’s instructions. After 44-48 h, the cells were used for experiments.

### GST pull-down experiments and mass spectrometry

Recombinant GST-Zfp296 or GST proteins were purified in hypertonic buffer using glutathione magnetic beads (Pierce). EB3 mouse embryonic stem cells were harvested with a cell scraper (Sumilon) into hypotonic buffer containing 10 mM Tris-HCl (pH 7.4), 10 mM NaCl, 3 mM MgCl_2_, and 0.1% NP-40 supplemented with protease inhibitor cocktail (Calbiochem). After vortexing briefly, the lysates were left on ice for 10 min, and then centrifuged at 14,000 g for 10 min at 4 °C. The supernatant was removed, and the pellet was resuspended in high-salt buffer containing 20 mM Tris-HCl (pH 8.0), 500 mM NaCl, 3 mM MgCl_2_, and 0.5% NP-40 supplemented with 100 U/ml Cryonase (cold-active nuclease; Takara) and protease inhibitor cocktail. After vortexing briefly, the lysates were incubated for 20 min at 4 °C for nuclease digestion, and then sonicated on ice using a Sonifier 250 (Branson) with a microtip. The sonication conditions were as follows: power level 2 for two 10-sec pulses, with 2-min rests on ice between pulses. After centrifugation to remove debris, the supernatant was collected and then diluted 3-fold in buffer containing 20 mM Tris-HCl (pH 8.0) supplemented with protease inhibitor cocktail. The lysates were incubated with purified recombinant GST-Zfp296 or GST proteins bound to glutathione magnetic beads overnight while rotating at 4 °C. The beads were washed three times with PBS plus 0.1% Tween-20 supplemented with protease inhibitor cocktail. The beads were then incubated in 50 mM Tris-HCl (pH 6.8), 2% sodium deoxycholate (SDC), 10% glycerol for 1 h while rocking at room temperature. Proteins were processed for LC-MS/MS analysis according to a standard protocol^53^. LC-MS/MS analysis was performed by an UltiMate 3000 Nano LC system (Thermo Fisher Scientific) coupled to a Q-Exactive hybrid quadrupole-Orbitrap mass spectrometer (Thermo Fisher Scientific).

### Chromatin immunoprecipitation

Embryos were dissociated with a solution containing 0.15% trypsin and 0.7 mM EDTA for 20 min while rocking at 37 °C and then diluted 5-fold in DMEM (Sigma) supplemented with 20% FBS to stop the trypsinization. After washing with PBS, the cells were crosslinked with 1% formaldehyde (Sigma) in PBS for 10 min while rocking at room temperature. Crosslinking was stopped by adding glycine solution to a final concentration of 125 mM. Subsequently, the chromatin preparation, immunoprecipitation, and DNA purification were performed using a SimpleChIP Enzymatic Chromatin IP kit (Cell Signaling). ChIP enrichment was determined by qPCR using major satellite primers^54,55^. The antibodies and primers are presented in the Supplementary Methods.

## Electronic supplementary material

Supplementary information
