# Supplementary material for: Zfp296 negatively regulates H3K9 methylation in embryonic development as a component of heterochromatin
Source: Sci Rep. 2017 Sep 29;7:12462. doi: 10.1038/s41598-017-12772-y (PMC5622089; doi:10.1038/s41598-017-12772-y)
Supplement: Supplementary file 1 — Supplementary information [file 41598_2017_12772_MOESM1_ESM.pdf]

Supplementary Information

Zfp296 negatively regulates H3K9 methylation in embryonic development as a component of heterochromatin

Takumi Matsuura, Satsuki Miyazaki, Tatsushi Miyazaki, Fumi Tashiro, Jun-ichi Miyazaki

Division of Stem Cell Regulation Research, Osaka University Graduate School of Medicine, 2-2 Yamadaoka, Suita, Osaka, 565-0871

Figure S1.

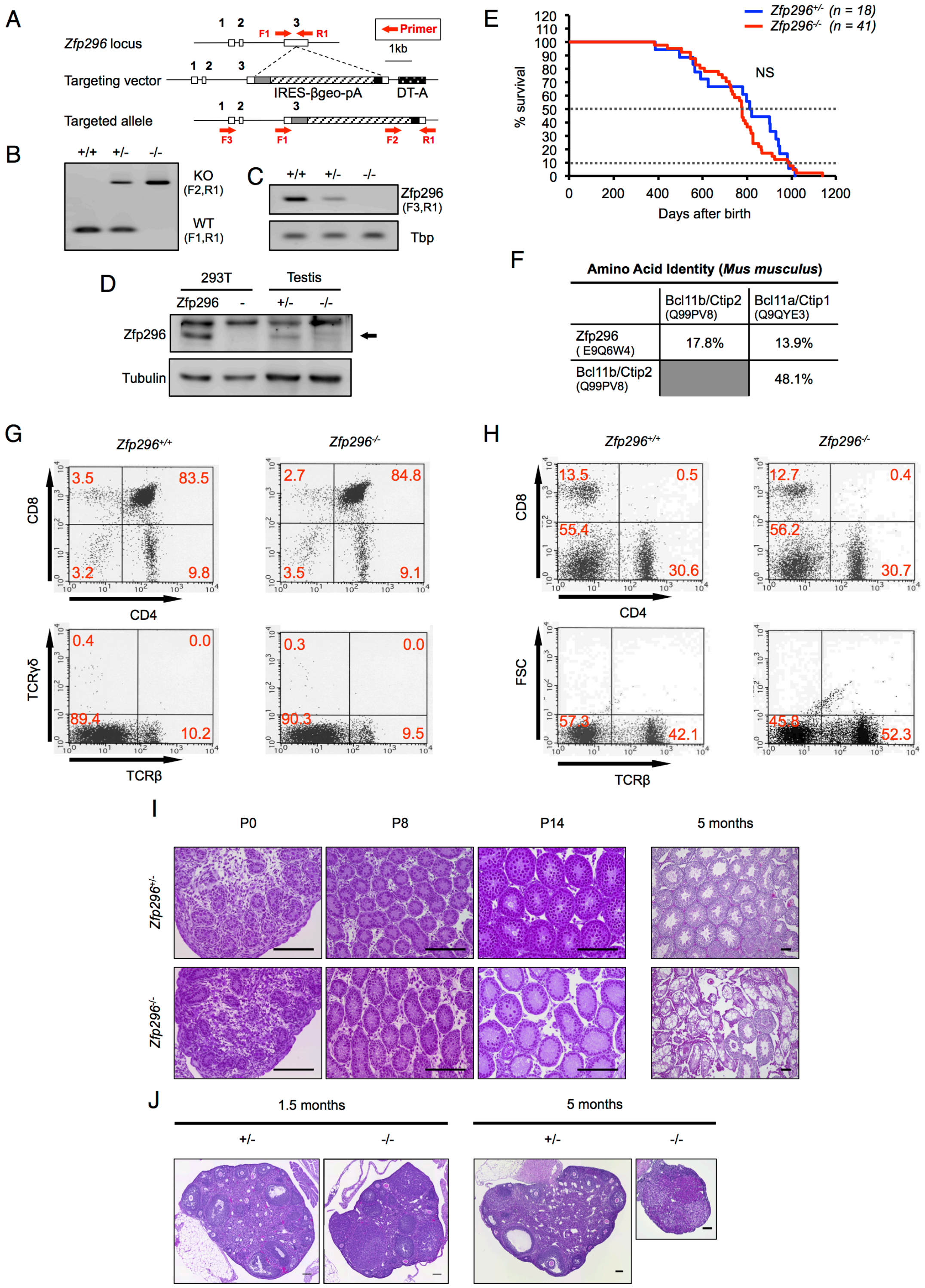

Figure S1 (Related to Figure 1).

(A) Schematic diagram of the *Zfp296* gene locus, the targeting vector, and the targeted allele. Positions of primers for PCR and RT-PCR analyses (B and C) are indicated.

(B) Genotyping by PCR of genomic DNA extracted from tail tips of 1 month-old mice. WT = 0.2 kbp, KO = 0.7 kbp.

(C) Detection of *Zfp296* mRNA in E9.75 littermate embryos by RT-PCR. PCR product from Tbp mRNA is shown as an internal control. Zfp296 = 0.6 kbp.

(D) Western blot showing the absence of Zfp296 protein in the adult testis from a *Zfp296*$^{-/-}$ mouse. Antibody specificity was confirmed using lysates from HEK293T cells transfected with pCAG-Zfp296, a plasmid vector expressing Zfp296. Tubulin served as a loading control.

(E) Kaplan-Meier survival curves of *Zfp296*$^{+/-}$ and *Zfp296*$^{-/-}$ mice (combined male and female). NS: no significant difference.

(F) Percent identity of amino acid sequences among Zfp296, Bcl11b/Ctip2, and Bcl11a/Ctip1. Alignment was calculated with Clustal Omega.

(G and H) Flow cytometry analysis of the CD4, CD8, TCRβ, and TCRγδ expression on thymocytes (G), and the CD4, CD8, and TCRβ on splenocytes (H) from 4-month-old *Zfp296*$^{-/-}$ and *Zfp296*$^{+/+}$ mice.

(I and J)Hematoxylin and eosin staining of testis (I) and ovary (J) sections from *Zfp296*$^{-/-}$ and control mice at various postnatal stages. Scale bar is 100 μm.

Figure S2.

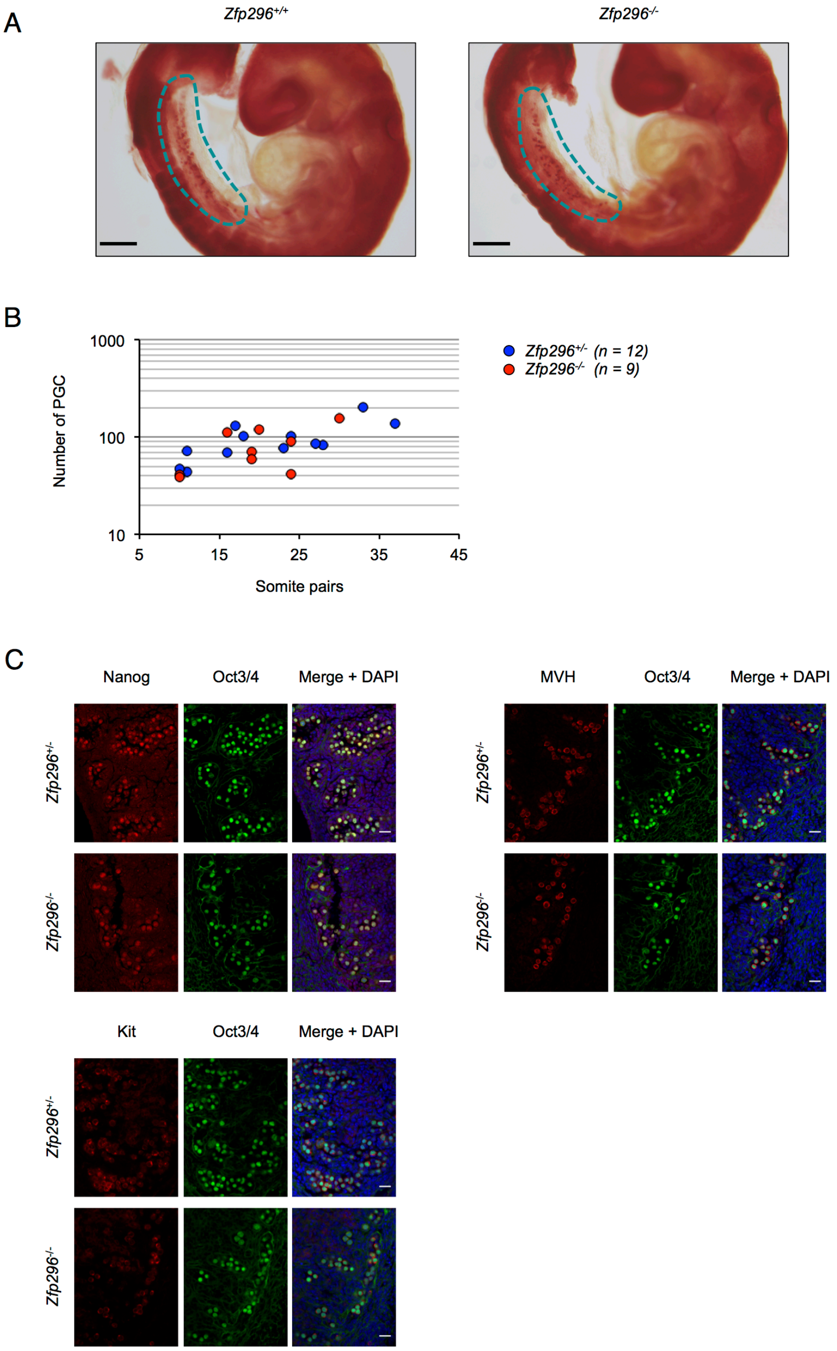

Figure S2 (Related to Figure 2).

(A) Alkaline phosphatase (AP) staining of E9.5 *Zfp296*$^{+/+}$ and *Zfp296*$^{-/-}$ embryos. Dashed circle indicates the hindgut region containing PGCs.

(B) Number of AP-positive PGCs at different developmental stages.

(C) Immunofluorescence staining of E13.5 *Zfp296*$^{+/-}$ and *Zfp296*$^{-/-}$ embryonic testis and ovary sections for the indicated PGC markers. Scale bar is 20 μm.

Figure S3.

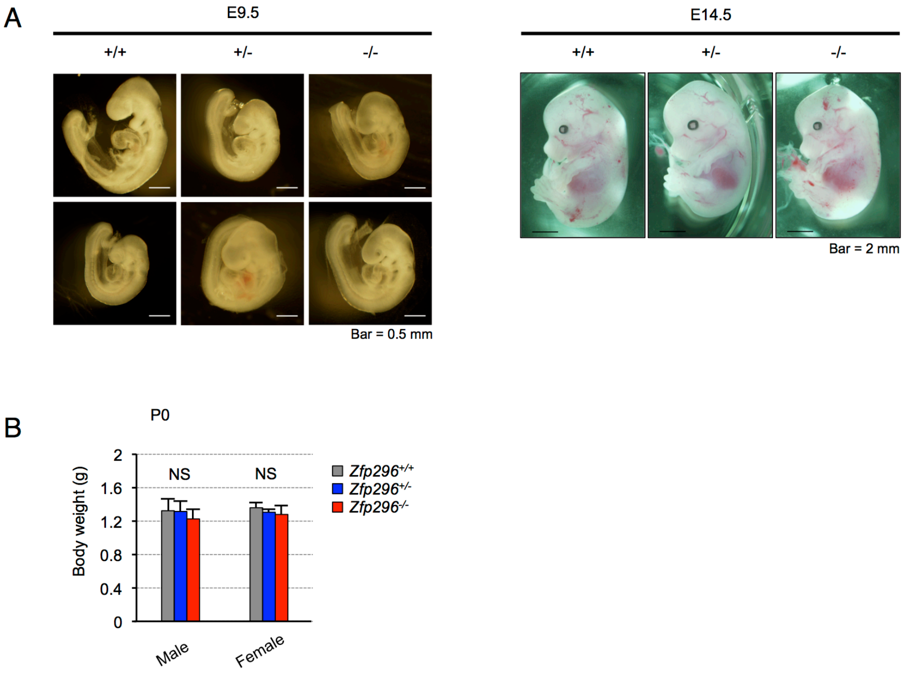

Figure S3 (Related to Figure 3).

(A) Gross images of E9.5 and E14.5 *Zfp296*$^{-/-}$ and control embryos from a single litter of *Zfp296*$^{-/+}$ intercrosses. We observed thirty-eight E9.5 embryos (+/+ : +/- : -/- = 10 : 16 : 12) and twenty E14.5 embryos (+/+ : +/- : -/- = 8 : 10 : 2) in total.

(B) Body weight of *Zfp296*$^{-/-}$ and control mice at P0. NS: no significant difference.

Figure S4.

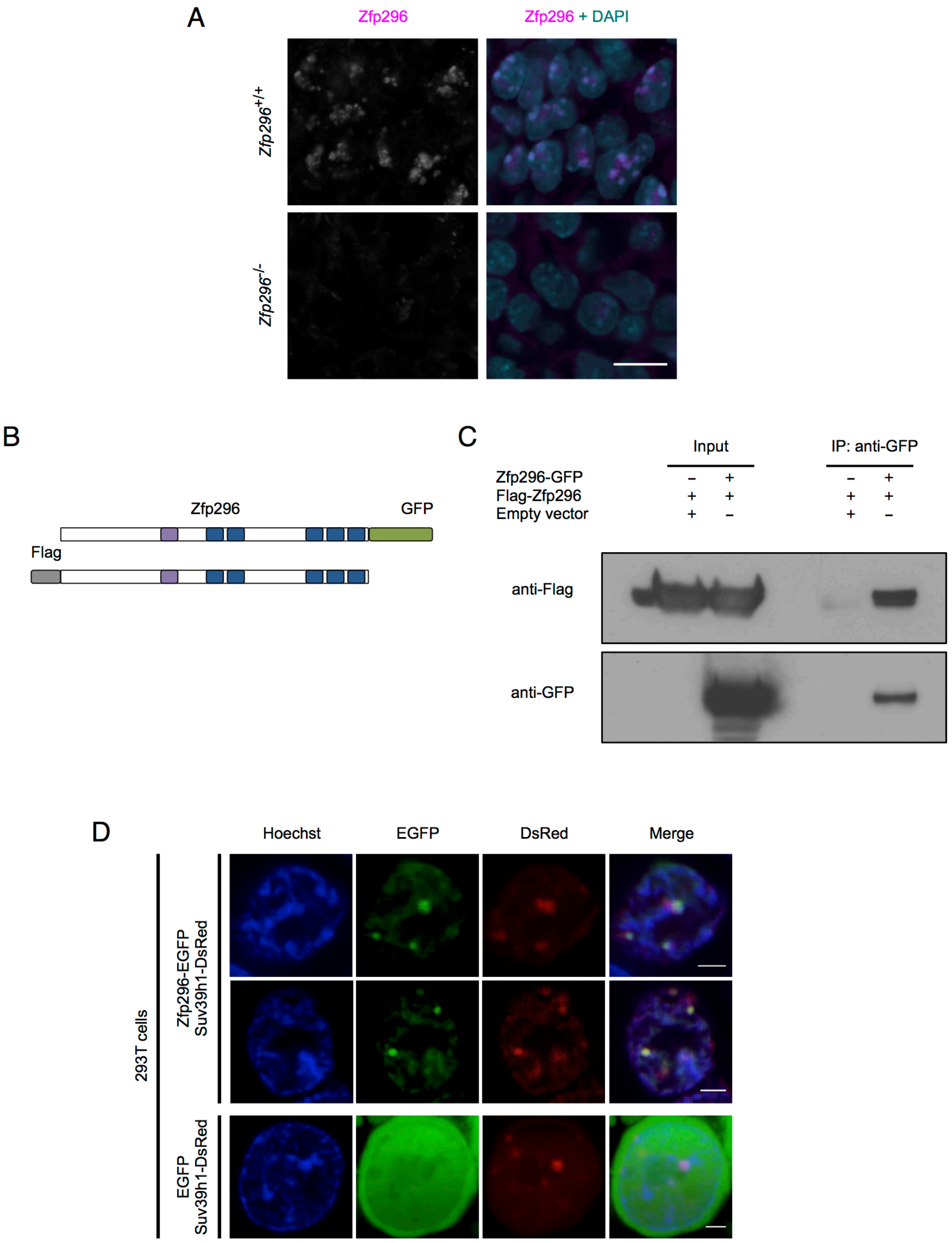

Figure S4 (Related to Figure 4).

(A) Immunofluorescence staining of E9.75 wild-type and Zfp296-/- embryos for Zfp296. Nuclei were counterstained with DAPI. Scale bar = 10 μm.

(B) Schematic diagram of Zfp296-GFP and Flag-Zfp296.

(C) Coimmunoprecipitation (IP) analysis of epitope-tagged proteins in HEK293T cells. HEK293T cells were transiently transfected with the indicated pCAG expression constructs. Nuclear extracts from transfected HEK293T cells were subjected to co-IP, and the immunoprecipitated proteins were detected by Western

blot (WB). Co-IP and WB were performed with the indicated antibodies.

(D) Live-cell imaging of 293T cells expressing Zfp296-EGFP (or EGFP as a control) and Suv39h1-DsRed. DNA is counterstained with Hoechst. Scale bar is 2 μm.

Figure S5.

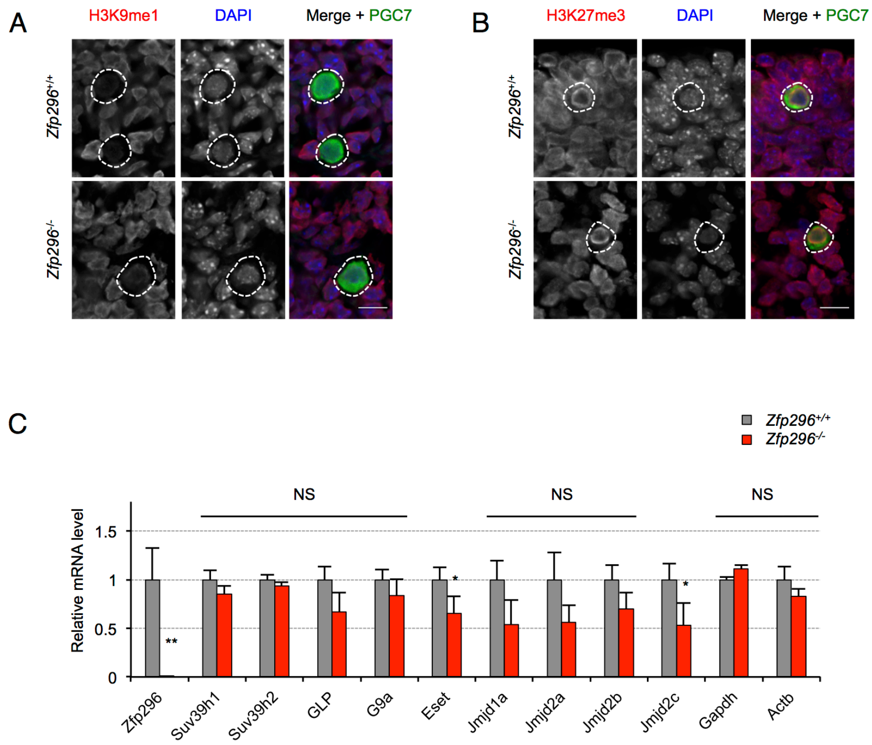

Figure S5 (Related to Figure 5).

(A and B) Immunofluorescence staining of E9.75 *Zfp296*$^{-/-}$ and *Zfp296*$^{+/+}$ whole-embryo sections for H3K9me1 (A) and H3K27me3 (B). PGC7 served as a marker for primordial germ cells.

(C) Relative mRNA levels of H3K9 methylation-related genes in E9.75 *Zfp296*$^{-/-}$ and wild-type embryos assessed by qRT-PCR; mRNA levels were normalized to that of *Tbp*. Data represent mean ± SD from three pairs of littermate embryos. *Gapdh* and *Actb* are shown as internal housekeeping control genes. **p < 0.01 *p < 0.05 by two-tailed Student's *t*-test.

Supplementary Methods

Flow cytometry

Single-cell suspensions from the thymus and spleen were prepared as previously described[26], and 1 x 10^6 cells were incubated with antibodies in PBS plus 1% FBS and 0.05% NaN_3 for 20 min at 4°C. After washing with PBS plus 1% FBS, the cells were resuspended in the same buffer, and analyzed using a FACSAria (BD Biosciences). Antibodies and their dilutions are listed in Table S3.

Co-immunoprecipitation

HEK293T cells were co-transfected with plasmid constructs as described in Methods ("Plasmids, cell culture, and transfection"). After 44-48 h, the cells were harvested, and nuclear extracts were prepared as described in Methods ("GST pull-down experiments and mass spectrometry"). The lysates were incubated with antibody for 1 h at 4°C and then mixed with Dynabeads Protein G (Invitrogen) for 3 h while rotating at 4°C. The beads were washed three times with buffer containing 20 mM Tris-HCl (pH 8.0), 125 mM NaCl, 0.05% NP-40, and 10% Blocking One (Nacalai) supplemented with protease inhibitor cocktail. The beads were then boiled in 62.5 mM Tris-HCl (pH 6.8), 2% SDS, 10% Glycerol, and 0.01% phenol red for 5 min. The eluted proteins were analyzed by Western blot as described in Methods ("Western blot analysis"). The antibodies and their dilutions are listed in Table S3.

Live cell imaging

HEK293T cells were seeded on collagen-coated glass-bottom dishes (Matsunami) and then transfected as described in Methods ("Plasmids, cell culture, and transfection"). The cells were stained with 5 μg/ml Hoechst 33342 (Sigma) in medium for 30 min at 37°C, followed by confocal microscope analysis.

Table S1.

Identified Proteins (991)	Accession Number	Molecular Weight	Total Spectrum Count				Quantitative Value (calculated with Scaffold software)				Fold Change	Fisher's Exact Test (p-value)
			Control		Pull Down		Control		Pull Down			
			#1	#2	#1	#2	#1	#2	#1	#2		
Protein Zfp296 OS=Mus musculus GN=Zfp296 PE=1 SV=1	E9Q6W4	48 kDa	0	3	59	184	2	4	68	111	30	< 0.00010
Protein Mki67 OS=Mus musculus GN=Mki67 PE=1 SV=1	E9PVX6	351 kDa	0	0	49	68	2	2	56	41	24	< 0.00010
Basement membrane-specific heparan sulfate proteoglycan core protein OS=Mus musculus GN=Hspg2 PE=4 SV=1	E9PZ16	470 kDa	0	0	32	33	2	2	37	20	14	< 0.00010
Protein ELYS OS=Mus musculus GN=Ahctf1 PE=1 SV=1	Q8CJF7	248 kDa	0	0	14	38	2	2	16	23	9.8	< 0.00010
Tyrosine-protein kinase BAZ1B OS=Mus musculus GN=Baz1b PE=1 SV=2	Q9Z277	171 kDa	0	0	13	32	2	2	15	19	8.6	< 0.00010
Histone H1.4 OS=Mus musculus GN=Hist1h1e PE=1 SV=2	P43274	22 kDa	0	0	16	25	2	2	18	15	8.4	< 0.00010
RNA cytidine acetyltransferase OS=Mus musculus GN=Nat10 PE=1 SV=1	Q8K224	115 kDa	3	2	26	41	4	3	30	25	8	< 0.00010
ATP-dependent RNA helicase DDX18 OS=Mus musculus GN=Ddx18 PE=1 SV=1	Q8K363	74 kDa	5	0	39	45	7	2	45	27	7.9	< 0.00010
DNA topoisomerase 1 OS=Mus musculus GN=Top1 PE=1 SV=2	Q04750	91 kDa	0	2	20	17	2	3	23	10	7.2	< 0.00010
HEAT repeat containing 1 OS=Mus musculus GN=Heatr1 PE=1 SV=1	G3X9B1	242 kDa	5	1	30	50	7	2	34	30	7.1	< 0.00010
Histone H2AX OS=Mus musculus GN=H2afx PE=1 SV=2	P27661	15 kDa	0	0	0	44	2	2	2	27	7.1	< 0.00010
Fibronectin OS=Mus musculus GN=Fn1 PE=1 SV=1	A0A087WR50 (+3)	263 kDa	0	0	15	18	2	2	17	11	7	< 0.00010
Nucleolar GTP-binding protein 1 OS=Mus musculus GN=Gtpbp4 PE=1 SV=3	Q99ME9	74 kDa	0	0	12	21	2	2	14	13	6.6	< 0.00010
Chromobox protein homolog 1 OS=Mus musculus GN=Cbx1 PE=1 SV=1	P83917	21 kDa	0	0	14	15	2	2	16	9	6.3	< 0.00010
Putative helicase MOV-10 OS=Mus musculus GN=Mov10 PE=1 SV=2	D3YVL0 (+2)	114 kDa	2	0	15	21	3	2	17	13	6.2	< 0.00010
WD repeat-containing protein 3 OS=Mus musculus GN=Wdr3 PE=1 SV=1	Q8BHB4	106 kDa	0	1	10	22	2	2	11	13	6.2	< 0.00010
Myosin-9 OS=Mus musculus GN=Myh9 PE=1 SV=4	Q8VDD5	226 kDa	0	0	8	26	2	2	9	16	6.2	< 0.00010
Transcriptional repressor p66 alpha OS=Mus musculus GN=Gatad2a PE=1 SV=1	E9QMN5 (+1)	67 kDa	0	0	12	18	2	2	14	11	6.2	< 0.00010
Protein Numa1 OS=Mus musculus GN=Numa1 PE=1 SV=1	E9Q7G0	236 kDa	5	5	36	68	7	6	41	41	6.1	< 0.00010
Nuclear valosin-containing protein-like OS=Mus musculus GN=Nvl PE=1 SV=1	Q9DBY8	94 kDa	2	0	13	23	3	2	15	14	5.9	< 0.00010
Transcriptional regulator ATRX OS=Mus musculus GN=Atrx PE=1 SV=3	Q61687	279 kDa	0	0	12	14	2	2	14	8	5.6	< 0.00010
DNA mismatch repair protein Msh6 OS=Mus musculus GN=Msh6 PE=1 SV=3	P54276	151 kDa	0	0	8	20	2	2	9	12	5.3	< 0.00010
ATP-dependent RNA helicase DDX51 OS=Mus musculus GN=Ddx51 PE=1 SV=1	Q6P9R1	70 kDa	0	0	9	18	2	2	10	11	5.3	< 0.00010
Core histone macro-H2A.1 OS=Mus musculus GN=H2afy PE=1 SV=3	Q9QZQ8	40 kDa	0	0	8	20	2	2	9	12	5.3	< 0.00010
Pescadillo homolog OS=Mus musculus GN=Pes1 PE=1 SV=1	Q5SQ20 (+1)	68 kDa	0	0	6	23	2	2	7	14	5.2	< 0.00010
Scaffold attachment factor B1 OS=Mus musculus GN=Safb PE=1 SV=2	D3YXK2 (+1)	105 kDa	0	0	9	17	2	2	10	10	5.1	< 0.00010
Probable RNA-binding protein 19 OS=Mus musculus GN=Rbm19 PE=1 SV=1	Q8R3C6	106 kDa	0	0	10	15	2	2	11	9	5.1	< 0.00010
Chromobox protein homolog 5 OS=Mus musculus GN=Cbx5 PE=1 SV=1	Q61686	22 kDa	0	0	9	16	2	2	10	10	5	< 0.00010
Heterogeneous nuclear ribonucleoprotein R OS=Mus musculus GN=Hnrnpr PE=1 SV=1	Q8VHM5	71 kDa	0	0	8	18	2	2	9	11	5	< 0.00010
Heat shock protein HSP 90-beta OS=Mus musculus GN=Hsp90ab1 PE=1 SV=3	P11499	83 kDa	0	0	12	10	2	2	14	6	5	< 0.00010
RNA-binding protein 28 OS=Mus musculus GN=Rbm28 PE=1 SV=4	Q8CGC6	84 kDa	0	2	9	20	2	3	10	12	4.9	0.00012
Myosin-10 OS=Mus musculus GN=Myh10 PE=1 SV=1	Q3UH59 (+2)	233 kDa	0	0	5	23	2	2	6	14	4.9	< 0.00010
Protein LYRIC OS=Mus musculus GN=Mtdh PE=1 SV=1	Q80WJ7	64 kDa	0	0	13	8	2	2	15	5	4.9	< 0.00010
DNA topoisomerase 2-beta OS=Mus musculus GN=Top2b PE=1 SV=2	Q64511	182 kDa	0	0	8	17	2	2	9	10	4.9	< 0.00010
Kinesin-like protein KIF20B OS=Mus musculus GN=Kif20b PE=1 SV=3	Q80WE4	204 kDa	0	0	10	13	2	2	11	8	4.8	< 0.00010
DNA damage-binding protein 1 OS=Mus musculus GN=Ddb1 PE=1 SV=2	Q3U1J4	127 kDa	0	0	12	8	2	2	14	5	4.7	0.0001
Protein Wdr75 OS=Mus musculus GN=Wdr75 PE=1 SV=1	Q3U821	94 kDa	0	0	8	16	2	2	9	10	4.7	< 0.00010
Protein RRP5 homolog OS=Mus musculus GN=Pdcd11 PE=1 SV=2	Q6NS46	208 kDa	5	6	29	59	7	8	33	36	4.6	< 0.00010
PH-interacting protein OS=Mus musculus GN=Phip PE=1 SV=1	F8VQ93 (+1)	207 kDa	0	0	6	19	2	2	7	11	4.6	< 0.00010
U3 small nucleolar RNA-associated protein 18 homolog OS=Mus musculus GN=Utp18 PE=1 SV=1	Q5SSI6	61 kDa	0	0	8	15	2	2	9	9	4.6	< 0.00010
Heterogeneous nuclear ribonucleoprotein D-like OS=Mus musculus GN=Hnrnpdl PE=1 SV=1	D3YTQ3	46 kDa	0	0	8	15	2	2	9	9	4.6	< 0.00010
Ribosome-binding protein 1 OS=Mus musculus GN=Rrbp1 PE=1 SV=1	A2AVJ7 (+1)	158 kDa	0	0	5	20	2	2	6	12	4.5	< 0.00010
Bromodomain adjacent to zinc finger domain protein 2A OS=Mus musculus GN=Baz2a PE=1 SV=1	E9Q374 (+1)	209 kDa	0	0	9	13	2	2	10	8	4.5	< 0.00010
Insulin-like growth factor 2 mRNA-binding protein 1 OS=Mus musculus GN=Igf2bp1 PE=1 SV=1	O88477	63 kDa	3	3	18	25	4	4	21	15	4.4	0.00012
Nucleolar protein 6 OS=Mus musculus GN=Nol6 PE=2 SV=2	Q8R5K4	129 kDa	4	0	14	30	6	2	16	18	4.4	< 0.00010
Importin subunit alpha-1 OS=Mus musculus GN=Kpna2 PE=1 SV=2	P52293	58 kDa	1	0	7	16	2	2	8	10	4.4	0.00024
Cell growth-regulating nucleolar protein OS=Mus musculus GN=Lyar PE=1 SV=2	Q08288	44 kDa	0	0	9	12	2	2	10	7	4.4	< 0.00010
Interleukin enhancer-binding factor 2 OS=Mus musculus GN=Ilf2 PE=1 SV=1	Q9CXY6	43 kDa	3	4	19	32	4	5	22	19	4.3	< 0.00010
ATP-dependent RNA helicase DDX54 OS=Mus musculus GN=Ddx54 PE=1 SV=1	Q8K4L0	98 kDa	3	0	11	24	4	2	13	14	4.3	< 0.00010
Protein Jade-1 OS=Mus musculus GN=Jade1 PE=1 SV=2	Q6ZPI0	94 kDa	0	0	6	17	2	2	7	10	4.3	< 0.00010
Transitional endoplasmic reticulum ATPase OS=Mus musculus GN=Vcp PE=1 SV=4	Q01853	89 kDa	0	0	9	11	2	2	10	7	4.2	0.0001
Ribosome biogenesis protein BOP1 OS=Mus musculus GN=Bop1 PE=1 SV=1	P97452	83 kDa	0	2	6	20	2	3	7	12	4.1	0.00038
Periodic tryptophan protein 1 homolog OS=Mus musculus GN=Pwp1 PE=1 SV=1	Q99LL5	56 kDa	0	0	9	10	2	2	10	6	4.1	0.00016
Nidogen-2 OS=Mus musculus GN=Nid2 PE=1 SV=2	O88322	154 kDa	0	0	11	6	2	2	13	4	4.1	0.00041
Heterogeneous nuclear ribonucleoprotein A/B OS=Mus musculus GN=Hnrnpab PE=1 SV=1	Q20BD0 (+2)	36 kDa	0	0	9	10	2	2	10	6	4.1	0.00016
DNA ligase OS=Mus musculus GN=Lig3 PE=1 SV=1	K4DI59 (+3)	113 kDa	0	0	5	17	2	2	6	10	4	< 0.00010
Probable ATP-dependent RNA helicase DDX27 OS=Mus musculus GN=Ddx27 PE=1 SV=3	Q921N6	86 kDa	0	5	15	26	2	6	17	16	3.9	< 0.00010
WD repeat-containing protein 46 OS=Mus musculus GN=Wdr46 PE=2 SV=1	Q9Z0H1	69 kDa	2	0	7	18	3	2	8	11	3.9	0.00056
CCAAT/enhancer-binding protein zeta OS=Mus musculus GN=Cebpz PE=1 SV=2	P53569	120 kDa	0	2	8	14	2	3	9	8	3.8	0.0018
Serine/arginine-rich splicing factor 3 OS=Mus musculus GN=Srsf3 PE=1 SV=1	P84104	19 kDa	0	0	7	12	2	2	8	7	3.8	0.00016
Agrin OS=Mus musculus GN=Agrn PE=1 SV=1	M0QWP1	217 kDa	0	0	9	8	2	2	10	5	3.8	0.00041
ATP-binding cassette sub-family F member 1 OS=Mus musculus GN=Abcf1 PE=1 SV=1	Q6P542	95 kDa	0	0	7	12	2	2	8	7	3.8	0.00016
Metastasis-associated protein MTA2 OS=Mus musculus GN=Mta2 PE=1 SV=1	Q9R190	75 kDa	0	0	7	12	2	2	8	7	3.8	0.00016
Histone H1.1 OS=Mus musculus GN=Hist1h1a PE=1 SV=2	P43275	22 kDa	0	0	7	12	2	2	8	7	3.8	0.00016
Nucleolar protein 10 OS=Mus musculus GN=Nol10 PE=2 SV=1	Q5RJG1	80 kDa	0	0	8	9	2	2	9	5	3.7	0.00041
Actin-like protein 6A OS=Mus musculus GN=Actl6a PE=1 SV=2	Q9Z2N8	47 kDa	0	2	10	9	2	3	11	5	3.7	0.0055
Pinin OS=Mus musculus GN=Pnn PE=1 SV=4	O35691 (+1)	82 kDa	0	0	6	13	2	2	7	8	3.7	0.00016
Pogo transposable element with ZNF domain OS=Mus musculus GN=Pogz PE=4 SV=1	D3YUW8 (+2)	145 kDa	0	0	8	9	2	2	9	5	3.7	0.00041
Protein Rbm15 OS=Mus musculus GN=Rbm15 PE=1 SV=1	Q0VBL3	106 kDa	0	0	7	11	2	2	8	7	3.7	0.00026
Replication factor C subunit 1 OS=Mus musculus GN=Rfc1 PE=1 SV=1	G3UWX1 (+3)	126 kDa	0	0	6	13	2	2	7	8	3.7	0.00016
Eukaryotic initiation factor 4A-I OS=Mus musculus GN=Eif4a1 PE=1 SV=1	P60843	46 kDa	0	0	7	11	2	2	8	7	3.7	0.00026
Kinesin-like protein KIF20A OS=Mus musculus GN=Kif20a PE=2 SV=1	P97329	100 kDa	0	0	5	15	2	2	6	9	3.7	0.0001
Serine/arginine-rich splicing factor 7 OS=Mus musculus GN=Srsf7 PE=1 SV=1	Q8BL97	31 kDa	0	0	6	13	2	2	7	8	3.7	0.00016
Poly [ADP-ribose] polymerase OS=Mus musculus GN=Parp1 PE=1 SV=1	Q921K2	113 kDa	7	7	28	61	10	9	32	37	3.6	< 0.00010
Nucleolar pre-ribosomal-associated protein 1 OS=Mus musculus GN=Urb1 PE=1 SV=1	E9PU96 (+1)	255 kDa	0	0	3	18	2	2	3	11	3.6	< 0.00010
U3 small nucleolar ribonucleoprotein protein MPP10 OS=Mus musculus GN=Mphosph10 PE=1 SV=2	Q810V0	79 kDa	0	0	4	16	2	2	5	10	3.6	0.0001
Lysine-specific demethylase 5B OS=Mus musculus GN=Kdm5b PE=1 SV=1	Q80Y84	176 kDa	0	0	7	10	2	2	8	6	3.5	0.00041
Protein Bptf OS=Mus musculus GN=Bptf PE=1 SV=1	A2A654 (+2)	333 kDa	0	0	7	10	2	2	8	6	3.5	0.00041
Deoxynucleotidyltransferase terminal-interacting protein 2 OS=Mus musculus GN=Dnttip2 PE=1 SV=1	Q8R2M2	84 kDa	0	0	6	12	2	2	7	7	3.5	0.00026
Cirhin OS=Mus musculus GN=Cirh1a PE=2 SV=3	Q8R2N2	77 kDa	0	0	6	12	2	2	7	7	3.5	0.00026
Nucleolar complex protein 3 homolog OS=Mus musculus GN=Noc3l PE=2 SV=2	Q8VI84	93 kDa	0	0	5	14	2	2	6	8	3.5	0.00016
MKI67 FHA domain-interacting nucleolar phosphoprotein OS=Mus musculus GN=Nifk PE=1 SV=1	Q91VE6	36 kDa	0	0	6	12	2	2	7	7	3.5	0.00026
Proliferation-associated protein 2G4 OS=Mus musculus GN=Pa2g4 PE=1 SV=3	P50580	44 kDa	0	0	8	8	2	2	9	5	3.5	0.00064
ATPase family AAA domain-containing protein 3 OS=Mus musculus GN=Atad3 PE=1 SV=1	Q925I1	67 kDa	0	0	6	12	2	2	7	7	3.5	0.00026
Origin recognition complex subunit 1 OS=Mus musculus GN=Orc1 PE=1 SV=2	Q9Z1N2	95 kDa	0	0	5	13	2	2	6	8	3.4	0.00026
40S ribosomal protein S6 OS=Mus musculus GN=Rps6 PE=1 SV=1	P62754	29 kDa	0	2	8	11	2	3	9	7	3.4	0.0055
Histone acetyltransferase KAT7 OS=Mus musculus GN=Kat7 PE=1 SV=1	Q5SVQ0	71 kDa	0	0	5	13	2	2	6	8	3.4	0.00026
Non-histone chromosomal protein HMG-17 OS=Mus musculus GN=Hmgn2 PE=1 SV=1	A3KGL9 (+2)	10 kDa	0	0	6	11	2	2	7	7	3.4	0.00041
DNA-directed RNA polymerase I subunit RPA34 OS=Mus musculus GN=Cd3eap PE=1 SV=2	Q76KJ5	43 kDa	0	0	8	7	2	2	9	4	3.4	0.001
RNA 3'-terminal phosphate cyclase-like protein OS=Mus musculus GN=Rcl1 PE=2 SV=1	Q9JJT0	41 kDa	0	0	4	15	2	2	5	9	3.4	0.00016
Dihydrolipoyllysine-residue succinyltransferase component of 2-oxoglutarate dehydrogenase complex, mitochondrial OS=Mus musculus GN=Dlst PE=1 SV=1	Q9D2G2	49 kDa	0	0	7	9	2	2	8	5	3.4	0.00064
Protein KRI1 homolog OS=Mus musculus GN=Kri1 PE=1 SV=2	F6WIU1	82 kDa	0	0	6	11	2	2	7	7	3.4	0.00041
Polyadenylate-binding protein 1 OS=Mus musculus GN=Pabpc1 PE=1 SV=2	P29341	71 kDa	0	3	7	18	2	4	8	11	3.2	0.0021
Caprin-1 OS=Mus musculus GN=Caprin1 PE=1 SV=2	Q60865	78 kDa	0	0	7	8	2	2	8	5	3.2	0.001
Putative ATP-dependent RNA helicase DHX30 OS=Mus musculus GN=Dhx30 PE=2 SV=1	Q99PU8	137 kDa	0	0	5	12	2	2	6	7	3.2	0.00041
Lamin-B receptor OS=Mus musculus GN=Lbr PE=1 SV=2	Q3U9G9	71 kDa	0	0	7	8	2	2	8	5	3.2	0.001
Nucleolar protein 11 OS=Mus musculus GN=Nol11 PE=2 SV=1	Q8BJW5	81 kDa	0	0	5	12	2	2	6	7	3.2	0.00041

Nucleolar protein 14 OS=Mus musculus GN=Nop14 PE=1 SV=2	Q8R3N1	99 kDa	0	0	6	10	2	2	7	6	3.2	0.00064
Protein SON OS=Mus musculus GN=Son PE=1 SV=1	H9KV00 (+1)	269 kDa	0	0	8	6	2	2	9	4	3.2	0.0016
Rcc1 protein OS=Mus musculus GN=Rcc1 PE=1 SV=1	Q6PFB2 (+1)	46 kDa	0	0	6	10	2	2	7	6	3.2	0.00064
Staphylococcal nuclease domain-containing protein 1 OS=Mus musculus GN=Snd1 PE=1 SV=1	Q78PY7	102 kDa	0	0	7	8	2	2	8	5	3.2	0.001
Scaffold attachment factor B2 OS=Mus musculus GN=Safb2 PE=1 SV=2	Q80YR5	112 kDa	0	0	6	10	2	2	7	6	3.2	0.00064
RNA-binding protein Raly (Fragment) OS=Mus musculus GN=Raly PE=1 SV=1	A2AU62 (+1)	32 kDa	3	2	13	10	4	3	15	6	3.1	0.025
Ribosome biogenesis regulatory protein homolog OS=Mus musculus GN=Rrs1 PE=1 SV=1	Q9CYH6	42 kDa	0	0	5	11	2	2	6	7	3.1	0.00064
BMS1 homolog, ribosome assembly protein (Yeast) OS=Mus musculus GN=Bms1 PE=1 SV=1	Q6PGF5	145 kDa	0	0	4	13	2	2	5	8	3.1	0.00041
eIF-2-alpha kinase activator GCN1 OS=Mus musculus GN=Gcn1 PE=1 SV=1	E9PVA8	293 kDa	0	0	5	11	2	2	6	7	3.1	0.00064
Probable rRNA-processing protein EBP2 OS=Mus musculus GN=Ebna1bp2 PE=2 SV=1	Q9D903	35 kDa	0	0	6	9	2	2	7	5	3.1	0.001
MCG2065, isoform CRA_c OS=Mus musculus GN=Gltscr2 PE=1 SV=1	Q8BK35	56 kDa	0	0	7	7	2	2	8	4	3.1	0.0016
60S ribosomal protein L4 OS=Mus musculus GN=Rpl4 PE=1 SV=3	Q9D8E6	47 kDa	5	6	22	31	7	8	25	19	3	0.0005
Protein Nolc1 OS=Mus musculus GN=Nolc1 PE=1 SV=1	E9Q5C9	74 kDa	5	3	17	22	7	4	20	13	3	0.0026
60S ribosomal protein L7a OS=Mus musculus GN=Rpl7a PE=1 SV=2	P12970	30 kDa	6	2	14	28	9	3	16	17	3	0.0011
Heat shock protein HSP 90-alpha OS=Mus musculus GN=Hsp90aa1 PE=1 SV=4	P07901	85 kDa	4	0	13	14	6	2	15	8	3	0.0031
DNA (cytosine-5)-methyltransferase 3B OS=Mus musculus GN=Dnmt3b PE=2 SV=1	Q3KR45	97 kDa	4	0	10	19	6	2	11	11	3	0.0016
DEAH (Asp-Glu-Ala-His) box polypeptide 37 OS=Mus musculus GN=Dhx37 PE=1 SV=1	Q6NZL1	128 kDa	0	0	4	12	2	2	5	7	3	0.00064
DNA (cytosine-5)-methyltransferase 1 OS=Mus musculus GN=Dnmt1 PE=1 SV=5	P13864	183 kDa	7	2	17	29	10	3	20	18	2.9	0.00076
Ribosomal RNA processing protein 1 homolog B OS=Mus musculus GN=Rrp1b PE=1 SV=2	Q91YK2	81 kDa	3	4	14	19	4	5	16	11	2.9	0.0066
Protein Ogdhl OS=Mus musculus GN=Ogdhl PE=1 SV=1	E9Q7L0	117 kDa	0	0	5	10	2	2	6	6	2.9	0.001
WD repeat-containing protein 5 OS=Mus musculus GN=Wdr5 PE=1 SV=1	P61965	37 kDa	0	2	7	9	2	3	8	5	2.9	0.016
DNA replication licensing factor MCM7 OS=Mus musculus GN=Mcm7 PE=1 SV=1	Q61881	81 kDa	0	0	6	8	2	2	7	5	2.9	0.0016
Laminin subunit alpha-5 OS=Mus musculus GN=Lama5 PE=1 SV=4	Q61001	404 kDa	0	0	8	4	2	2	9	2	2.9	0.004
ESF1 homolog OS=Mus musculus GN=Esf1 PE=1 SV=1	Q3V1V3	98 kDa	0	0	6	8	2	2	7	5	2.9	0.0016
Eukaryotic translation initiation factor 2 subunit 1 OS=Mus musculus GN=Eif2s1 PE=1 SV=3	Q6ZWX6	36 kDa	0	0	7	6	2	2	8	4	2.9	0.0026
Transcriptional repressor p66-beta OS=Mus musculus GN=Gatad2b PE=1 SV=1	Q8VHR5	65 kDa	0	0	7	6	2	2	8	4	2.9	0.0026
Pumilio domain-containing protein KIAA0020 OS=Mus musculus GN=Kiaa0020 PE=1 SV=2	Q8BKS9	73 kDa	4	3	9	27	6	4	10	16	2.8	0.0028
Suppressor of SWI4 1 homolog OS=Mus musculus GN=Ppan PE=1 SV=2	Q91YU8	53 kDa	0	3	8	12	2	4	9	7	2.8	0.012
Ras GTPase-activating protein-binding protein 1 OS=Mus musculus GN=G3bp1 PE=1 SV=1	P97855	52 kDa	0	0	5	9	2	2	6	5	2.8	0.0016
Ribosomal RNA processing protein 1 homolog A OS=Mus musculus GN=Rrp1 PE=1 SV=2	P56183	55 kDa	0	0	4	11	2	2	5	7	2.8	0.001
Alpha-enolase OS=Mus musculus GN=Eno1 PE=1 SV=3	P17182	47 kDa	0	0	6	7	2	2	7	4	2.8	0.0026
Aldehyde dehydrogenase, mitochondrial OS=Mus musculus GN=Aldh2 PE=1 SV=1	P47738	57 kDa	0	0	5	9	2	2	6	5	2.8	0.0016
U3 small nucleolar RNA-associated protein 6 homolog OS=Mus musculus GN=Utp6 PE=2 SV=1	Q8VCY6	70 kDa	0	0	4	11	2	2	5	7	2.8	0.001
Something about silencing protein 10 OS=Mus musculus GN=Utp3 PE=1 SV=1	Q9JI13	53 kDa	0	0	4	11	2	2	5	7	2.8	0.001
High mobility group protein HMG-I/HMG-Y OS=Mus musculus GN=Hmga1 PE=1 SV=4	P17095	12 kDa	0	0	6	7	2	2	7	4	2.8	0.0026
Replication factor C subunit 5 OS=Mus musculus GN=Rfc5 PE=2 SV=1	Q9D0F6	38 kDa	0	0	6	7	2	2	7	4	2.8	0.0026
2-oxoglutarate dehydrogenase, mitochondrial OS=Mus musculus GN=Ogdh PE=1 SV=3	Q60597 (+1)	116 kDa	0	0	5	9	2	2	6	5	2.8	0.0016
Heterogeneous nuclear ribonucleoprotein D0 OS=Mus musculus GN=Hnrnpd PE=1 SV=2	Q60668	38 kDa	0	0	0	15	2	2	2	9	2.8	0.001
L1TD1 OS=Mus musculus GN=L1td1 PE=1 SV=1	G3UYN0 (+1)	95 kDa	4	4	13	24	6	5	15	14	2.7	0.0046
Inner centromere protein OS=Mus musculus GN=Incenp PE=1 SV=2	Q9WU62	101 kDa	3	0	9	11	4	2	10	7	2.7	0.012
Myelin expression factor 2 OS=Mus musculus GN=Myef2 PE=1 SV=1	Q8C854	63 kDa	0	3	7	13	2	4	8	8	2.7	0.012
Probable 28S rRNA (cytosine-C(5))-methyltransferase OS=Mus musculus GN=Nop2 PE=1 SV=1	E9QN31	87 kDa	6	8	26	32	9	10	30	19	2.6	0.0011
Interleukin enhancer-binding factor 3 OS=Mus musculus GN=Ilf3 PE=1 SV=2	Q9Z1X4	96 kDa	7	3	19	24	10	4	22	14	2.6	0.0037
Heterogeneous nuclear ribonucleoprotein Q OS=Mus musculus GN=Syncrip PE=1 SV=2	Q7TMK9	70 kDa	0	5	9	20	2	6	10	12	2.6	0.0042
CAD protein OS=Mus musculus GN=Cad PE=1 SV=1	B2RQC6 (+2)	243 kDa	0	0	5	8	2	2	6	5	2.6	0.0026
Collagen alpha-1(XVIII) chain OS=Mus musculus GN=Col18a1 PE=1 SV=1	E9QPX1 (+1)	182 kDa	0	0	6	6	2	2	7	4	2.6	0.004
ATPase family AAA domain-containing protein 5 OS=Mus musculus GN=Atad5 PE=1 SV=1	Q4QY64 (+1)	204 kDa	0	0	7	4	2	2	8	2	2.6	0.0064
UDP-N-acetylglucosamine--peptide N-acetylglucosaminyltransferase 110 kDa subunit OS=Mus musculus GN=Ogt PE=1 SV=2	Q8CGY8	117 kDa	0	0	6	6	2	2	7	4	2.6	0.004
Exosome complex component RRP43 OS=Mus musculus GN=Exosc8 PE=2 SV=1	Q9D753	30 kDa	0	0	5	8	2	2	6	5	2.6	0.0026
pre-rRNA processing protein FTSJ3 OS=Mus musculus GN=Ftsj3 PE=1 SV=1	Q9DBE9	96 kDa	4	6	13	32	6	8	15	19	2.5	0.0022
Leucine-rich repeat and WD repeat-containing protein 1 OS=Mus musculus GN=LRWD1 PE=2 SV=1	Q8BUI3	72 kDa	3	0	5	16	4	2	6	10	2.5	0.0083
Mediator of DNA damage checkpoint protein 1 OS=Mus musculus GN=Mdc1 PE=1 SV=1	E9QK89 (+1)	185 kDa	0	0	5	7	2	2	6	4	2.5	0.004
Putative ATP-dependent RNA helicase DHX33 OS=Mus musculus GN=Dhx33 PE=1 SV=1	Q80VY9	78 kDa	1	0	4	9	2	2	5	5	2.5	0.015
RNA binding motif protein 34 OS=Mus musculus GN=Rbm34 PE=1 SV=1	B2RUP0 (+1)	49 kDa	0	0	2	13	2	2	2	8	2.5	0.001
Plectin OS=Mus musculus GN=Plec PE=1 SV=1	E9Q3W4 (+1)	499 kDa	0	0	4	9	2	2	5	5	2.5	0.0026
Nucleolar protein 8 OS=Mus musculus GN=Nol8 PE=1 SV=1	E9QKD1 (+1)	131 kDa	0	0	3	11	2	2	3	7	2.5	0.0016
Replication factor C subunit 3 OS=Mus musculus GN=Rfc3 PE=1 SV=1	Q8R323	41 kDa	0	0	3	11	2	2	3	7	2.5	0.0016
Leucine-rich repeat-containing protein 59 OS=Mus musculus GN=Lrrc59 PE=1 SV=1	Q922Q8	35 kDa	0	0	4	9	2	2	5	5	2.5	0.0026
Proline-, glutamic acid- and leucine-rich protein 1 OS=Mus musculus GN=Pelp1 PE=1 SV=2	Q9DBD5	118 kDa	0	0	5	7	2	2	6	4	2.5	0.004
Methyl-CpG-binding domain protein 3 OS=Mus musculus GN=Mbd3 PE=1 SV=1	D3YTR4 (+2)	31 kDa	0	0	4	9	2	2	5	5	2.5	0.0026
Histone-lysine N-methyltransferase 2B OS=Mus musculus GN=Kmt2b PE=1 SV=1	F8WJ40 (+1)	296 kDa	0	0	3	11	2	2	3	7	2.5	0.0016
Exosome complex component RRP4 OS=Mus musculus GN=Exosc2 PE=2 SV=1	Q8VBV3	33 kDa	0	0	5	7	2	2	6	4	2.5	0.004
14-3-3 protein zeta/delta OS=Mus musculus GN=Ywhaz PE=1 SV=1	P63101	28 kDa	0	0	6	5	2	2	7	3	2.5	0.0064
Lamin-B2 OS=Mus musculus GN=Lmnb2 PE=1 SV=2	P21619	67 kDa	0	0	6	5	2	2	7	3	2.5	0.0064
DNA topoisomerase 2-alpha OS=Mus musculus GN=Top2a PE=1 SV=2	Q01320	173 kDa	22	9	43	89	31	12	49	54	2.4	< 0.00010
Histone H2A OS=Mus musculus GN=H2afj PE=1 SV=1	A0A0N4SV66 (+1)	14 kDa	8	0	26	0	11	2	30	2	2.4	0.073
Probable ATP-dependent RNA helicase DDX10 OS=Mus musculus GN=Ddx10 PE=1 SV=2	Q80Y44	101 kDa	0	4	6	17	2	5	7	10	2.4	0.011
60S ribosomal protein L13 OS=Mus musculus GN=Rpl13 PE=1 SV=3	P47963	24 kDa	1	3	6	12	2	4	7	7	2.4	0.051
Fragile X mental retardation syndrome-related protein 1 OS=Mus musculus GN=Fxr1 PE=1 SV=2	Q61584	76 kDa	0	4	10	10	2	5	11	6	2.4	0.028
Ubiquitin carboxyl-terminal hydrolase 36 OS=Mus musculus GN=Usp36 PE=1 SV=1	B1AQJ2	120 kDa	0	1	3	10	2	2	3	6	2.4	0.015
KRR1 small subunit processome component homolog OS=Mus musculus GN=Krr1 PE=2 SV=1	Q8BGA5	44 kDa	2	0	5	10	3	2	6	6	2.4	0.023
Serine/arginine repetitive matrix protein 1 OS=Mus musculus GN=Srrm1 PE=1 SV=1	A2A8V8 (+4)	103 kDa	0	2	5	9	2	3	6	5	2.4	0.033
U3 small nucleolar RNA-associated protein 15 homolog OS=Mus musculus GN=Utp15 PE=1 SV=1	Q8C7V3	59 kDa	0	0	3	10	2	2	3	6	2.4	0.0026
60S ribosomal protein L14 OS=Mus musculus GN=Rpl14 PE=1 SV=3	Q9CR57	24 kDa	0	0	4	8	2	2	5	5	2.4	0.004
Structural maintenance of chromosomes flexible hinge domain-containing protein 1 OS=Mus musculus GN=Smchd1 PE=1 SV=2	Q6P5D8	226 kDa	0	0	4	8	2	2	5	5	2.4	0.004
H/ACA ribonucleoprotein complex subunit 2 OS=Mus musculus GN=Nhp2 PE=1 SV=1	Q9CRB2	17 kDa	0	0	4	8	2	2	5	5	2.4	0.004
60S acidic ribosomal protein P2 OS=Mus musculus GN=Rplp2 PE=1 SV=3	P99027	12 kDa	8	4	19	26	11	5	22	16	2.3	0.008
Cbx3 protein OS=Mus musculus GN=Cbx3 PE=1 SV=1	Q9DCC5	21 kDa	8	3	19	21	11	4	22	13	2.3	0.015
Protein AATF OS=Mus musculus GN=Aatf PE=1 SV=1	Q9JKX4	59 kDa	4	3	11	15	6	4	13	9	2.3	0.043
Protein Rsf1 OS=Mus musculus GN=Rsf1 PE=1 SV=2	E9PWW9	161 kDa	4	0	5	20	6	2	6	12	2.3	0.0059
40S ribosomal protein SA OS=Mus musculus GN=Rpsa PE=1 SV=4	P14206	33 kDa	0	4	6	16	2	5	7	10	2.3	0.015
Peptidyl-prolyl cis-trans isomerase B OS=Mus musculus GN=Ppib PE=1 SV=2	P24369	24 kDa	0	3	6	11	2	4	7	7	2.3	0.031
Transcription activator BRG1 OS=Mus musculus GN=Smarca4 PE=1 SV=1	Q3TKT4	181 kDa	0	1	5	6	2	2	6	4	2.3	0.032
E3 ubiquitin-protein ligase TRIM71 OS=Mus musculus GN=Trim71 PE=1 SV=1	Q1PSW8	92 kDa	0	1	0	12	2	2	2	7	2.3	0.022
G patch domain-containing protein 4 OS=Mus musculus GN=Gpatch4 PE=1 SV=1	Q3TFK5	47 kDa	0	0	5	6	2	2	6	4	2.3	0.0064
Lamina-associated polypeptide 2, isoforms alpha/zeta OS=Mus musculus GN=Tmpo PE=1 SV=4	Q61033	75 kDa	0	0	0	12	2	2	2	7	2.3	0.004
Stress-70 protein, mitochondrial OS=Mus musculus GN=Hspa9 PE=1 SV=3	P38647	73 kDa	8	5	22	23	11	6	25	14	2.2	0.014
WD repeat-containing protein 43 OS=Mus musculus GN=Wdr43 PE=2 SV=2	Q6ZQL4	75 kDa	6	0	9	21	9	2	10	13	2.2	0.0073
Periodic tryptophan protein 2 homolog OS=Mus musculus GN=Pwp2 PE=1 SV=1	Q8BU03	103 kDa	3	0	5	13	4	2	6	8	2.2	0.023
Insulin-like growth factor 2 mRNA-binding protein 3 OS=Mus musculus GN=Igf2bp3 PE=1 SV=1	Q9CPN8	64 kDa	4	2	10	11	6	3	11	7	2.2	0.081
S1 RNA-binding domain-containing protein 1 OS=Mus musculus GN=Srbd1 PE=1 SV=1	F8WGW3 (+1)	110 kDa	0	0	4	7	2	2	5	4	2.2	0.0064
DNA-directed RNA polymerase I subunit RPA1 OS=Mus musculus GN=Polr1a PE=1 SV=2	O35134	194 kDa	0	0	3	9	2	2	3	5	2.2	0.004
Transcription factor 20 OS=Mus musculus GN=Tcf20 PE=1 SV=3	Q9EPQ8	216 kDa	0	0	3	9	2	2	3	5	2.2	0.004
Protein Atad2b OS=Mus musculus GN=Atad2b PE=1 SV=1	E9Q166	165 kDa	0	0	3	9	2	2	3	5	2.2	0.004
Heterochromatin protein 1-binding protein 3 OS=Mus musculus GN=Hp1bp3 PE=1 SV=1	Q3TEA8 (+1)	61 kDa	0	0	4	7	2	2	5	4	2.2	0.0064
Borealin OS=Mus musculus GN=Cdca8 PE=1 SV=2	Q8BHX3	32 kDa	0	0	3	9	2	2	3	5	2.2	0.004
60S ribosomal protein L13a OS=Mus musculus GN=Rpl13a PE=1 SV=4	P19253	23 kDa	0	0	0	11	2	2	2	7	2.2	0.0064
Zinc finger protein 518B OS=Mus musculus GN=Znf518b PE=2 SV=1	B2RRE4 (+1)	119 kDa	0	0	6	3	2	2	7	2	2.2	0.016
Exosome complex component MTR3 OS=Mus musculus GN=Exosc6 PE=1 SV=1	Q8BTW3	28 kDa	0	0	5	5	2	2	6	3	2.2	0.01
Armadillo repeat-containing protein 10 OS=Mus musculus GN=Armc10 PE=1 SV=1	Q9D0L7	33 kDa	0	0	4	7	2	2	5	4	2.2	0.0064
Ribosomal biogenesis protein LAS1L OS=Mus musculus GN=Las1l PE=1 SV=1	A2BE28	89 kDa	0	0	0	11	2	2	2	7	2.2	0.0064
Protein polybromo-1 (Fragment) OS=Mus musculus GN=Pbrm1 PE=1 SV=7	D3YYF2 (+7)	168 kDa	0	0	6	1	2	2	7	2	2.2	0.04
Y-box-binding protein 3 OS=Mus musculus GN=Ybx3 PE=1 SV=2	Q9JKB3	39 kDa	0	0	5	5	2	2	6	3	2.2	0.01

Chromatin target of PRMT1 protein OS=Mus musculus GN=Chtop PE=1 SV=1	E9PW20 (+1)	22 kDa	0	0	6	0	2	2	7	2	2.2	0.064
Pyruvate kinase PKM OS=Mus musculus GN=Pkm PE=1 SV=4	P52480	58 kDa	0	0	6	0	2	2	7	2	2.2	0.064
RNA polymerase II-associated factor 1 homolog OS=Mus musculus GN=Paf1 PE=1 SV=1	Q8K2T8	61 kDa	0	0	6	0	2	2	7	2	2.2	0.064
Histone-lysine N-methyltransferase OS=Mus musculus GN=Nsd1 PE=1 SV=1	E9QAE4	296 kDa	3	3	9	11	4	4	10	7	2.1	0.1
FACT complex subunit SSRP1 OS=Mus musculus GN=Ssrp1 PE=1 SV=2	Q08943	81 kDa	4	0	5	17	6	2	6	10	2.1	0.015
60S acidic ribosomal protein P0 OS=Mus musculus GN=Gm8730 PE=1 SV=1	E9Q070	34 kDa	7	0	20	0	10	2	23	2	2.1	0.17
Superkiller viralicidic activity 2-like 2 OS=Mus musculus GN=Skiv2l2 PE=1 SV=1	Q9CZU3	118 kDa	2	2	7	6	3	3	8	4	2.1	0.19
Protein Urb2 OS=Mus musculus GN=Urb2 PE=1 SV=1	E9Q7L1	172 kDa	0	0	2	10	2	2	2	6	2.1	0.004
AT-hook DNA-binding motif-containing protein 1 OS=Mus musculus GN=Ahdc1 PE=1 SV=1	Q6PAL7	168 kDa	0	0	3	8	2	2	3	5	2.1	0.0064
Probable dimethyladenosine transferase OS=Mus musculus GN=Dimt1 PE=2 SV=1	Q9D0D4	35 kDa	0	0	2	10	2	2	2	6	2.1	0.004
Barrier-to-autointegration factor OS=Mus musculus GN=Banf1 PE=1 SV=1	O54962	10 kDa	0	0	4	6	2	2	5	4	2.1	0.01
RIKEN cDNA 5830416A07, isoform CRA_c OS=Mus musculus GN=Zc3h18 PE=1 SV=1	G3X8T2 (+1)	108 kDa	0	0	4	6	2	2	5	4	2.1	0.01
Vimentin OS=Mus musculus GN=Vim PE=1 SV=1	A0A0A6YWC8 (+1)	49 kDa	0	0	4	6	2	2	5	4	2.1	0.01
Zinc finger protein 638 OS=Mus musculus GN=Zfml PE=4 SV=1	E9QML5	214 kDa	0	0	3	8	2	2	3	5	2.1	0.0064
MCG1407, isoform CRA_a OS=Mus musculus GN=Rpf2 PE=1 SV=1	G3X926	31 kDa	0	0	4	6	2	2	5	4	2.1	0.01
60S ribosomal protein L26 OS=Mus musculus GN=Rpl26 PE=1 SV=1	P61255	17 kDa	0	0	4	6	2	2	5	4	2.1	0.01
DNA replication licensing factor MCM2 OS=Mus musculus GN=Mcm2 PE=1 SV=3	P97310	102 kDa	0	0	4	6	2	2	5	4	2.1	0.01
Histone acetyltransferase KAT6B OS=Mus musculus GN=Kat6b PE=1 SV=3	Q8BRB7	209 kDa	0	0	3	8	2	2	3	5	2.1	0.0064
Ribosomal RNA-processing protein 7 homolog A OS=Mus musculus GN=Rrp7a PE=2 SV=1	Q9D1C9	32 kDa	0	0	4	6	2	2	5	4	2.1	0.01
Metastasis-associated protein MTA1 OS=Mus musculus GN=Mta1 PE=1 SV=1	E9PX23 (+2)	79 kDa	0	0	4	6	2	2	5	4	2.1	0.01
60S acidic ribosomal protein P0 OS=Mus musculus GN=Rplp0 PE=1 SV=3	P14869	34 kDa	0	6	0	30	2	8	2	18	2.1	0.0073
Small subunit processome component 20 homolog OS=Mus musculus GN=Utp20 PE=1 SV=1	E9QK83	318 kDa	12	6	23	38	17	8	26	23	2	0.0054
Protein Jumonji OS=Mus musculus GN=Jarid2 PE=1 SV=1	Q62315	137 kDa	2	8	10	25	3	10	11	15	2	0.027
60S ribosomal protein L18 OS=Mus musculus GN=Rpl18 PE=1 SV=3	P35980	22 kDa	2	3	5	13	3	4	6	8	2	0.097
Heterogeneous nuclear ribonucleoprotein U-like protein 2 OS=Mus musculus GN=Hnrnpul2 PE=1 SV=2	Q00PI9	85 kDa	3	2	6	11	4	3	7	7	2	0.12
60S ribosomal protein L24 OS=Mus musculus GN=Rpl24 PE=1 SV=2	Q8BP67	18 kDa	3	2	8	8	4	3	9	5	2	0.16
RNA-binding protein FUS (Fragment) OS=Mus musculus GN=Fus PE=1 SV=1	G3UXT7 (+3)	14 kDa	3	0	6	9	4	2	7	5	2	0.058
ATPase family AAA domain-containing protein 2 OS=Mus musculus GN=Atad2 PE=1 SV=1	G3X963	155 kDa	2	0	2	12	3	2	2	7	2	0.033
Lysine-rich nucleolar protein 1 OS=Mus musculus GN=Knop1 PE=1 SV=1	E9QKQ3 (+1)	60 kDa	0	2	5	6	2	3	6	4	2	0.09
Eukaryotic translation initiation factor 2 subunit 3, X-linked OS=Mus musculus GN=Eif2s3x PE=1 SV=2	Q9Z0N1	51 kDa	0	0	0	10	2	2	2	6	2	0.01
Dehydrogenase/reductase SDR family member 4 OS=Mus musculus GN=Dhrs4 PE=1 SV=3	Q99LB2	30 kDa	0	0	5	4	2	2	6	2	2	0.016
60S acidic ribosomal protein P1 OS=Mus musculus GN=Rplp1 PE=1 SV=1	P47955	11 kDa	0	0	0	10	2	2	2	6	2	0.01
Heat shock protein beta-1 OS=Mus musculus GN=Hspb1 PE=1 SV=3	P14602	23 kDa	0	0	5	4	2	2	6	2	2	0.016
Bromodomain and WD repeat-containing protein 1 (Fragment) OS=Mus musculus GN=Brwd1 PE=1 SV=1	E9Q2N1 (+1)	295 kDa	0	0	5	4	2	2	6	2	2	0.016
Histone-binding protein RBBP7 OS=Mus musculus GN=Rbbp7 PE=1 SV=1	A2AFJ1 (+1)	47 kDa	0	0	0	10	2	2	2	6	2	0.01
Nucleolar protein 56 OS=Mus musculus GN=Nop56 PE=1 SV=2	Q9D6Z1	64 kDa	14	14	30	61	20	18	34	37	1.9	0.0013
Transcription intermediary factor 1-beta OS=Mus musculus GN=Trim28 PE=1 SV=3	Q62318	89 kDa	13	13	38	41	18	17	44	25	1.9	0.0058
Protein Wdr36 OS=Mus musculus GN=Wdr36 PE=1 SV=1	Q3TAQ9	100 kDa	11	5	17	37	16	6	20	22	1.9	0.009
Heterogeneous nuclear ribonucleoproteins A2/B1 OS=Mus musculus GN=Hnrnpa2b1 PE=1 SV=2	O88569	37 kDa	0	15	19	32	2	19	22	19	1.9	0.01
FACT complex subunit SPT16 OS=Mus musculus GN=Supt16 PE=1 SV=1	G3X956 (+1)	120 kDa	7	4	11	28	10	5	13	17	1.9	0.019
60S ribosomal protein L7 OS=Mus musculus GN=Rpl7 PE=1 SV=2	P14148	31 kDa	5	0	7	16	7	2	8	10	1.9	0.025
Death-inducer obliterator 1 OS=Mus musculus GN=Dido1 PE=1 SV=4	Q8C9B9	247 kDa	0	6	9	13	2	8	10	8	1.9	0.064
Elongation factor 2 OS=Mus musculus GN=Eef2 PE=1 SV=2	P58252	95 kDa	0	4	4	15	2	5	5	9	1.9	0.038
60S ribosomal protein L5 OS=Mus musculus GN=Rpl5 PE=1 SV=3	P47962	34 kDa	0	3	5	9	2	4	6	5	1.9	0.078
Kinectin OS=Mus musculus GN=Ktn1 PE=1 SV=1	A0A087WP14 (+9)	147 kDa	0	0	4	5	2	2	5	3	1.9	0.016
Serine/arginine-rich splicing factor 9 OS=Mus musculus GN=Srsf9 PE=1 SV=1	Q9D0B0	26 kDa	0	2	3	9	2	3	3	5	1.9	0.065
AP-2 complex subunit beta OS=Mus musculus GN=Ap2b1 PE=1 SV=1	H3BKM0 (+1)	101 kDa	0	0	1	9	2	2	2	5	1.9	0.01
60S ribosomal protein L35a OS=Mus musculus GN=Rpl35a PE=1 SV=2	O55142	13 kDa	0	0	4	5	2	2	5	3	1.9	0.016
Bifunctional polynucleotide phosphatase/kinase OS=Mus musculus GN=Pnkp PE=1 SV=1	E9Q9A5 (+2)	53 kDa	0	0	2	9	2	2	2	5	1.9	0.0064
mRNA turnover protein 4 homolog OS=Mus musculus GN=Mrto4 PE=1 SV=1	Q9D0I8	28 kDa	0	0	2	9	2	2	2	5	1.9	0.0064
Nuclear pore complex protein Nup133 OS=Mus musculus GN=Nup133 PE=1 SV=2	Q8R0G9	129 kDa	0	0	1	9	2	2	2	5	1.9	0.01
Replication factor C subunit 2 OS=Mus musculus GN=Rfc2 PE=2 SV=1	Q9WUK4	39 kDa	0	0	3	7	2	2	3	4	1.9	0.01
Nucleolar complex protein 4 homolog OS=Mus musculus GN=Noc4l PE=2 SV=1	Q8BHY2	59 kDa	0	0	0	9	2	2	2	5	1.9	0.016
La-related protein 7 OS=Mus musculus GN=Larp7 PE=1 SV=2	Q05CL8	65 kDa	0	0	4	5	2	2	5	3	1.9	0.016
Centromere protein V OS=Mus musculus GN=Cenpv PE=1 SV=2	Q9CXS4	28 kDa	0	0	3	7	2	2	3	4	1.9	0.01
Bifunctional lysine-specific demethylase and histidyl-hydroxylase NO66 OS=Mus musculus GN=No66 PE=1 SV=2	Q9JJF3	68 kDa	0	0	0	9	2	2	2	5	1.9	0.016
Histone-lysine N-methyltransferase EZH2 OS=Mus musculus GN=Ezh2 PE=1 SV=1	D3Z774 (+2)	81 kDa	0	0	4	5	2	2	5	3	1.9	0.016
Neuroguidin OS=Mus musculus GN=Ngdn PE=1 SV=1	Q9DB96	36 kDa	0	0	3	7	2	2	3	4	1.9	0.01
DNA ligase 1 OS=Mus musculus GN=Lig1 PE=1 SV=2	P37913 (+1)	102 kDa	0	0	0	9	2	2	2	5	1.9	0.016
Protein MAK16 homolog OS=Mus musculus GN=Mak16 PE=1 SV=1	Q8BGS0	35 kDa	0	0	0	9	2	2	2	5	1.9	0.016
Calcium-transporting ATPase OS=Mus musculus GN=Atp2a2 PE=1 SV=2	J3KMM5 (+1)	110 kDa	0	0	4	5	2	2	5	3	1.9	0.016
Transformer-2 protein homolog alpha OS=Mus musculus GN=Tra2a PE=1 SV=1	E9QP00 (+1)	33 kDa	0	0	3	7	2	2	3	4	1.9	0.01
Histone deacetylase 2 OS=Mus musculus GN=Hdac2 PE=1 SV=1	P70288	55 kDa	0	0	0	9	2	2	2	5	1.9	0.016
N-acetyltransferase ESCO2 OS=Mus musculus GN=Esco2 PE=2 SV=3	Q8CIB9	67 kDa	0	0	5	0	2	2	6	2	1.9	0.1
C-1-tetrahydrofolate synthase, cytoplasmic OS=Mus musculus GN=Mthfd1 PE=1 SV=4	Q922D8	101 kDa	0	0	5	0	2	2	6	2	1.9	0.1
14-3-3 protein gamma OS=Mus musculus GN=Ywhag PE=1 SV=2	P61982 (+1)	28 kDa	0	0	5	0	2	2	6	2	1.9	0.1
Tubulin beta-5 chain OS=Mus musculus GN=Tubb5 PE=1 SV=1	P99024	50 kDa	11	5	20	27	16	6	23	16	1.8	0.037
Chromodomain-helicase-DNA-binding protein 4 OS=Mus musculus GN=Chd4 PE=1 SV=1	E9QAS4 (+2)	216 kDa	5	6	10	25	7	8	11	15	1.8	0.045
Pre-mRNA-processing factor 40 homolog A OS=Mus musculus GN=Prpf40a PE=1 SV=1	Q9R1C7	108 kDa	1	0	3	6	2	2	3	4	1.8	0.069
Serrate RNA effector molecule homolog OS=Mus musculus GN=Srrt PE=1 SV=1	Q99MR6	100 kDa	2	2	7	3	3	3	8	2	1.8	0.37
Paired amphipathic helix protein Sin3a OS=Mus musculus GN=Sin3a PE=1 SV=3	Q60520	145 kDa	0	0	2	8	2	2	2	5	1.8	0.01
ATP-binding cassette sub-family F member 2 OS=Mus musculus GN=Abcf2 PE=1 SV=1	Q99LE6	72 kDa	0	0	3	6	2	2	3	4	1.8	0.016
Leucine-rich PPR motif-containing protein, mitochondrial OS=Mus musculus GN=Lrpprc PE=1 SV=2	Q6PB66	157 kDa	0	0	4	4	2	2	5	2	1.8	0.025
Catenin alpha-1 OS=Mus musculus GN=Ctnna1 PE=1 SV=1	P26231	100 kDa	0	0	3	6	2	2	3	4	1.8	0.016
Cell division cycle-associated protein 2 OS=Mus musculus GN=Cdca2 PE=1 SV=2	Q14B71	106 kDa	0	0	3	6	2	2	3	4	1.8	0.016
Exosome complex component RRP45 OS=Mus musculus GN=Exosc9 PE=1 SV=1	Q9JHI7	49 kDa	0	0	3	6	2	2	3	4	1.8	0.016
Uncharacterized protein C12orf43 homolog OS=Mus musculus PE=2 SV=1	Q3UY34	28 kDa	0	0	4	4	2	2	5	2	1.8	0.025
Protein TASOR OS=Mus musculus GN=Fam208a PE=1 SV=2	Q69ZR9	181 kDa	0	0	3	6	2	2	3	4	1.8	0.016
Guanine nucleotide-binding protein-like 3-like protein OS=Mus musculus GN=Gnl3l PE=1 SV=1	Q6PGG6	65 kDa	0	0	3	6	2	2	3	4	1.8	0.016
Notchless protein homolog 1 OS=Mus musculus GN=Nle1 PE=1 SV=4	Q8VEJ4	53 kDa	0	0	3	6	2	2	3	4	1.8	0.016
Msx2-interacting protein OS=Mus musculus GN=Spen PE=1 SV=1	A2ADB0 (+1)	399 kDa	0	0	4	4	2	2	5	2	1.8	0.025
WD repeat-containing protein 18 OS=Mus musculus GN=Wdr18 PE=1 SV=1	Q4VBE8	47 kDa	0	0	4	4	2	2	5	2	1.8	0.025
DNA-directed RNA polymerase II subunit RPB4 OS=Mus musculus GN=Polr2d PE=1 SV=2	Q9D7M8	16 kDa	0	0	4	4	2	2	5	2	1.8	0.025
AP-2 complex subunit alpha-1 OS=Mus musculus GN=Ap2a1 PE=1 SV=1	P17426	108 kDa	0	0	3	6	2	2	3	4	1.8	0.016
Serine/threonine-protein phosphatase 2A 65 kDa regulatory subunit A alpha isoform OS=Mus musculus GN=Ppp2r1a PE=1 SV=3	Q76MZ3	65 kDa	0	0	3	6	2	2	3	4	1.8	0.016
Myb-binding protein 1A OS=Mus musculus GN=Mybbp1a PE=1 SV=2	Q7TPV4	152 kDa	42	29	82	121	60	38	94	73	1.7	< 0.00010
Heterogeneous nuclear ribonucleoproteins C1/C2 OS=Mus musculus GN=Hnrnpc PE=1 SV=1	Q9Z204	34 kDa	11	10	25	31	16	13	29	19	1.7	0.05
Telomere-associated protein RIF1 OS=Mus musculus GN=Rif1 PE=1 SV=2	Q6PR54	266 kDa	6	11	12	42	9	14	14	25	1.7	0.015
Histone H1.5 OS=Mus musculus GN=Hist1h1b PE=1 SV=2	P43276	23 kDa	6	7	16	19	9	9	18	11	1.7	0.1
Undifferentiated embryonic cell transcription factor 1 OS=Mus musculus GN=Utf1 PE=1 SV=2	Q6J1H4	36 kDa	7	0	8	19	10	2	9	11	1.7	0.033
40S ribosomal protein S17 OS=Mus musculus GN=Rps17 PE=1 SV=2	P63276	16 kDa	3	5	6	19	4	6	7	11	1.7	0.091
Eukaryotic translation initiation factor 6 OS=Mus musculus GN=Eif6 PE=1 SV=2	O55135 (+1)	27 kDa	4	0	8	7	6	2	9	4	1.7	0.12
MCG16685, isoform CRA_d OS=Mus musculus GN=Wtap PE=1 SV=1	E0CYH0	44 kDa	0	4	8	5	2	5	9	3	1.7	0.19
Nucleolar complex protein 2 homolog OS=Mus musculus GN=Noc2l PE=1 SV=2	Q9WV70	85 kDa	3	0	5	8	4	2	6	5	1.7	0.1
Bromodomain adjacent to zinc finger domain protein 1A OS=Mus musculus GN=Baz1a PE=1 SV=1	G3UWZ0 (+1)	178 kDa	0	0	0	8	2	2	2	5	1.7	0.025
ATP-dependent RNA helicase DDX50 OS=Mus musculus GN=Ddx50 PE=2 SV=1	Q99MJ9	82 kDa	0	0	0	8	2	2	2	5	1.7	0.025
Surfeit locus protein 6 OS=Mus musculus GN=Surf6 PE=1 SV=1	P70279	41 kDa	0	0	0	8	2	2	2	5	1.7	0.025
Nuclear receptor subfamily 0 group B member 1 OS=Mus musculus GN=Nr0b1 PE=1 SV=1	Q61066	53 kDa	0	0	0	8	2	2	2	5	1.7	0.025
Histone H2B type 1-F/J/L OS=Mus musculus GN=Hist1h2bf PE=1 SV=2	P10853 (+8)	14 kDa	35	13	43	97	50	17	49	59	1.6	0.00063
Nucleolar RNA helicase 2 OS=Mus musculus GN=Ddx21 PE=1 SV=3	Q9JIK5	94 kDa	20	30	42	96	28	39	48	58	1.6	0.0019
Histone H4 OS=Mus musculus GN=Hist2h4 PE=1 SV=1	B2RTM0 (+1)	11 kDa	27	14	22	111	38	18	25	67	1.6	0.00012
Heterogeneous nuclear ribonucleoprotein F OS=Mus musculus GN=Hnrnpf PE=1 SV=3	Q9Z2X1	46 kDa	7	12	19	30	10	16	22	18	1.6	0.08
Matrin-3 OS=Mus musculus GN=Matr3 PE=1 SV=1	Q8K310	95 kDa	8	6	10	31	11	8	11	19	1.6	0.051

60S ribosomal protein L6 OS=Mus musculus GN=Rpl6 PE=1 SV=3	P47911	34 kDa	5	4	9	16	7	5	10	10	1.6	0.14
Putative oxidoreductase GLYR1 OS=Mus musculus GN=Glyr1 PE=1 SV=1	D3YYT1	60 kDa	7	0	9	14	10	2	10	8	1.6	0.087
Protein Mdn1 OS=Mus musculus GN=Mdn1 PE=1 SV=1	A2ANY6 (+1)	630 kDa	3	0	3	11	4	2	3	7	1.6	0.078
Kinesin-like protein KIF23 OS=Mus musculus GN=Kif23 PE=1 SV=1	E9Q5G3	109 kDa	0	3	2	12	2	4	2	7	1.6	0.078
LINE-1 retrotransposable element ORF1 protein OS=Mus musculus PE=1 SV=2	P11260	41 kDa	0	3	3	10	2	4	3	6	1.6	0.1
Polycomb protein Suz12 OS=Mus musculus GN=Suz12 PE=1 SV=1	E9PW15 (+1)	80 kDa	0	4	6	8	2	5	7	5	1.6	0.15
Heterogeneous nuclear ribonucleoprotein A0 OS=Mus musculus GN=Hnrnpa0 PE=1 SV=1	Q9CX86	31 kDa	0	4	5	9	2	5	6	5	1.6	0.15
Serine/arginine-rich splicing factor 6 OS=Mus musculus GN=Srsf6 PE=1 SV=1	Q3TWW8	39 kDa	1	0	4	3	2	2	5	2	1.6	0.14
Metastasis-associated protein MTA3 OS=Mus musculus GN=Mta3 PE=1 SV=1	E9Q794 (+3)	58 kDa	0	2	5	9	2	5	6	5	1.6	0.15
H/ACA ribonucleoprotein complex subunit 1 (Fragment) OS=Mus musculus GN=Gar1 PE=1 SV=1	D3YZ09 (+1)	20 kDa	1	0	0	7	2	2	2	4	1.6	0.14
U3 small nucleolar ribonucleoprotein protein IMP4 OS=Mus musculus GN=Imp4 PE=2 SV=1	Q8VHZ7	34 kDa	0	0	3	5	2	2	3	3	1.6	0.025
Trifunctional enzyme subunit alpha, mitochondrial OS=Mus musculus GN=Hadha PE=1 SV=1	Q8BMS1	83 kDa	2	0	4	5	3	2	5	3	1.6	0.17
Importin subunit alpha-5 OS=Mus musculus GN=Kpna1 PE=1 SV=2	Q60960	60 kDa	1	0	1	7	2	2	2	4	1.6	0.1
60S ribosomal protein L18a OS=Mus musculus GN=Rpl18a PE=1 SV=1	P62717	21 kDa	0	0	4	3	2	2	5	2	1.6	0.04
Protein Zfp219 OS=Mus musculus GN=Zfp219 PE=1 SV=1	Q6IQX8	78 kDa	0	0	3	5	2	2	3	3	1.6	0.025
DNA-directed RNA polymerase I subunit RPA49 (Fragment) OS=Mus musculus GN=Polr1e PE=1 SV=1	G8JL51 (+1)	46 kDa	0	0	3	5	2	2	3	3	1.6	0.025
T-complex protein 1 subunit zeta OS=Mus musculus GN=Cct6a PE=1 SV=3	P80317	58 kDa	0	0	3	5	2	2	3	3	1.6	0.025
M-phase phosphoprotein 8 OS=Mus musculus GN=Mphosph8 PE=1 SV=1	Q3TYA6	97 kDa	0	0	0	7	2	2	2	4	1.6	0.04
60S ribosomal protein L28 OS=Mus musculus GN=Rpl28 PE=1 SV=2	P41105	16 kDa	0	0	3	5	2	2	3	3	1.6	0.025
Eukaryotic translation initiation factor 2 subunit 2 OS=Mus musculus GN=Eif2s2 PE=1 SV=1	Q99L45	38 kDa	0	0	3	5	2	2	3	3	1.6	0.025
Zinc finger protein 512 OS=Mus musculus GN=Znf512 PE=2 SV=2	Q69Z99	64 kDa	0	0	4	3	2	2	5	2	1.6	0.04
DnaJ homolog subfamily C member 9 OS=Mus musculus GN=Dnajc9 PE=1 SV=2	Q91WN1	30 kDa	0	0	4	3	2	2	5	2	1.6	0.04
Spectrin alpha chain, non-erythrocytic 1 OS=Mus musculus GN=Sptan1 PE=1 SV=1	A3KGU5 (+3)	283 kDa	0	0	4	3	2	2	5	2	1.6	0.04
Pre-mRNA-splicing factor CWC22 homolog OS=Mus musculus GN=Cwc22 PE=4 SV=1	B1AYU7 (+1)	104 kDa	0	0	3	5	2	2	3	3	1.6	0.025
WD repeat-containing protein 74 OS=Mus musculus GN=Wdr74 PE=2 SV=1	Q8VCG3	43 kDa	0	0	3	5	2	2	3	3	1.6	0.025
Nuclear receptor coactivator 5 OS=Mus musculus GN=Ncoa5 PE=1 SV=1	Q91W39	65 kDa	0	0	4	3	2	2	5	2	1.6	0.04
Serine/threonine-protein phosphatase 1 regulatory subunit 10 OS=Mus musculus GN=Ppp1r10 PE=1 SV=1	Q80W00	94 kDa	0	0	4	3	2	2	5	2	1.6	0.04
Heat shock protein 105 kDa OS=Mus musculus GN=Hsph1 PE=1 SV=1	E9Q0U7 (+1)	92 kDa	0	0	3	5	2	2	3	3	1.6	0.025
Ribosome production factor 1 OS=Mus musculus GN=Rpf1 PE=2 SV=2	Q7TND5	40 kDa	0	0	0	7	2	2	2	4	1.6	0.04
T-complex protein 1 subunit theta OS=Mus musculus GN=Cct8 PE=1 SV=3	P42932	60 kDa	0	0	4	3	2	2	5	2	1.6	0.04
MICOS complex subunit Mic60 OS=Mus musculus GN=Immt PE=1 SV=1	Q8CAQ8	84 kDa	0	0	3	5	2	2	3	3	1.6	0.025
Coiled-coil domain-containing protein 86 OS=Mus musculus GN=Ccdc86 PE=1 SV=2	Q9JJ89	46 kDa	0	0	3	5	2	2	3	3	1.6	0.025
Probable ATP-dependent RNA helicase DDX49 OS=Mus musculus GN=Ddx49 PE=2 SV=1	Q4FZF3	54 kDa	0	0	4	3	2	2	5	2	1.6	0.04
Max protein OS=Mus musculus GN=Max PE=1 SV=1	B2RS19 (+1)	18 kDa	0	0	4	2	2	2	5	2	1.6	0.064
Insulin-like growth factor 2 mRNA-binding protein 2 OS=Mus musculus GN=Igf2bp2 PE=1 SV=1	A6X8Z3 (+1)	58 kDa	0	0	0	7	2	2	2	4	1.6	0.04
Cytoskeleton-associated protein 2 OS=Mus musculus GN=Ckap2 PE=1 SV=1	Q3V1H1	74 kDa	0	0	0	7	2	2	2	4	1.6	0.04
Nipped-B-like protein OS=Mus musculus GN=Nipbl PE=1 SV=1	Q6KCD5	315 kDa	0	0	0	7	2	2	2	4	1.6	0.04
Probable ATP-dependent RNA helicase DDX31 OS=Mus musculus GN=Ddx31 PE=2 SV=2	Q6NZQ2	77 kDa	0	0	0	7	2	2	2	4	1.6	0.04
WD repeat-containing protein 61 OS=Mus musculus GN=Wdr61 PE=2 SV=1	Q9ERF3 (+1)	34 kDa	0	0	4	0	2	2	5	2	1.6	0.16
U3 small nucleolar ribonucleoprotein protein IMP3 OS=Mus musculus GN=Imp3 PE=2 SV=1	Q921Y2	22 kDa	0	0	4	3	2	2	5	2	1.6	0.04
mRNA export factor OS=Mus musculus GN=Rae1 PE=1 SV=1	Q8C570	41 kDa	0	0	4	2	2	2	5	2	1.6	0.064
Nucleolar protein 9 OS=Mus musculus GN=Nop9 PE=1 SV=1	Q8BMC4	70 kDa	0	0	0	7	2	2	2	4	1.6	0.04
DNA-directed RNA polymerase II subunit RPB7 OS=Mus musculus GN=Polr2g PE=1 SV=1	P62488	19 kDa	0	0	4	0	2	2	5	2	1.6	0.16
UPF0568 protein C14orf166 homolog OS=Mus musculus PE=2 SV=1	Q9CQE8 (+1)	28 kDa	0	0	4	0	2	2	5	2	1.6	0.16
60S ribosomal protein L31 OS=Mus musculus GN=Rpl31 PE=1 SV=1	P62900 (+1)	14 kDa	0	0	4	0	2	2	5	2	1.6	0.16
Leucine-rich repeat-containing protein 40 OS=Mus musculus GN=Lrrc40 PE=2 SV=2	Q9CRC8 (+1)	68 kDa	0	0	4	0	2	2	5	2	1.6	0.16
Caspase activity and apoptosis inhibitor 1 OS=Mus musculus GN=Caap1 PE=1 SV=2	Q8VDY9	38 kDa	0	0	4	0	2	2	5	2	1.6	0.16
Zinc finger protein 532 OS=Mus musculus GN=Zfp532 PE=1 SV=1	S4R2I9	141 kDa	0	0	4	0	2	2	5	2	1.6	0.16
Serine/arginine-rich-splicing factor 1 OS=Mus musculus GN=Srsf1 PE=1 SV=1	H7BX95 (+1)	28 kDa	12	10	26	27	17	13	30	16	1.5	0.11
Guanine nucleotide-binding protein subunit beta-2-like 1 OS=Mus musculus GN=Gnb2l1 PE=1 SV=3	P68040	35 kDa	4	8	9	22	6	10	10	13	1.5	0.15
Methylcytosine dioxygenase TET1 OS=Mus musculus GN=Tet1 PE=1 SV=1	E9Q9Y4	223 kDa	4	4	7	14	6	5	8	8	1.5	0.2
Importin subunit beta-1 OS=Mus musculus GN=Kpnb1 PE=1 SV=2	P70168	97 kDa	4	0	5	9	6	2	6	5	1.5	0.15
Rac GTPase-activating protein 1 OS=Mus musculus GN=Racgap1 PE=1 SV=1	Q9WVM1	70 kDa	0	3	4	7	2	4	5	4	1.5	0.18
Cytoplasmic dynein 1 heavy chain 1 OS=Mus musculus GN=Dync1h1 PE=1 SV=2	Q9JHU4	532 kDa	0	0	3	4	2	2	3	2	1.5	0.04
Shugoshin-like 2 OS=Mus musculus GN=Sgol2 PE=1 SV=1	Q7TSY8	130 kDa	0	0	3	4	2	2	3	2	1.5	0.04
T-complex protein 1 subunit alpha OS=Mus musculus GN=Tcp1 PE=1 SV=3	P11983	60 kDa	1	0	3	4	2	2	3	2	1.5	0.14
Ubiquitin-like modifier-activating enzyme 1 OS=Mus musculus GN=Uba1 PE=1 SV=1	Q02053	118 kDa	0	0	3	4	2	2	3	2	1.5	0.04
Host cell factor 1 OS=Mus musculus GN=Hcfc1 PE=1 SV=1	B1AUX2 (+1)	215 kDa	0	0	3	4	2	2	3	2	1.5	0.04
Ras GTPase-activating protein-binding protein 2 OS=Mus musculus GN=G3bp2 PE=1 SV=2	P97379	54 kDa	0	0	3	4	2	2	3	2	1.5	0.04
Ribosomal RNA-processing protein 8 OS=Mus musculus GN=Rrp8 PE=1 SV=1	E9PVA2 (+1)	57 kDa	0	0	3	4	2	2	3	2	1.5	0.04
Pyrroline-5-carboxylate reductase 2 OS=Mus musculus GN=Pycr2 PE=2 SV=1	Q922Q4	34 kDa	0	0	3	4	2	2	3	2	1.5	0.04
Lamin-B1 OS=Mus musculus GN=Lmnb1 PE=1 SV=3	P14733	67 kDa	7	8	15	17	10	10	17	10	1.4	0.3
Transducin beta-like protein 3 OS=Mus musculus GN=Tbl3 PE=2 SV=1	Q8C4J7	88 kDa	5	6	6	22	7	8	7	13	1.4	0.17
tRNA (cytosine(34)-C(5))-methyltransferase OS=Mus musculus GN=Nsun2 PE=1 SV=2	Q1HFZ0	85 kDa	0	10	9	17	2	13	10	10	1.4	0.17
60S ribosomal protein L3 OS=Mus musculus GN=Rpl3 PE=1 SV=3	P27659	46 kDa	4	4	9	9	6	5	10	5	1.4	0.34
DNA mismatch repair protein Msh2 OS=Mus musculus GN=Msh2 PE=1 SV=1	P43247	104 kDa	5	4	8	14	7	5	9	8	1.4	0.24
ATP-dependent RNA helicase DDX1 OS=Mus musculus GN=Ddx1 PE=1 SV=1	Q91VR5	83 kDa	5	0	5	11	7	2	6	7	1.4	0.16
Thyroid hormone receptor-associated protein 3 OS=Mus musculus GN=Thrap3 PE=1 SV=1	Q569Z6	108 kDa	0	3	4	6	2	4	5	4	1.4	0.23
Mago nashi protein OS=Mus musculus GN=Magohb PE=1 SV=1	A0A023T778 (+2)	17 kDa	0	2	3	5	2	3	3	3	1.4	0.22
Chromodomain-helicase-DNA-binding protein 1 OS=Mus musculus GN=Chd1 PE=1 SV=3	P40201	196 kDa	0	0	3	3	2	2	3	2	1.4	0.064
C-terminal-binding protein 2 OS=Mus musculus GN=Ctbp2 PE=1 SV=2	P56546 (+1)	49 kDa	0	0	1	6	2	2	2	4	1.4	0.04
MCG21756, isoform CRA_b OS=Mus musculus GN=Nup205 PE=2 SV=1	B9EJ54	227 kDa	0	0	3	2	2	2	3	2	1.4	0.1
E3 ubiquitin-protein ligase NEDD4 OS=Mus musculus GN=Nedd4 PE=1 SV=3	P46935	103 kDa	0	0	3	3	2	2	3	2	1.4	0.064
Origin recognition complex subunit 3 OS=Mus musculus GN=Orc3 PE=1 SV=1	Q9JK30	82 kDa	0	0	3	3	2	2	3	2	1.4	0.064
Exosome complex component RRP46 OS=Mus musculus GN=Exosc5 PE=1 SV=1	Q9CRA8	25 kDa	0	0	0	6	2	2	2	4	1.4	0.064
Laminin subunit gamma-1 OS=Mus musculus GN=Lamc1 PE=1 SV=1	F8VQJ3	177 kDa	0	0	3	3	2	2	3	2	1.4	0.064
TOX high mobility group box family member 4 OS=Mus musculus GN=Tox4 PE=1 SV=3	Q8BU11	66 kDa	0	0	3	2	2	2	3	2	1.4	0.1
Protein Cenpf OS=Mus musculus GN=Cenpf PE=1 SV=1	E9Q3P4	342 kDa	0	0	0	6	2	2	2	4	1.4	0.064
Peptidyl-prolyl cis-trans isomerase (Fragment) OS=Mus musculus GN=Ppih PE=1 SV=1	A2BGI8	17 kDa	0	0	3	3	2	2	3	2	1.4	0.064
Protein 2310022A10Rik OS=Mus musculus GN=2310022A10Rik PE=1 SV=1	A0A087WRI8	43 kDa	0	0	3	2	2	2	3	2	1.4	0.1
Cellular nucleic acid-binding protein OS=Mus musculus GN=Cnbp PE=1 SV=2	P53996	20 kDa	0	0	3	2	2	2	3	2	1.4	0.1
Protein Dnajc21 OS=Mus musculus GN=Dnajc21 PE=1 SV=1	E9Q8D0	62 kDa	0	0	3	1	2	2	3	2	1.4	0.16
Serine/arginine-rich splicing factor 2 OS=Mus musculus GN=Srsf2 PE=1 SV=4	Q62093	25 kDa	0	0	3	1	2	2	3	2	1.4	0.16
TFIIH basal transcription factor complex helicase XPB subunit OS=Mus musculus GN=Ercc3 PE=2 SV=1	P49135	89 kDa	0	0	0	6	2	2	2	4	1.4	0.064
Replication factor C subunit 4 OS=Mus musculus GN=Rfc4 PE=1 SV=1	Q3UI84 (+1)	41 kDa	0	0	0	6	2	2	2	4	1.4	0.064
Serine/arginine-rich splicing factor 5 OS=Mus musculus GN=Srsf5 PE=1 SV=2	O35326 (+1)	31 kDa	0	0	0	6	2	2	2	4	1.4	0.064
F-actin-capping protein subunit alpha-1 OS=Mus musculus GN=Capza1 PE=1 SV=4	P47753	33 kDa	0	0	3	3	2	2	3	2	1.4	0.064
Monofunctional C1-tetrahydrofolate synthase, mitochondrial OS=Mus musculus GN=Mthfd1l PE=1 SV=2	Q3V3R1	106 kDa	0	0	3	3	2	2	3	2	1.4	0.064
Peptidyl-prolyl cis-trans isomerase A OS=Mus musculus GN=Ppia PE=1 SV=2	P17742	18 kDa	0	0	3	3	2	2	3	2	1.4	0.064
Pyruvate dehydrogenase E1 component subunit beta, mitochondrial OS=Mus musculus GN=Pdhb PE=1 SV=1	Q9D051	39 kDa	0	0	3	3	2	2	3	2	1.4	0.064
Protein Scaf11 OS=Mus musculus GN=Scaf11 PE=1 SV=1	E9PZM7	162 kDa	0	0	3	3	2	2	3	2	1.4	0.064
RNA polymerase-associated protein CTR9 homolog OS=Mus musculus GN=Ctr9 PE=1 SV=2	Q62018	133 kDa	0	0	3	2	2	2	3	2	1.4	0.1
Endoplasmin OS=Mus musculus GN=Hsp90b1 PE=1 SV=2	P08113	92 kDa	0	0	3	3	2	2	3	2	1.4	0.064
General transcription factor IIH subunit 4 OS=Mus musculus GN=Gtf2h4 PE=2 SV=1	O70422	52 kDa	0	0	0	6	2	2	2	4	1.4	0.064
DNA (cytosine-5)-methyltransferase 3A OS=Mus musculus GN=Dnmt3a PE=1 SV=2	O88508	102 kDa	0	0	0	6	2	2	2	4	1.4	0.064
Exosome component 10 OS=Mus musculus GN=Exosc10 PE=1 SV=2	P56960 (+1)	101 kDa	0	0	0	6	2	2	2	4	1.4	0.064
Nucleolar GTP-binding protein 2 OS=Mus musculus GN=Gnl2 PE=1 SV=2	Q99LH1	83 kDa	0	0	0	6	2	2	2	4	1.4	0.064
Zinc finger CCHC domain-containing protein 8 OS=Mus musculus GN=Zcchc8 PE=2 SV=3	Q9CYA6	78 kDa	0	0	0	6	2	2	2	4	1.4	0.064
Mortality factor 4-like protein 2 OS=Mus musculus GN=Morf4l2 PE=2 SV=1	Q9R0Q4	32 kDa	0	0	0	6	2	2	2	4	1.4	0.064
Apoptosis-inducing factor 1, mitochondrial OS=Mus musculus GN=Aifm1 PE=1 SV=1	B1AU25 (+1)	66 kDa	0	0	3	0	2	2	3	2	1.4	0.25
Elongation factor Tu OS=Mus musculus GN=Gm9755 PE=3 SV=1	D3YVN7 (+1)	50 kDa	0	0	3	0	2	2	3	2	1.4	0.25
14-3-3 protein theta (Fragment) OS=Mus musculus GN=Ywhaq PE=1 SV=1	F6VW30 (+2)	34 kDa	0	0	0	6	2	2	2	4	1.4	0.064
Structural maintenance of chromosomes protein 5 OS=Mus musculus GN=Smc5 PE=1 SV=1	Q8CG46	129 kDa	0	0	3	0	2	2	3	2	1.4	0.25

Stomatin-like protein 2, mitochondrial OS=Mus musculus GN=Stoml2 PE=1 SV=1	Q99JB2	38 kDa	0	0	3	0	2	2	3	2	1.4	0.25
Hypermethylated in cancer 2 protein OS=Mus musculus GN=Hic2 PE=2 SV=4	Q9JLZ6	67 kDa	0	0	3	0	2	2	3	2	1.4	0.25
Protein Zfp462 OS=Mus musculus GN=Zfp462 PE=1 SV=1	B1AWL2	283 kDa	0	0	3	0	2	2	3	2	1.4	0.25
Glypican 6, isoform CRA_a OS=Mus musculus GN=Gpc6 PE=1 SV=1	Q3V1C9 (+2)	63 kDa	0	0	3	0	2	2	3	2	1.4	0.25
Cytochrome c oxidase subunit 5B, mitochondrial OS=Mus musculus GN=Cox5b PE=1 SV=1	P19536 (+1)	14 kDa	0	0	3	0	2	2	3	2	1.4	0.25
ATP-dependent 6-phosphofructokinase, liver type OS=Mus musculus GN=Pfkl PE=1 SV=4	P12382	85 kDa	0	0	3	0	2	2	3	2	1.4	0.25
Guanine nucleotide-binding protein G(I)/G(S)/G(T) subunit beta-1 OS=Mus musculus GN=Gnb1 PE=1 SV=3	P62874 (+1)	37 kDa	0	0	3	0	2	2	3	2	1.4	0.25
Spindlin-1 OS=Mus musculus GN=Spin1 PE=1 SV=2	Q61142	30 kDa	0	0	3	0	2	2	3	2	1.4	0.25
Nucleoporin Nup37 OS=Mus musculus GN=Nup37 PE=1 SV=2	Q9CWU9	37 kDa	0	0	3	0	2	2	3	2	1.4	0.25
Elongation factor 1-beta OS=Mus musculus GN=Eef1b2 PE=1 SV=1	A0A087WS46 (+1)	20 kDa	0	0	3	0	2	2	3	2	1.4	0.25
Eukaryotic translation initiation factor 4 gamma 2 OS=Mus musculus GN=Eif4g2 PE=1 SV=1	F7CBP1 (+2)	98 kDa	0	0	3	0	2	2	3	2	1.4	0.25
Electron transfer flavoprotein subunit alpha, mitochondrial OS=Mus musculus GN=Etfa PE=1 SV=2	Q99LC5	35 kDa	0	0	3	0	2	2	3	2	1.4	0.25
Nucleophosmin OS=Mus musculus GN=Npm1 PE=1 SV=1	Q61937	33 kDa	12	22	25	53	17	29	29	32	1.3	0.092
U3 small nucleolar RNA-interacting protein 2 OS=Mus musculus GN=Rrp9 PE=1 SV=1	Q91WM3	52 kDa	7	6	10	19	10	8	11	11	1.3	0.27
ATP-dependent RNA helicase DDX24 OS=Mus musculus GN=Ddx24 PE=1 SV=1	F8WJA0 (+1)	101 kDa	8	2	9	12	11	3	10	7	1.3	0.37
Histone deacetylase 1 OS=Mus musculus GN=Hdac1 PE=1 SV=1	O09106	55 kDa	4	5	6	15	6	6	7	9	1.3	0.28
60 kDa heat shock protein, mitochondrial OS=Mus musculus GN=Hspd1 PE=1 SV=1	P63038	61 kDa	6	3	10	7	9	4	11	4	1.3	0.5
Apoptotic chromatin condensation inducer in the nucleus (Fragment) OS=Mus musculus GN=Acin1 PE=1 SV=1	F6RJ39 (+1)	144 kDa	3	3	3	12	4	4	3	7	1.3	0.29
17beta-hydroxysteroid dehydrogenase type 10/short chain L-3-hydroxyacyl-CoA dehydrogenase OS=Mus musculus GN=Hsd17b10 PE=2 SV=1	Q99N15	27 kDa	7	0	10	6	10	2	11	4	1.3	0.34
Protein Rbmxl2 OS=Mus musculus GN=Rbmxl2 PE=1 SV=1	Q9DAE2	42 kDa	0	6	4	13	2	8	5	8	1.3	0.2
Ribosome biogenesis protein BRX1 homolog OS=Mus musculus GN=Brix1 PE=1 SV=3	Q9DCA5	41 kDa	0	1	2	5	2	2	2	3	1.3	0.14
Active regulator of SIRT1 OS=Mus musculus GN=Rps19bp1 PE=1 SV=1	Q8C6B9	16 kDa	0	3	5	3	2	4	6	2	1.3	0.38
40S ribosomal protein S25 OS=Mus musculus GN=Rps25 PE=1 SV=1	P62852 (+1)	14 kDa	0	0	2	5	2	2	2	3	1.3	0.04
WD repeat-containing protein 76 OS=Mus musculus GN=Wdr76 PE=1 SV=1	A6PWY4	69 kDa	0	0	2	5	2	2	2	3	1.3	0.04
Heterogeneous nuclear ribonucleoprotein U-like protein 1 OS=Mus musculus GN=Hnrnpul1 PE=1 SV=1	Q8VDM6	96 kDa	0	0	2	5	2	2	2	3	1.3	0.04
Protein Dnajc13 OS=Mus musculus GN=Dnajc13 PE=1 SV=1	D4AFX7 (+1)	255 kDa	0	0	1	5	2	2	2	3	1.3	0.064
Uncharacterized protein C7orf50 homolog OS=Mus musculus PE=1 SV=3	Q9CXL3	22 kDa	0	0	2	5	2	2	2	3	1.3	0.04
Heat shock 70 kDa protein 4 OS=Mus musculus GN=Hspa4 PE=1 SV=1	Q3U2G2 (+1)	94 kDa	0	0	0	5	2	2	2	3	1.3	0.1
Ubiquitin carboxyl-terminal hydrolase 7 OS=Mus musculus GN=Usp7 PE=1 SV=1	E9PXY8 (+2)	133 kDa	0	0	0	5	2	2	2	3	1.3	0.1
Nucleoprotein TPR OS=Mus musculus GN=Tpr PE=1 SV=1	F6ZDS4 (+1)	274 kDa	0	0	0	5	2	2	2	3	1.3	0.1
Importin-5 OS=Mus musculus GN=Ipo5 PE=1 SV=3	Q8BKC5	124 kDa	0	0	0	5	2	2	2	3	1.3	0.1
Nucleolar protein 16 OS=Mus musculus GN=Nop16 PE=1 SV=1	Q9CPT5	21 kDa	0	0	0	5	2	2	2	3	1.3	0.1
RNA-binding protein PNO1 OS=Mus musculus GN=Pno1 PE=1 SV=1	Q9CPS7	27 kDa	0	0	0	5	2	2	2	3	1.3	0.1
Ubiquitin carboxyl-terminal hydrolase 48 OS=Mus musculus GN=Usp48 PE=1 SV=1	A2ALR9	119 kDa	0	0	0	5	2	2	2	3	1.3	0.1
T-complex protein 1 subunit gamma OS=Mus musculus GN=Cct3 PE=1 SV=1	E9Q133 (+2)	57 kDa	0	0	0	5	2	2	2	3	1.3	0.1
Zinc finger protein 106 OS=Mus musculus GN=Znf106 PE=1 SV=3	O88466	209 kDa	0	0	0	5	2	2	2	3	1.3	0.1
Non-histone chromosomal protein HMG-14 OS=Mus musculus GN=Hmgn1 PE=1 SV=2	P18608	10 kDa	0	0	0	5	2	2	2	3	1.3	0.1
60S ribosomal protein L32 OS=Mus musculus GN=Rpl32 PE=1 SV=2	P62911	16 kDa	0	0	0	5	2	2	2	3	1.3	0.1
Protein FAM98B OS=Mus musculus GN=Fam98b PE=1 SV=1	Q80VD1	45 kDa	0	0	0	5	2	2	2	3	1.3	0.1
SAFB-like transcription modulator OS=Mus musculus GN=Sltm PE=1 SV=1	Q8CH25	117 kDa	0	0	0	5	2	2	2	3	1.3	0.1
ATP-dependent RNA helicase DHX36 OS=Mus musculus GN=Dhx36 PE=1 SV=2	Q8VHK9	114 kDa	0	0	0	5	2	2	2	3	1.3	0.1
Regulator of nonsense transcripts 1 OS=Mus musculus GN=Upf1 PE=1 SV=2	Q9EPU0	124 kDa	0	0	0	5	2	2	2	3	1.3	0.1
Histone H1.0 OS=Mus musculus GN=H1f0 PE=2 SV=4	P10922	21 kDa	0	0	0	5	2	2	2	3	1.3	0.1
Glutamate dehydrogenase 1, mitochondrial OS=Mus musculus GN=Glud1 PE=1 SV=1	P26443	61 kDa	0	0	0	5	2	2	2	3	1.3	0.1
DNA replication licensing factor MCM4 OS=Mus musculus GN=Mcm4 PE=1 SV=1	P49717	97 kDa	0	0	0	5	2	2	2	3	1.3	0.1
Cyclin-dependent kinase 12 OS=Mus musculus GN=Cdk12 PE=1 SV=2	Q14AX6	164 kDa	0	0	0	5	2	2	2	3	1.3	0.1
Myosin regulatory light chain 12B OS=Mus musculus GN=Myl12b PE=1 SV=2	Q3THE2 (+1)	20 kDa	0	0	0	5	2	2	2	3	1.3	0.1
E3 ubiquitin/ISG15 ligase TRIM25 OS=Mus musculus GN=Trim25 PE=4 SV=1	Q5SU71 (+1)	71 kDa	0	0	0	5	2	2	2	3	1.3	0.1
Cytoskeleton-associated protein 4 OS=Mus musculus GN=Ckap4 PE=1 SV=2	Q8BMK4	64 kDa	0	0	0	5	2	2	2	3	1.3	0.1
Myosin light polypeptide 6 OS=Mus musculus GN=Myl6 PE=1 SV=3	Q60605	17 kDa	0	0	0	5	2	2	2	3	1.3	0.1
RNA-binding protein 3 OS=Mus musculus GN=Rbm3 PE=2 SV=1	O89086 (+1)	17 kDa	0	0	0	5	2	2	2	3	1.3	0.1
Sister chromatid cohesion protein PDS5 homolog B OS=Mus musculus GN=Pds5b PE=1 SV=1	F8WHU5 (+1)	165 kDa	0	0	0	5	2	2	2	3	1.3	0.1
40S ribosomal protein S9 OS=Mus musculus GN=Rps9 PE=1 SV=3	Q6ZWN5	23 kDa	6	5	10	11	9	6	11	7	1.2	0.47
Heterogeneous nuclear ribonucleoprotein M OS=Mus musculus GN=Hnrnpm PE=1 SV=3	Q9D0E1	78 kDa	16	29	37	51	23	38	43	31	1.2	0.27
Treacle protein OS=Mus musculus GN=Tcof1 PE=1 SV=1	H3BL37 (+1)	139 kDa	12	12	19	30	17	16	22	18	1.2	0.28
RuvB-like 1 OS=Mus musculus GN=Ruvbl1 PE=1 SV=1	P60122	50 kDa	8	9	16	16	11	12	18	10	1.2	0.44
Guanine nucleotide-binding protein-like 3 OS=Mus musculus GN=Gnl3 PE=1 SV=2	Q8CI11	61 kDa	6	8	10	20	9	10	11	12	1.2	0.3
Probable ATP-dependent RNA helicase DDX56 OS=Mus musculus GN=Ddx56 PE=2 SV=1	Q9D0R4	61 kDa	8	7	10	20	11	9	11	12	1.2	0.38
Glutamate-rich WD repeat-containing protein 1 OS=Mus musculus GN=Grwd1 PE=1 SV=2	Q810D6	49 kDa	6	7	11	14	9	9	13	8	1.2	0.44
RNA binding motif protein, X-linked-like-1 OS=Mus musculus GN=Rbmxl1 PE=2 SV=1	Q91VM5	42 kDa	5	5	8	12	7	6	9	7	1.2	0.42
Ribosomal protein OS=Mus musculus GN=Rpl10a PE=1 SV=1	Q5XJF6	25 kDa	7	3	4	21	10	4	5	13	1.2	0.2
40S ribosomal protein S18 OS=Mus musculus GN=Rps18 PE=1 SV=3	P62270	18 kDa	4	7	9	12	6	9	10	7	1.2	0.47
60S ribosomal protein L17 OS=Mus musculus GN=Rpl17 PE=1 SV=1	Q6ZWZ7 (+1)	21 kDa	2	4	4	9	3	5	5	5	1.2	0.42
Lupus La protein homolog OS=Mus musculus GN=Ssb PE=1 SV=1	P32067	48 kDa	3	2	0	10	4	3	2	6	1.2	0.5
Histone H2A.Z OS=Mus musculus GN=H2afz PE=1 SV=2	P0C0S6 (+1)	14 kDa	8	5	0	33	11	6	2	20	1.2	0.15
Filamin, alpha OS=Mus musculus GN=Flna PE=1 SV=1	B7FAU9 (+2)	280 kDa	1	0	2	4	2	2	2	2	1.2	0.2
DDB1- and CUL4-associated factor 13 OS=Mus musculus GN=Dcaf13 PE=2 SV=2	Q6PAC3	51 kDa	0	3	3	6	2	4	3	4	1.2	0.3
60S ribosomal protein L8 OS=Mus musculus GN=Rpl8 PE=1 SV=2	P62918	28 kDa	0	0	2	4	2	2	2	2	1.2	0.064
60S ribosomal protein L9 OS=Mus musculus GN=Rpl9 PE=1 SV=2	P51410	22 kDa	3	0	0	9	4	2	2	5	1.2	0.3
Serine/threonine-protein kinase VRK1 OS=Mus musculus GN=Vrk1 PE=1 SV=2	Q80X41	50 kDa	0	3	0	8	2	4	2	5	1.2	0.38
60S ribosome subunit biogenesis protein NIP7 homolog OS=Mus musculus GN=Nip7 PE=1 SV=1	Q9CXK8	20 kDa	2	0	0	6	3	2	2	4	1.2	0.39
Transformer-2 protein homolog beta OS=Mus musculus GN=Tra2b PE=1 SV=1	F8WJG3 (+1)	22 kDa	0	2	0	6	2	3	2	4	1.2	0.39
39S ribosomal protein L12, mitochondrial OS=Mus musculus GN=Mrpl12 PE=1 SV=2	Q9DB15	22 kDa	0	2	3	2	2	3	3	2	1.2	0.49
Zinc finger protein 292 OS=Mus musculus GN=Zfp292 PE=1 SV=2	Q9Z2U2	301 kDa	0	0	2	4	2	2	2	2	1.2	0.064
RNA-binding protein with serine-rich domain 1 OS=Mus musculus GN=Rnps1 PE=1 SV=1	Q99M28 (+1)	34 kDa	0	2	0	6	2	3	2	4	1.2	0.39
Trifunctional purine biosynthetic protein adenosine-3 OS=Mus musculus GN=Gart PE=1 SV=3	Q64737	108 kDa	0	0	2	4	2	2	2	2	1.2	0.064
Interferon-stimulated 20 kDa exonuclease-like 2 OS=Mus musculus GN=Isg20l2 PE=2 SV=2	Q3U1G5	41 kDa	0	0	2	4	2	2	2	2	1.2	0.064
Protein FAM207A OS=Mus musculus GN=Fam207a PE=1 SV=1	P58468	25 kDa	0	0	2	4	2	2	2	2	1.2	0.064
Atherin OS=Mus musculus GN=Samd1 PE=1 SV=1	D3YXK1	55 kDa	0	0	2	4	2	2	2	2	1.2	0.064
Heterogeneous nuclear ribonucleoprotein U OS=Mus musculus GN=Hnrnpu PE=1 SV=1	Q8VEK3	88 kDa	15	20	24	38	21	26	28	23	1.1	0.49
SWI/SNF-related matrix-associated actin-dependent regulator of chromatin subfamily A member 5 OS=Mus musculus GN=Smarca5 PE=1 SV=1	Q91ZW3	122 kDa	9	17	15	37	13	22	17	22	1.1	0.3
Nucleolar protein 58 OS=Mus musculus GN=Nop58 PE=1 SV=1	Q6DFW4	60 kDa	19	10	20	38	27	13	23	23	1.1	0.29
Ribosomal L1 domain-containing protein 1 OS=Mus musculus GN=Rsl1d1 PE=1 SV=1	Q8BVY0	50 kDa	10	17	18	30	14	22	21	18	1.1	0.49
Glyceraldehyde-3-phosphate dehydrogenase OS=Mus musculus GN=Gapdh PE=1 SV=1	A0A0A0MQF6 (+2)	39 kDa	12	9	16	20	17	12	18	12	1.1	0.55
Histone-binding protein RBBP4 OS=Mus musculus GN=Rbbp4 PE=1 SV=5	Q60972	48 kDa	8	6	12	13	11	8	14	8	1.1	0.52
H/ACA ribonucleoprotein complex subunit 4 OS=Mus musculus GN=Dkc1 PE=1 SV=4	Q9ESX5	57 kDa	9	5	7	23	13	6	8	14	1.1	0.3
Serine/arginine repetitive matrix protein 2 OS=Mus musculus GN=Srrm2 PE=1 SV=3	Q8BTI8	295 kDa	8	5	9	16	11	6	10	10	1.1	0.44
E3 ubiquitin-protein ligase UHRF1 OS=Mus musculus GN=Uhrf1 PE=1 SV=2	Q8VDF2	88 kDa	10	3	10	14	14	4	11	8	1.1	0.49
RuvB-like 2 OS=Mus musculus GN=Ruvbl2 PE=2 SV=3	Q9WTM5	51 kDa	4	9	10	12	6	12	11	7	1.1	0.55
Probable ATP-dependent RNA helicase DDX47 OS=Mus musculus GN=Ddx47 PE=2 SV=2	Q9CWX9	51 kDa	6	5	6	15	9	6	7	9	1.1	0.47
40S ribosomal protein S2 OS=Mus musculus GN=Rps2 PE=1 SV=3	P25444	31 kDa	3	7	7	11	4	9	8	7	1.1	0.54
NHP2-like protein 1 OS=Mus musculus GN=Snu13 PE=1 SV=4	Q9D0T1	14 kDa	3	7	7	10	4	9	8	6	1.1	0.56
Parafibromin OS=Mus musculus GN=Cdc73 PE=1 SV=1	Q8JZM7	61 kDa	7	0	7	8	10	2	8	5	1.1	0.4
Bcl-2-associated transcription factor 1 OS=Mus musculus GN=Bclaf1 PE=1 SV=2	Q8K019	106 kDa	3	5	6	8	4	6	7	5	1.1	0.58
60S ribosomal protein L27a OS=Mus musculus GN=Rpl27a PE=1 SV=5	P14115	17 kDa	4	0	4	7	6	2	5	4	1.1	0.3
Digestive organ expansion factor homolog OS=Mus musculus GN=Diexf PE=2 SV=2	Q8BTT6	89 kDa	3	1	4	4	4	2	5	2	1.1	0.53
Activity-dependent neuroprotector homeobox protein OS=Mus musculus GN=Adnp PE=1 SV=2	Q9Z103	92 kDa	2	0	3	3	3	2	3	2	1.1	0.39
Protein virilizer homolog OS=Mus musculus GN=Kiaa1429 PE=1 SV=1	A2AIV2 (+1)	201 kDa	0	0	0	4	2	2	2	2	1.1	0.16
60S ribosomal protein L37a OS=Mus musculus GN=Rpl37a PE=1 SV=2	P61514	10 kDa	0	3	0	7	2	4	2	4	1.1	0.46
SWI/SNF-related matrix-associated actin-dependent regulator of chromatin subfamily D member 1 OS=Mus musculus GN=Smarcd1 PE=1 SV=3	Q61466	58 kDa	0	0	2	3	2	2	2	2	1.1	0.1
Pre-mRNA 3'-end-processing factor FIP1 OS=Mus musculus GN=Fip1l1 PE=1 SV=1	D3Z4V2 (+1)	67 kDa	0	1	0	4	2	2	2	2	1.1	0.39
Activator of basal transcription 1 OS=Mus musculus GN=Abt1 PE=2 SV=1	Q9QYL7	31 kDa	0	0	1	4	2	2	2	2	1.1	0.1
Apolipoprotein E OS=Mus musculus GN=Apoe PE=1 SV=2	P08226	36 kDa	0	0	1	4	2	2	2	2	1.1	0.1

Exosome complex exonuclease RRP42 OS=Mus musculus GN=Exosc7 PE=1 SV=2	Q9D0M0	32 kDa	0	0	2	3	2	2	2	2	1.1	0.1
DNA-directed RNA polymerase, mitochondrial OS=Mus musculus GN=Polrmt PE=2 SV=1	Q8BKF1	137 kDa	1	0	0	4	2	2	2	2	1.1	0.39
UPF0688 protein C1orf174 homolog OS=Mus musculus PE=1 SV=1	Q80WR5	25 kDa	0	3	4	0	2	4	5	2	1.1	0.51
Uncharacterized protein C1orf131 homolog OS=Mus musculus PE=1 SV=1	Q8CIL4	31 kDa	0	0	2	2	2	2	2	2	1.1	0.16
Nucleolar MIF4G domain-containing protein 1 OS=Mus musculus GN=Nom1 PE=1 SV=2	Q3UFM5	96 kDa	0	0	2	2	2	2	2	2	1.1	0.16
Pre-rRNA-processing protein TSR1 homolog OS=Mus musculus GN=Tsr1 PE=1 SV=1	Q5SWD9	92 kDa	0	1	0	4	2	2	2	2	1.1	0.39
60S ribosomal protein L34 OS=Mus musculus GN=Rpl34 PE=1 SV=2	Q9D1R9	13 kDa	0	0	0	4	2	2	2	2	1.1	0.16
Protein nepro OS=Mus musculus GN=Nepro PE=2 SV=1	Q8R2U2	63 kDa	0	0	2	3	2	2	2	2	1.1	0.1
Elongation factor 1-delta (Fragment) OS=Mus musculus GN=Eef1d PE=1 SV=1	E9QN08 (+6)	27 kDa	0	0	2	3	2	2	2	2	1.1	0.1
Histone-lysine N-methyltransferase EHMT1 OS=Mus musculus GN=Ehmt1 PE=1 SV=2	Q5DW34 (+1)	142 kDa	0	0	2	3	2	2	2	2	1.1	0.1
THO complex subunit 6 homolog OS=Mus musculus GN=Thoc6 PE=1 SV=1	D3Z132 (+1)	37 kDa	0	0	2	1	2	2	2	2	1.1	0.25
PHD finger protein 3 OS=Mus musculus GN=Phf3 PE=1 SV=1	B2RQG2	226 kDa	0	0	0	4	2	2	2	2	1.1	0.16
ATP-dependent RNA helicase DDX55 OS=Mus musculus GN=Ddx55 PE=1 SV=2	Q6ZPL9	68 kDa	0	0	0	4	2	2	2	2	1.1	0.16
H1 histone family, member X OS=Mus musculus GN=H1fx PE=1 SV=1	Q80ZM5	20 kDa	0	0	0	4	2	2	2	2	1.1	0.16
Spermatid perinuclear RNA-binding protein OS=Mus musculus GN=Strbp PE=1 SV=1	Q91WM1	74 kDa	0	0	0	4	2	2	2	2	1.1	0.16
60S ribosomal protein L35 OS=Mus musculus GN=Rpl35 PE=1 SV=1	Q6ZWV7	15 kDa	0	0	0	4	2	2	2	2	1.1	0.16
Cullin-4B OS=Mus musculus GN=Cul4b PE=1 SV=1	A2A432	111 kDa	0	0	0	4	2	2	2	2	1.1	0.16
DNA-directed RNA polymerase I subunit RPA2 OS=Mus musculus GN=Polr1b PE=3 SV=1	A2AP84 (+1)	86 kDa	0	0	0	4	2	2	2	2	1.1	0.16
Importin subunit alpha-4 OS=Mus musculus GN=Kpna3 PE=1 SV=1	O35344	58 kDa	0	0	0	4	2	2	2	2	1.1	0.16
Centromere protein C OS=Mus musculus GN=Cenpc PE=1 SV=2	P49452	102 kDa	0	0	0	4	2	2	2	2	1.1	0.16
Serine/threonine-protein kinase PRP4 homolog OS=Mus musculus GN=Prpf4b PE=1 SV=3	Q61136	117 kDa	0	0	0	4	2	2	2	2	1.1	0.16
Paraspeckle component 1 OS=Mus musculus GN=Pspc1 PE=1 SV=1	Q8R326	59 kDa	0	0	0	4	2	2	2	2	1.1	0.16
Sentrin-specific protease 3 OS=Mus musculus GN=Senp3 PE=1 SV=1	Q9EP97	64 kDa	0	0	0	4	2	2	2	2	1.1	0.16
X-ray repair cross-complementing protein 5 OS=Mus musculus GN=Xrcc5 PE=1 SV=4	P27641	83 kDa	0	0	0	4	2	2	2	2	1.1	0.16
DnaJ homolog subfamily A member 2 OS=Mus musculus GN=Dnaja2 PE=1 SV=1	Q9QYJ0	46 kDa	0	0	0	4	2	2	2	2	1.1	0.16
Mitochondrial Rho GTPase 1 OS=Mus musculus GN=Rhot1 PE=1 SV=1	Q8BG51	72 kDa	0	0	0	4	2	2	2	2	1.1	0.16
Chromatin modification-related protein MEAF6 OS=Mus musculus GN=Meaf6 PE=1 SV=1	Q2VPQ9 (+1)	22 kDa	0	0	0	4	2	2	2	2	1.1	0.16
Testis-expressed sequence 10 protein OS=Mus musculus GN=Tex10 PE=1 SV=1	Q3URQ0	105 kDa	0	0	0	4	2	2	2	2	1.1	0.16
Talin-1 OS=Mus musculus GN=Tln1 PE=1 SV=2	P26039	270 kDa	0	0	2	0	2	2	2	2	1.1	0.4
Protein Prdm2 OS=Mus musculus GN=Prdm2 PE=1 SV=1	A2A7B5	187 kDa	0	0	0	4	2	2	2	2	1.1	0.16
RNA exonuclease 4 (Fragment) OS=Mus musculus GN=Rexo4 PE=1 SV=1	A2ALB1 (+2)	40 kDa	0	0	0	4	2	2	2	2	1.1	0.16
Histone acetyltransferase type B catalytic subunit OS=Mus musculus GN=Hat1 PE=1 SV=1	A2ATU9 (+1)	50 kDa	0	0	0	4	2	2	2	2	1.1	0.16
rRNA-processing protein FCF1 homolog OS=Mus musculus GN=Fcf1 PE=1 SV=1	E9Q186 (+1)	22 kDa	0	0	0	4	2	2	2	2	1.1	0.16
Transcription intermediary factor 1-alpha OS=Mus musculus GN=Trim24 PE=1 SV=1	E9Q1U8 (+1)	110 kDa	0	0	0	4	2	2	2	2	1.1	0.16
T-complex protein 1 subunit delta OS=Mus musculus GN=Cct4 PE=1 SV=1	G5E839 (+1)	55 kDa	0	0	0	4	2	2	2	2	1.1	0.16
Eukaryotic translation initiation factor 3 subunit D OS=Mus musculus GN=Eif3d PE=1 SV=2	O70194	64 kDa	0	0	0	4	2	2	2	2	1.1	0.16
T-complex protein 1 subunit beta OS=Mus musculus GN=Cct2 PE=1 SV=4	P80314	57 kDa	0	0	0	4	2	2	2	2	1.1	0.16
Growth/differentiation factor 3 OS=Mus musculus GN=Gdf3 PE=2 SV=2	Q07104	42 kDa	0	0	0	4	2	2	2	2	1.1	0.16
Zinc finger and SCAN domain containing protein 4C OS=Mus musculus GN=Zscan4c PE=2 SV=1	Q80VJ6	58 kDa	0	0	0	4	2	2	2	2	1.1	0.16
Aconitate hydratase, mitochondrial OS=Mus musculus GN=Aco2 PE=1 SV=1	Q99KI0	85 kDa	0	0	0	4	2	2	2	2	1.1	0.16
Probable U3 small nucleolar RNA-associated protein 11 OS=Mus musculus GN=Utp11l PE=2 SV=1	Q9CZJ1	31 kDa	0	0	0	4	2	2	2	2	1.1	0.16
Nucleosome assembly protein 1-like 1 OS=Mus musculus GN=Nap1l1 PE=1 SV=1	E9PW66 (+1)	49 kDa	0	0	0	4	2	2	2	2	1.1	0.16
Nuclear pore complex protein Nup98-Nup96 OS=Mus musculus GN=Nup98 PE=1 SV=2	Q6PFD9	197 kDa	0	0	0	4	2	2	2	2	1.1	0.16
Acidic leucine-rich nuclear phosphoprotein 32 family member B OS=Mus musculus GN=Anp32b PE=1 SV=1	Q9EST5	31 kDa	0	0	0	4	2	2	2	2	1.1	0.16
Kinesin-like protein KIF18B OS=Mus musculus GN=Kif18b PE=2 SV=2	Q6PFD6	92 kDa	0	0	0	4	2	2	2	2	1.1	0.16
Aspartyl/asparaginyl beta-hydroxylase OS=Mus musculus GN=Asph PE=1 SV=1	A2AL85 (+2)	81 kDa	0	0	2	0	2	2	2	2	1.1	0.4
Fatty acid synthase OS=Mus musculus GN=Fasn PE=1 SV=2	P19096 (+1)	272 kDa	0	0	2	0	2	2	2	2	1.1	0.4
Structural maintenance of chromosomes protein 2 OS=Mus musculus GN=Smc2 PE=1 SV=2	Q8CG48	134 kDa	0	0	2	0	2	2	2	2	1.1	0.4
Zinc finger protein 280C OS=Mus musculus GN=Znf280c PE=1 SV=1	Q6P3Y5	83 kDa	0	0	2	0	2	2	2	2	1.1	0.4
Kinesin-like protein OS=Mus musculus GN=Kif2a PE=1 SV=1	E0CZ72 (+1)	84 kDa	0	0	2	0	2	2	2	2	1.1	0.4
Zinc finger protein 346 OS=Mus musculus GN=Zfp346 PE=1 SV=1	E9PYN2 (+1)	29 kDa	0	0	2	0	2	2	2	2	1.1	0.4
Growth factor receptor-bound protein 2 OS=Mus musculus GN=Grb2 PE=1 SV=1	Q60631	25 kDa	0	0	2	0	2	2	2	2	1.1	0.4
Centrosomal protein of 55 kDa OS=Mus musculus GN=Cep55 PE=1 SV=2	Q8BT07	54 kDa	0	0	2	0	2	2	2	2	1.1	0.4
Protein SEC13 homolog OS=Mus musculus GN=Sec13 PE=1 SV=3	Q9D1M0	36 kDa	0	0	2	0	2	2	2	2	1.1	0.4
Mitochondrial ribonuclease P protein 1 OS=Mus musculus GN=Trmt10c PE=1 SV=2	Q3UFY8	48 kDa	0	0	2	0	2	2	2	2	1.1	0.4
Protein Wiz (Fragment) OS=Mus musculus GN=Wiz PE=1 SV=1	F6ZBR8 (+2)	105 kDa	0	0	2	0	2	2	2	2	1.1	0.4
Nucleolin OS=Mus musculus GN=Ncl PE=1 SV=2	P09405	77 kDa	29	28	35	64	41	36	40	39	1	0.51
E3 ubiquitin-protein ligase TRIP12 OS=Mus musculus GN=Trip12 PE=1 SV=1	G5E870	224 kDa	28	15	18	62	40	19	21	37	1	0.37
Heterogeneous nuclear ribonucleoprotein K OS=Mus musculus GN=Hnrnpk PE=1 SV=1	P61979	51 kDa	30	13	25	50	43	17	29	30	1	0.51
Histone H1.2 OS=Mus musculus GN=Hist1h1c PE=1 SV=2	P15864	21 kDa	16	16	21	31	23	21	24	19	1	0.44
PC4 and SFRS1-interacting protein OS=Mus musculus GN=Psip1 PE=1 SV=1	Q99JF8	60 kDa	9	11	13	22	13	14	15	13	1	0.53
U3 small nucleolar RNA-associated protein 14 homolog A OS=Mus musculus GN=Utp14a PE=1 SV=1	Q640M1	87 kDa	9	6	8	18	13	8	9	11	1	0.56
40S ribosomal protein S8 OS=Mus musculus GN=Rps8 PE=1 SV=2	P62242	24 kDa	7	7	10	11	10	9	11	7	1	0.41
Tubulin alpha-1B chain OS=Mus musculus GN=Tuba1b PE=1 SV=2	P05213	50 kDa	8	5	9	12	11	6	10	7	1	0.5
Ribosome biogenesis protein WDR12 OS=Mus musculus GN=Wdr12 PE=2 SV=1	Q9JJA4	47 kDa	7	3	5	14	10	4	6	8	1	0.48
ATP synthase subunit O, mitochondrial OS=Mus musculus GN=Atp5o PE=1 SV=1	Q9DB20	23 kDa	7	5	6	15	10	6	7	9	1	0.56
60S ribosomal protein L23a OS=Mus musculus GN=Rpl23a PE=1 SV=1	P62751	18 kDa	6	6	7	15	9	8	8	9	1	0.5
Polymerase delta-interacting protein 3 OS=Mus musculus GN=Poldip3 PE=1 SV=1	Q8BG81	46 kDa	3	8	8	8	4	10	9	5	1	0.41
Nucleolar transcription factor 1 OS=Mus musculus GN=Ubtf PE=1 SV=1	A2AWT5 (+3)	89 kDa	4	1	2	9	6	2	2	5	1	0.43
60S ribosomal protein L30 OS=Mus musculus GN=Rpl30 PE=1 SV=2	P62889	13 kDa	5	4	7	8	7	5	8	5	1	0.55
Protein DEK OS=Mus musculus GN=Dek PE=1 SV=1	Q7TNV0	43 kDa	4	4	7	4	6	5	8	2	1	0.4
40S ribosomal protein S16 OS=Mus musculus GN=Rps16 PE=1 SV=4	P14131	16 kDa	0	5	5	5	2	6	6	3	1	0.5
RNA-binding protein 27 OS=Mus musculus GN=Rbm27 PE=1 SV=3	Q5SFM8	119 kDa	0	4	3	6	2	5	3	4	1	0.45
tRNA-splicing ligase RtcB homolog OS=Mus musculus GN=Rtcb PE=1 SV=1	Q99LF4	55 kDa	0	3	0	6	2	4	2	4	1	0.56
DNA-directed RNA polymerase II subunit RPB3 OS=Mus musculus GN=Polr2c PE=1 SV=1	Q99M46	31 kDa	0	3	3	4	2	4	3	2	1	0.46
Eukaryotic translation initiation factor 4 gamma 1 OS=Mus musculus GN=Eif4g1 PE=1 SV=1	E9PVC5 (+3)	175 kDa	0	0	1	2	2	2	2	2	1	0.25
Integrator complex subunit 1 OS=Mus musculus GN=Ints1 PE=1 SV=2	K3W4P2 (+1)	248 kDa	1	0	0	2	2	2	2	2	1	0.69
Large subunit GTPase 1 homolog OS=Mus musculus GN=Lsg1 PE=1 SV=2	Q3UM18	73 kDa	0	0	1	2	2	2	2	2	1	0.25
DnaJ homolog subfamily B member 6 OS=Mus musculus GN=Dnajb6 PE=1 SV=4	O54946	40 kDa	0	0	1	3	2	2	2	2	1	0.16
Spermatogenesis-associated protein 5 OS=Mus musculus GN=Spata5 PE=1 SV=1	A0A0A0MQ80 (+1)	97 kDa	0	0	0	3	2	2	2	2	1	0.25
60S ribosomal protein L21 OS=Mus musculus GN=Rpl21 PE=1 SV=3	O09167 (+1)	19 kDa	0	0	0	3	2	2	2	2	1	0.25
Integrator complex subunit 3 OS=Mus musculus GN=Ints3 PE=2 SV=2	Q7TPD0	118 kDa	0	0	0	3	2	2	2	2	1	0.25
Sodium/potassium-transporting ATPase subunit alpha-1 OS=Mus musculus GN=Atp1a1 PE=1 SV=1	Q8VDN2	113 kDa	0	0	0	3	2	2	2	2	1	0.25
Aspartate--tRNA ligase, cytoplasmic OS=Mus musculus GN=Dars PE=1 SV=2	Q922B2	57 kDa	0	0	0	3	2	2	2	2	1	0.25
Sororin OS=Mus musculus GN=Cdca5 PE=1 SV=1	Q9CPY3	29 kDa	0	0	0	3	2	2	2	2	1	0.25
PHD and RING finger domain-containing protein 1 OS=Mus musculus GN=Phrf1 PE=2 SV=2	A6H619	184 kDa	0	0	0	3	2	2	2	2	1	0.25
Probable 28S rRNA (cytosine-C(5))-methyltransferase OS=Mus musculus GN=Nsun5 PE=2 SV=2	Q8K4F6	51 kDa	0	0	0	3	2	2	2	2	1	0.25
Bromodomain and PHD finger containing, 1 OS=Mus musculus GN=Brpf1 PE=2 SV=1	B2RRD7 (+3)	137 kDa	0	0	0	2	2	2	2	2	1	0.4
Tyrosine--tRNA ligase OS=Mus musculus GN=Yars PE=1 SV=1	A2A7S7 (+1)	63 kDa	0	0	0	2	2	2	2	2	1	0.4
Alanine--tRNA ligase, cytoplasmic OS=Mus musculus GN=Aars PE=1 SV=1	Q8BGQ7	107 kDa	0	0	0	3	2	2	2	2	1	0.25
Methionine--tRNA ligase, cytoplasmic OS=Mus musculus GN=Mars PE=3 SV=1	E9QB02 (+1)	102 kDa	0	0	0	3	2	2	2	2	1	0.25
Clathrin heavy chain OS=Mus musculus GN=Cltc PE=1 SV=1	Q5SXR6 (+1)	192 kDa	0	0	0	2	2	2	2	2	1	0.4
Protein Srcap OS=Mus musculus GN=Srcap PE=1 SV=1	A0A087WNL7 (+3)	343 kDa	0	0	0	3	2	2	2	2	1	0.25
Protein Arid2 OS=Mus musculus GN=Arid2 PE=1 SV=1	E9Q7E2	196 kDa	0	0	0	3	2	2	2	2	1	0.25
F-box and leucine-rich repeat protein 11 OS=Mus musculus GN=Kdm2a PE=1 SV=2	F6YRW4 (+1)	133 kDa	0	0	0	3	2	2	2	2	1	0.25
G2/mitotic-specific cyclin-B1 OS=Mus musculus GN=Ccnb1 PE=3 SV=1	G3UY65 (+1)	44 kDa	0	0	0	3	2	2	2	2	1	0.25
MBT domain-containing protein 1 OS=Mus musculus GN=Mbtd1 PE=1 SV=1	Q6P5G3	71 kDa	0	0	0	3	2	2	2	2	1	0.25
RNA-binding protein with multiple splicing 2 OS=Mus musculus GN=Rbpms2 PE=1 SV=1	Q8VC52 (+1)	22 kDa	0	0	0	3	2	2	2	2	1	0.25
DnaJ homolog subfamily C member 7 OS=Mus musculus GN=Dnajc7 PE=1 SV=2	Q9QYI3	56 kDa	0	0	0	3	2	2	2	2	1	0.25
Eukaryotic translation initiation factor 2A OS=Mus musculus GN=Eif2a PE=1 SV=2	Q8BJW6	64 kDa	0	0	0	3	2	2	2	2	1	0.25
Structural maintenance of chromosomes protein 3 OS=Mus musculus GN=Smc3 PE=1 SV=2	Q9CW03	142 kDa	0	0	0	2	2	2	2	2	1	0.4
Zinc finger CCCH domain-containing protein 11A OS=Mus musculus GN=Zc3h11a PE=2 SV=1	Q6NZF1	86 kDa	0	0	0	2	2	2	2	2	1	0.4
Exportin-1 OS=Mus musculus GN=Xpo1 PE=1 SV=1	Q6P5F9	123 kDa	0	0	0	2	2	2	2	2	1	0.4

Cyclin-dependent kinase 2-associated protein 1 OS=Mus musculus GN=Cdk2ap1 PE=1 SV=1	F2Z4B3 (+1)	10 kDa	0	0	0	3	2	2	2	2	1	0.25
Partner of Y14 and mago OS=Mus musculus GN=Pym1 PE=1 SV=2	Q8CHP5	23 kDa	0	0	0	2	2	2	2	2	1	0.4
Cell division cycle protein 27 homolog OS=Mus musculus GN=Cdc27 PE=1 SV=1	A2A6Q5	92 kDa	0	0	0	2	2	2	2	2	1	0.4
Shugoshin-like 1 OS=Mus musculus GN=Sgol1 PE=2 SV=1	Q9CXH7	59 kDa	0	0	0	3	2	2	2	2	1	0.25
Cullin-associated NEDD8-dissociated protein 1 OS=Mus musculus GN=Cand1 PE=1 SV=2	Q6ZQ38	136 kDa	0	0	0	2	2	2	2	2	1	0.4
E3 ubiquitin-protein ligase RBBP6 OS=Mus musculus GN=Rbbp6 PE=1 SV=5	P97868	200 kDa	0	0	0	3	2	2	2	2	1	0.25
Serine/arginine-rich-splicing factor 10 OS=Mus musculus GN=Srsf10 PE=2 SV=1	Q3TFP0 (+1)	22 kDa	0	0	0	3	2	2	2	2	1	0.25
Prohibitin-2 OS=Mus musculus GN=Phb2 PE=1 SV=1	O35129	33 kDa	0	0	0	3	2	2	2	2	1	0.25
Protein Iqgap3 OS=Mus musculus GN=Iqgap3 PE=4 SV=1	F8VQ29	185 kDa	0	0	0	3	2	2	2	2	1	0.25
Ribonuclease P protein subunit p38 OS=Mus musculus GN=Rpp38 PE=1 SV=1	A2AJG0 (+1)	31 kDa	0	0	0	2	2	2	2	2	1	0.4
Inactive serine/threonine-protein kinase VRK3 OS=Mus musculus GN=Vrk3 PE=1 SV=2	Q8K3G5	51 kDa	0	0	0	2	2	2	2	2	1	0.4
Baculoviral IAP repeat-containing protein 5 OS=Mus musculus GN=Birc5 PE=1 SV=1	O70201	16 kDa	0	0	0	3	2	2	2	2	1	0.25
Polycomb group RING finger protein 6 OS=Mus musculus GN=Pcgf6 PE=1 SV=1	Q99NA9	40 kDa	0	0	0	2	2	2	2	2	1	0.4
Rab GDP dissociation inhibitor beta OS=Mus musculus GN=Gdi2 PE=1 SV=1	Q61598	51 kDa	0	0	0	3	2	2	2	2	1	0.25
DNA helicase INO80 OS=Mus musculus GN=Ino80 PE=1 SV=2	Q6ZPV2	177 kDa	0	0	0	2	2	2	2	2	1	0.4
AT-rich interactive domain-containing protein 1A OS=Mus musculus GN=Arid1a PE=1 SV=1	A2BH40 (+1)	242 kDa	0	0	0	2	2	2	2	2	1	0.4
Cullin-1 OS=Mus musculus GN=Cul1 PE=1 SV=1	Q9WTX6	90 kDa	0	0	0	2	2	2	2	2	1	0.4
Nucleoporin-like protein 2 OS=Mus musculus GN=Nupl2 PE=1 SV=1	E9QL43 (+1)	44 kDa	0	0	0	3	2	2	2	2	1	0.25
Exosome complex component RRP40 (Fragment) OS=Mus musculus GN=Exosc3 PE=1 SV=1	F6TGV1 (+1)	15 kDa	0	0	0	3	2	2	2	2	1	0.25
Peptidyl-prolyl cis-trans isomerase FKBP8 OS=Mus musculus GN=Fkbp8 PE=1 SV=2	O35465	44 kDa	0	0	0	3	2	2	2	2	1	0.25
Ribosome biogenesis protein NSA2 homolog OS=Mus musculus GN=Nsa2 PE=2 SV=1	Q9CR47	30 kDa	0	0	0	3	2	2	2	2	1	0.25
Serine hydroxymethyltransferase OS=Mus musculus GN=Shmt2 PE=1 SV=1	Q9CZN7	56 kDa	0	0	0	3	2	2	2	2	1	0.25
Acylglycerol kinase, mitochondrial OS=Mus musculus GN=Agk PE=1 SV=1	Q9ESW4	47 kDa	0	0	0	3	2	2	2	2	1	0.25
Ribosomal protein L19 OS=Mus musculus GN=Rpl19 PE=1 SV=1	A2A547 (+1)	23 kDa	0	0	0	3	2	2	2	2	1	0.25
SUN domain-containing protein 1 OS=Mus musculus GN=Sun1 PE=1 SV=1	D3Z0V9 (+1)	85 kDa	0	0	0	3	2	2	2	2	1	0.25
Protein Gm17669 OS=Mus musculus GN=Gm17669 PE=4 SV=1	F6QL70 (+1)	17 kDa	0	0	0	3	2	2	2	2	1	0.25
Cold shock domain-containing protein E1 OS=Mus musculus GN=Csde1 PE=2 SV=1	Q91W50	89 kDa	0	0	0	3	2	2	2	2	1	0.25
28S ribosomal protein S22, mitochondrial OS=Mus musculus GN=Mrps22 PE=1 SV=1	Q9CXW2	41 kDa	0	0	0	3	2	2	2	2	1	0.25
Protein BUD31 homolog OS=Mus musculus GN=Bud31 PE=4 SV=1	E0CX20	17 kDa	0	0	0	3	2	2	2	2	1	0.25
UAP56-interacting factor OS=Mus musculus GN=Fyttd1 PE=1 SV=1	Q91Z49	36 kDa	0	0	0	3	2	2	2	2	1	0.25
Eukaryotic translation initiation factor 3 subunit A OS=Mus musculus GN=Eif3a PE=1 SV=5	P23116	162 kDa	0	0	0	3	2	2	2	2	1	0.25
Eukaryotic translation initiation factor 3 subunit C OS=Mus musculus GN=Eif3c PE=1 SV=1	Q8R1B4	106 kDa	0	0	0	3	2	2	2	2	1	0.25
Protein FAM60A OS=Mus musculus GN=Fam60a PE=2 SV=1	Q8C8M1	25 kDa	0	0	0	3	2	2	2	2	1	0.25
RNA-binding protein 8A OS=Mus musculus GN=RBM8 PE=1 SV=1	A0A023T672	20 kDa	0	0	0	3	2	2	2	2	1	0.25
Nuclear pore complex protein Nup50 OS=Mus musculus GN=Nup50 PE=1 SV=3	Q9JIH2	49 kDa	0	0	0	3	2	2	2	2	1	0.25
Protein Zfp512b OS=Mus musculus GN=Zfp512b PE=1 SV=1	B7ZCR6 (+1)	95 kDa	0	0	0	3	2	2	2	2	1	0.25
Ribonuclease P protein subunit p30 OS=Mus musculus GN=Rpp30 PE=1 SV=1	O88796	29 kDa	0	0	0	2	2	2	2	2	1	0.4
Double-stranded RNA-binding protein Staufen homolog 1 OS=Mus musculus GN=Stau1 PE=4 SV=1	A2A5S3	55 kDa	0	0	0	2	2	2	2	2	1	0.4
N-acylneuraminate cytidylyltransferase OS=Mus musculus GN=Cmas PE=1 SV=2	Q99KK2	48 kDa	0	0	0	3	2	2	2	2	1	0.25
DNA replication licensing factor MCM6 OS=Mus musculus GN=Mcm6 PE=1 SV=1	P97311 (+1)	93 kDa	0	0	0	2	2	2	2	2	1	0.4
Ubiquitin carboxyl-terminal hydrolase 10 OS=Mus musculus GN=Usp10 PE=1 SV=3	P52479	87 kDa	0	0	0	2	2	2	2	2	1	0.4
Lysine-specific demethylase 2B OS=Mus musculus GN=Kdm2b PE=4 SV=1	D3YVU4 (+3)	146 kDa	0	0	0	2	2	2	2	2	1	0.4
A-kinase anchor protein 8 OS=Mus musculus GN=Akap8 PE=1 SV=1	Q9DBR0	76 kDa	0	0	0	2	2	2	2	2	1	0.4
Dual specificity protein kinase TTK OS=Mus musculus GN=Ttk PE=1 SV=1	P35761 (+1)	96 kDa	0	0	0	2	2	2	2	2	1	0.4
rRNA-processing protein UTP23 homolog OS=Mus musculus GN=Utp23 PE=2 SV=1	Q9CX11	28 kDa	0	0	0	2	2	2	2	2	1	0.4
Vesicle-trafficking protein SEC22b OS=Mus musculus GN=Sec22b PE=1 SV=1	E9Q6R3 (+1)	19 kDa	0	0	0	2	2	2	2	2	1	0.4
Tight junction protein ZO-2 OS=Mus musculus GN=Tjp2 PE=1 SV=2	Q9Z0U1	131 kDa	0	0	0	2	2	2	2	2	1	0.4
Centromere protein T OS=Mus musculus GN=Cenpt PE=2 SV=2	Q3TJM4	56 kDa	0	0	0	2	2	2	2	2	1	0.4
Maternal embryonic leucine zipper kinase OS=Mus musculus GN=Melk PE=1 SV=2	Q61846	73 kDa	0	0	0	2	2	2	2	2	1	0.4
26S proteasome non-ATPase regulatory subunit 14 OS=Mus musculus GN=Psmd14 PE=1 SV=2	O35593	35 kDa	0	0	0	2	2	2	2	2	1	0.4
Symplekin OS=Mus musculus GN=Sympk PE=1 SV=1	F8WJD4 (+1)	143 kDa	0	0	0	2	2	2	2	2	1	0.4
Protein MCM10 homolog OS=Mus musculus GN=Mcm10 PE=1 SV=1	Q0VBD2	98 kDa	0	0	0	2	2	2	2	2	1	0.4
Signal recognition particle 14 kDa protein OS=Mus musculus GN=Srp14 PE=1 SV=1	P16254	13 kDa	0	0	0	2	2	2	2	2	1	0.4
Serine/arginine-rich-splicing factor 4 OS=Mus musculus GN=Srsf4 PE=1 SV=1	Q542V3 (+1)	56 kDa	0	0	0	3	2	2	2	2	1	0.25
Inosine-5'-monophosphate dehydrogenase 2 (Fragment) OS=Mus musculus GN=Impdh2 PE=1 SV=1	A0A0A6YY72 (+1)	34 kDa	0	0	0	2	2	2	2	2	1	0.4
Eukaryotic translation initiation factor 5B OS=Mus musculus GN=Eif5b PE=1 SV=2	Q05D44	138 kDa	0	0	0	2	2	2	2	2	1	0.4
Electron transfer flavoprotein subunit beta OS=Mus musculus GN=Etfb PE=1 SV=3	Q9DCW4	28 kDa	0	0	0	2	2	2	2	2	1	0.4
Structural maintenance of chromosomes protein 1A OS=Mus musculus GN=Smc1a PE=1 SV=4	Q9CU62	143 kDa	0	0	0	2	2	2	2	2	1	0.4
Formin-binding protein 4 OS=Mus musculus GN=Fnbp4 PE=1 SV=2	Q6ZQ03	111 kDa	0	0	0	2	2	2	2	2	1	0.4
Filamin-B OS=Mus musculus GN=Flnb PE=1 SV=3	Q80X90	278 kDa	0	0	0	3	2	2	2	2	1	0.25
Transcription elongation factor B polypeptide 2 OS=Mus musculus GN=Tceb2 PE=1 SV=1	P62869	13 kDa	0	0	0	2	2	2	2	2	1	0.4
Set1/Ash2 histone methyltransferase complex subunit ASH2 OS=Mus musculus GN=Ash2l PE=4 SV=1	E9PU93 (+1)	60 kDa	0	0	0	2	2	2	2	2	1	0.4
Heat shock 70 kDa protein 14 OS=Mus musculus GN=Hspa14 PE=1 SV=2	Q99M31	55 kDa	0	0	0	2	2	2	2	2	1	0.4
Lymphocyte-specific helicase OS=Mus musculus GN=Hells PE=1 SV=2	Q60848	95 kDa	9	5	9	13	13	6	10	8	0.9	0.46
Heterogeneous nuclear ribonucleoprotein A1 OS=Mus musculus GN=Hnrnpa1 PE=1 SV=2	P49312 (+1)	34 kDa	3	17	9	20	4	22	10	12	0.9	0.33
60S ribosomal protein L12 OS=Mus musculus GN=Rpl12 PE=1 SV=2	P35979	18 kDa	12	6	10	16	17	8	11	10	0.9	0.34
RNA-binding protein 14 OS=Mus musculus GN=Rbm14 PE=1 SV=1	Q8C2Q3	69 kDa	7	8	12	7	10	10	14	4	0.9	0.24
Blood vessel epicardial substance OS=Mus musculus GN=Pop1 PE=1 SV=1	Q8K205 (+1)	117 kDa	6	8	8	13	9	10	9	8	0.9	0.41
40S ribosomal protein S15a OS=Mus musculus GN=Rps15a PE=1 SV=2	P62245	15 kDa	5	5	6	8	7	6	7	5	0.9	0.38
Nuclease-sensitive element-binding protein 1 OS=Mus musculus GN=Ybx1 PE=1 SV=3	P62960	36 kDa	3	6	5	8	4	8	6	5	0.9	0.42
40S ribosomal protein S19 OS=Mus musculus GN=Rps19 PE=1 SV=3	Q9CZX8	16 kDa	5	3	4	9	7	4	5	5	0.9	0.53
Serine/threonine-protein phosphatase PP1-alpha catalytic subunit OS=Mus musculus GN=Ppp1ca PE=1 SV=1	P62137	38 kDa	0	8	0	15	2	10	2	9	0.9	0.51
39S ribosomal protein L46, mitochondrial OS=Mus musculus GN=Mrpl46 PE=1 SV=1	Q9EQI8	32 kDa	6	0	5	6	9	2	6	4	0.9	0.56
Serine/threonine-protein kinase PLK1 OS=Mus musculus GN=Plk1 PE=1 SV=2	Q07832 (+1)	68 kDa	4	0	3	6	6	2	3	4	0.9	0.45
S-phase kinase-associated protein 1 OS=Mus musculus GN=Skp1 PE=1 SV=3	Q9WTX5	19 kDa	0	4	4	3	2	5	5	2	0.9	0.62
Microtubule-actin cross-linking factor 1 OS=Mus musculus GN=Macf1 PE=1 SV=1	B1ARU4 (+3)	832 kDa	2	0	2	2	3	2	2	2	0.9	0.61
Transcription and mRNA export factor ENY2 OS=Mus musculus GN=Eny2 PE=1 SV=1	Q9JIX0	12 kDa	0	2	0	2	2	3	2	2	0.9	0.47
ATP-dependent zinc metalloprotease YME1L1 OS=Mus musculus GN=Yme1l1 PE=1 SV=1	O88967	80 kDa	3	0	3	3	4	2	3	2	0.9	0.56
Nucleolar protein 7 OS=Mus musculus GN=Nol7 PE=1 SV=1	Q9D7Z3	29 kDa	3	0	0	6	4	2	2	4	0.9	0.56
Bystin OS=Mus musculus GN=Bysl PE=1 SV=3	O54825	50 kDa	2	0	2	3	3	2	2	2	0.9	0.49
Cleavage and polyadenylation specificity factor subunit 5 OS=Mus musculus GN=Nudt21 PE=1 SV=1	Q9CQF3	26 kDa	0	4	4	0	2	5	5	2	0.9	0.33
THO complex subunit 5 homolog OS=Mus musculus GN=Thoc5 PE=1 SV=2	Q8BKT7	79 kDa	0	2	0	3	2	3	2	2	0.9	0.61
Zinc finger and SCAN domain-containing protein 10 (Fragment) OS=Mus musculus GN=Zscan10 PE=4 SV=1	E9PXB1 (+1)	79 kDa	0	2	0	2	2	3	2	2	0.9	0.47
Nucleolar and spindle-associated protein 1 OS=Mus musculus GN=Nusap1 PE=1 SV=1	Q9ERH4	49 kDa	0	2	0	2	2	3	2	2	0.9	0.47
DNA-directed RNA polymerases I and III subunit RPAC2 OS=Mus musculus GN=Polr1d PE=1 SV=1	Q9D1M1	14 kDa	0	2	0	0	2	3	2	2	0.9	0.14
Cleavage and polyadenylation-specificity factor subunit 6 OS=Mus musculus GN=Cpsf6 PE=1 SV=1	H3BJ30 (+2)	59 kDa	0	2	0	0	2	3	2	2	0.9	0.14
Trans-acting transcription factor 1 OS=Mus musculus GN=Sp1 PE=1 SV=1	G3X8Q0 (+1)	80 kDa	0	2	0	0	2	3	2	2	0.9	0.14
G2 and S phase-expressed protein 1 OS=Mus musculus GN=Gtse1 PE=1 SV=2	Q8R080	79 kDa	0	2	0	0	2	3	2	2	0.9	0.14
NTF2-related export protein 1 OS=Mus musculus GN=Nxt1 PE=1 SV=2	Q9QZV9	16 kDa	0	2	0	0	2	3	2	2	0.9	0.14
Cleavage and polyadenylation specificity factor subunit 3 OS=Mus musculus GN=Cpsf3 PE=1 SV=2	Q9QXK7	78 kDa	0	2	0	0	2	3	2	2	0.9	0.14
Putative RNA-binding protein Luc7-like 1 OS=Mus musculus GN=Luc7l PE=2 SV=2	Q9CYI4	44 kDa	0	2	0	0	2	3	2	2	0.9	0.14
Phospholipid hydroperoxide glutathione peroxidase, mitochondrial OS=Mus musculus GN=Gpx4 PE=1 SV=4	O70325 (+3)	22 kDa	0	2	0	0	2	3	2	2	0.9	0.14
Cytochrome b-c1 complex subunit 1, mitochondrial OS=Mus musculus GN=Uqcrc1 PE=1 SV=2	Q9CZ13	53 kDa	0	2	0	0	2	3	2	2	0.9	0.14
Heterogeneous nuclear ribonucleoprotein L (Fragment) OS=Mus musculus GN=Hnrnpl PE=1 SV=1	G5E924	67 kDa	11	18	12	32	16	23	14	19	0.8	0.34
40S ribosomal protein S3 OS=Mus musculus GN=Rps3 PE=1 SV=1	P62908	27 kDa	11	9	9	20	16	12	10	12	0.8	0.33
60S ribosomal protein L23 OS=Mus musculus GN=Rpl23 PE=1 SV=1	P62830	15 kDa	10	7	10	12	14	9	11	7	0.8	0.24
Probable ATP-dependent RNA helicase DDX52 OS=Mus musculus GN=Ddx52 PE=2 SV=2	Q8K301	67 kDa	11	4	7	14	16	5	8	8	0.8	0.33
DNA-directed RNA polymerase II subunit RPB1 OS=Mus musculus GN=Polr2a PE=1 SV=3	P08775	217 kDa	4	7	4	11	6	9	5	7	0.8	0.35
U4/U6 small nuclear ribonucleoprotein Prp3 OS=Mus musculus GN=Prpf3 PE=1 SV=1	Q922U1	77 kDa	7	6	8	9	10	8	9	5	0.8	0.29
60S ribosomal protein L10 (Fragment) OS=Mus musculus GN=Rpl10 PE=1 SV=1	I7HLV2 (+1)	23 kDa	4	5	5	6	6	6	6	4	0.8	0.29
Methionine aminopeptidase 1 OS=Mus musculus GN=Metap1 PE=2 SV=1	Q8BP48	43 kDa	0	6	4	6	2	8	5	4	0.8	0.57
78 kDa glucose-regulated protein OS=Mus musculus GN=Hspa5 PE=1 SV=3	P20029	72 kDa	5	8	7	11	7	10	8	7	0.8	0.34
40S ribosomal protein S5 OS=Mus musculus GN=Rps5 PE=1 SV=1	Q91V55	23 kDa	0	5	4	4	2	6	5	2	0.8	0.55

Aurora kinase B OS=Mus musculus GN=Aurkb PE=1 SV=2	O70126	39 kDa	4	0	0	7	6	2	2	4	0.8	0.62
60S ribosomal protein L22 OS=Mus musculus GN=Rpl22 PE=1 SV=2	P67984	15 kDa	4	0	0	7	6	2	2	4	0.8	0.62
Zinc finger RNA-binding protein OS=Mus musculus GN=Zfr PE=1 SV=2	O88532	117 kDa	0	4	3	0	2	5	3	2	0.8	0.23
Beta-catenin-like protein 1 OS=Mus musculus GN=Ctnnbl1 PE=1 SV=1	Q9CWL8	65 kDa	2	0	0	2	3	2	2	2	0.8	0.47
Prohibitin-2 (Fragment) OS=Mus musculus GN=Phb2 PE=1 SV=1	F6QPR1	18 kDa	2	0	0	0	3	2	2	2	0.8	0.14
Glycine dehydrogenase (decarboxylating), mitochondrial OS=Mus musculus GN=Gldc PE=1 SV=1	Q91W43	113 kDa	2	0	0	0	3	2	2	2	0.8	0.14
RNA demethylase ALKBH5 OS=Mus musculus GN=Alkbh5 PE=1 SV=2	Q3TSG4	44 kDa	2	0	0	0	3	2	2	2	0.8	0.14
Cytochrome b-c1 complex subunit Rieske, mitochondrial OS=Mus musculus GN=Uqcrfs1 PE=1 SV=1	Q9CR68	29 kDa	2	0	0	0	3	2	2	2	0.8	0.14
RNA pseudouridylate synthase domain-containing protein 3 OS=Mus musculus GN=Rpusd3 PE=2 SV=1	Q14AI6	38 kDa	2	0	0	0	3	2	2	2	0.8	0.14
Catechol O-methyltransferase domain-containing protein 1 OS=Mus musculus GN=Comtd1 PE=1 SV=1	Q8BIG7	29 kDa	2	0	0	0	3	2	2	2	0.8	0.14
Dedicator of cytokinesis protein 6 OS=Mus musculus GN=Dock6 PE=1 SV=1	E9QPN7 (+1)	237 kDa	2	0	0	0	3	2	2	2	0.8	0.14
Guanine nucleotide-binding protein G(s) subunit alpha isoforms short OS=Mus musculus GN=Gnas PE=1 SV=1	P63094 (+1)	46 kDa	2	0	0	0	3	2	2	2	0.8	0.14
ATP-dependent RNA helicase A OS=Mus musculus GN=Dhx9 PE=1 SV=1	E9QNN1	150 kDa	33	23	20	47	47	30	23	28	0.7	0.029
40S ribosomal protein S3a OS=Mus musculus GN=Rps3a PE=1 SV=3	P97351	30 kDa	11	16	16	11	16	21	18	7	0.7	0.032
Eukaryotic initiation factor 4A-III OS=Mus musculus GN=Eif4a3 PE=1 SV=3	Q91VC3	47 kDa	13	8	10	14	18	10	11	8	0.7	0.11
Recombining-binding protein suppressor of hairless OS=Mus musculus GN=Rbpj PE=1 SV=1	E9Q7W0 (+1)	54 kDa	10	14	12	15	14	18	14	9	0.7	0.086
TAR DNA-binding protein 43 OS=Mus musculus GN=Tardbp PE=1 SV=1	Q921F2	45 kDa	8	9	7	13	11	12	8	8	0.7	0.16
ATP synthase subunit beta, mitochondrial OS=Mus musculus GN=Atp5b PE=1 SV=2	P56480	56 kDa	7	10	9	9	10	13	10	5	0.7	0.1
KH domain-containing, RNA-binding, signal transduction-associated protein 1 OS=Mus musculus GN=Khdrbs1 PE=1 SV=2	Q60749	48 kDa	4	10	9	6	6	13	10	4	0.7	0.14
40S ribosomal protein S12 OS=Mus musculus GN=Rps12 PE=1 SV=1	Q6ZWZ6	15 kDa	5	7	4	10	7	9	5	6	0.7	0.21
DNA (cytosine-5)-methyltransferase 3-like OS=Mus musculus GN=Dnmt3l PE=1 SV=1	Q9CWR8	48 kDa	3	8	3	11	4	10	3	7	0.7	0.29
Plasminogen activator inhibitor 1 RNA-binding protein OS=Mus musculus GN=Serbp1 PE=1 SV=2	Q9CY58	45 kDa	0	8	4	7	2	10	5	4	0.7	0.4
Sal-like protein 4 OS=Mus musculus GN=Sall4 PE=1 SV=2	Q8BX22	113 kDa	0	6	2	7	2	8	2	4	0.7	0.5
Apoptosis-enhancing nuclease OS=Mus musculus GN=Aen PE=2 SV=1	Q9CZI9	37 kDa	4	2	3	4	6	3	3	2	0.7	0.33
Luc7-like protein 3 OS=Mus musculus GN=Luc7l3 PE=1 SV=1	Q5SUF2	51 kDa	2	3	0	5	3	4	2	3	0.7	0.29
Dolichyl-diphosphooligosaccharide--protein glycosyltransferase subunit 1 OS=Mus musculus GN=Rpn1 PE=1 SV=1	Q91YQ5	69 kDa	0	3	0	4	2	4	2	2	0.7	0.51
MCG11048, isoform CRA_c OS=Mus musculus GN=Tecr PE=1 SV=1	G3UWE1 (+2)	34 kDa	0	3	0	3	2	4	2	2	0.7	0.39
Spliceosome RNA helicase Ddx39b OS=Mus musculus GN=Ddx39b PE=1 SV=1	Q9Z1N5	49 kDa	0	3	0	3	2	4	2	2	0.7	0.39
Actin-binding protein anillin OS=Mus musculus GN=Anln PE=1 SV=2	Q8K298	123 kDa	2	2	0	0	3	3	2	2	0.7	0.018
Serum response factor-binding protein 1 OS=Mus musculus GN=Srfbp1 PE=1 SV=1	Q9CZ91	49 kDa	0	3	0	3	2	4	2	2	0.7	0.39
MCG130458 OS=Mus musculus GN=Fubp3 PE=4 SV=1	A2AJ72 (+1)	61 kDa	0	3	0	3	2	4	2	2	0.7	0.39
Serine/threonine-protein phosphatase PP1-beta catalytic subunit OS=Mus musculus GN=Ppp1cb PE=1 SV=3	P62141	37 kDa	11	0	9	0	16	2	10	2	0.7	0.075
Krueppel-like factor 4 OS=Mus musculus GN=Klf4 PE=1 SV=3	Q60793	52 kDa	0	3	0	0	2	4	2	2	0.7	0.05
Protein Zfp534 OS=Mus musculus GN=Zfp534 PE=4 SV=2	A2A7A1	78 kDa	0	3	0	0	2	4	2	2	0.7	0.05
Nucleolar protein 12 OS=Mus musculus GN=Nol12 PE=4 SV=1	B7ZBX7 (+1)	20 kDa	0	3	0	0	2	4	2	2	0.7	0.05
Up-regulated during skeletal muscle growth protein 5 OS=Mus musculus GN=Usmg5 PE=1 SV=1	Q78IK2	6 kDa	0	3	0	0	2	4	2	2	0.7	0.05
Protein transport protein Sec61 subunit beta OS=Mus musculus GN=Sec61b PE=1 SV=3	Q9CQS8	10 kDa	0	3	0	0	2	4	2	2	0.7	0.05
Cleavage and polyadenylation specific factor 4 isoform 1 OS=Mus musculus GN=Cpsf4 PE=1 SV=1	B2LVG5 (+2)	27 kDa	0	3	0	0	2	4	2	2	0.7	0.05
Zinc finger CCCH domain-containing protein 4 OS=Mus musculus GN=Zc3h4 PE=1 SV=1	E9Q8K8 (+1)	136 kDa	0	3	0	0	2	4	2	2	0.7	0.05
Small nuclear ribonucleoprotein F OS=Mus musculus GN=Snrpf PE=1 SV=1	P62307	10 kDa	0	3	0	0	2	4	2	2	0.7	0.05
60S ribosomal protein L36a OS=Mus musculus GN=Rpl36a PE=1 SV=2	P83882	12 kDa	0	3	0	0	2	4	2	2	0.7	0.05
DnaJ homolog subfamily C member 8 OS=Mus musculus GN=Dnajc8 PE=2 SV=2	Q6NZB0	30 kDa	0	3	0	0	2	4	2	2	0.7	0.05
Protein RTF2 homolog OS=Mus musculus GN=Rtfdc1 PE=1 SV=1	Q99K95	34 kDa	0	3	0	0	2	4	2	2	0.7	0.05
Signal recognition particle subunit SRP68 OS=Mus musculus GN=Srp68 PE=1 SV=1	A2AAN2 (+1)	66 kDa	0	3	0	0	2	4	2	2	0.7	0.05
BUD13 homolog OS=Mus musculus GN=Bud13 PE=1 SV=1	Q8R149	72 kDa	0	3	0	0	2	4	2	2	0.7	0.05
Keratin, type I cytoskeletal 10 OS=Mus musculus GN=Krt10 PE=3 SV=1	A2A513 (+1)	57 kDa	0	3	0	0	2	4	2	2	0.7	0.05
Nardilysin OS=Mus musculus GN=Nrd1 PE=1 SV=1	A2A9Q2 (+2)	141 kDa	0	3	0	0	2	4	2	2	0.7	0.05
MCG13402, isoform CRA_a OS=Mus musculus GN=Ptbp1 PE=1 SV=1	Q8BGJ5 (+1)	57 kDa	32	36	23	49	46	47	26	30	0.6	0.0028
RRP12-like protein OS=Mus musculus GN=Rrp12 PE=1 SV=1	Q6P5B0	143 kDa	22	23	17	26	31	30	20	16	0.6	0.0042
Lamina-associated polypeptide 2, isoforms beta/delta/epsilon/gamma OS=Mus musculus GN=Tmpo PE=1 SV=4	Q61029	50 kDa	9	15	9	14	13	19	10	8	0.6	0.032
Heterogeneous nuclear ribonucleoprotein H OS=Mus musculus GN=Hnrnph1 PE=1 SV=3	O35737 (+1)	49 kDa	11	17	12	17	16	22	14	10	0.6	0.038
Developmental pluripotency-associated protein 2 OS=Mus musculus GN=Dppa2 PE=1 SV=1	Q9CWH0	34 kDa	10	7	6	10	14	9	7	6	0.6	0.06
Protein Red OS=Mus musculus GN=Ik PE=1 SV=2	Q9Z1M8	66 kDa	5	10	7	8	7	13	8	5	0.6	0.097
Protein lin-28 homolog A OS=Mus musculus GN=Lin28a PE=1 SV=1	Q8K3Y3	23 kDa	4	10	6	8	6	13	7	5	0.6	0.11
5'-3' exoribonuclease 2 OS=Mus musculus GN=Xrn2 PE=1 SV=1	Q9DBR1	109 kDa	7	5	4	8	10	6	5	5	0.6	0.13
40S ribosomal protein S11 OS=Mus musculus GN=Rps11 PE=1 SV=3	P62281	18 kDa	7	6	4	10	10	8	5	6	0.6	0.15
Nuclear cap-binding protein subunit 1 OS=Mus musculus GN=Ncbp1 PE=1 SV=2	Q3UYV9	92 kDa	6	4	4	7	9	5	5	4	0.6	0.21
Protein regulator of cytokinesis 1 OS=Mus musculus GN=Prc1 PE=1 SV=1	G3UW86 (+2)	71 kDa	0	6	3	3	2	8	3	2	0.6	0.25
40S ribosomal protein S10 OS=Mus musculus GN=Rps10 PE=1 SV=1	P63325	19 kDa	4	3	3	4	6	4	3	2	0.6	0.23
Cyclin-dependent kinase 1 OS=Mus musculus GN=Cdk1 PE=1 SV=3	P11440	34 kDa	3	4	0	6	4	5	2	4	0.6	0.16
60S ribosomal protein L27 OS=Mus musculus GN=Rpl27 PE=1 SV=2	P61358	16 kDa	0	6	0	7	2	8	2	4	0.6	0.33
DnaJ homolog subfamily A member 3, mitochondrial OS=Mus musculus GN=Dnaja3 PE=1 SV=1	Q99M87	52 kDa	3	0	0	3	4	2	2	2	0.6	0.39
Ran GTPase-activating protein 1 OS=Mus musculus GN=Rangap1 PE=1 SV=2	P46061	64 kDa	4	0	0	4	6	2	2	2	0.6	0.33
SWI/SNF complex subunit SMARCC1 OS=Mus musculus GN=Smarcc1 PE=1 SV=2	P97496	123 kDa	0	4	0	4	2	5	2	2	0.6	0.33
Putative methyltransferase C9orf114 homolog OS=Mus musculus GN=D2Wsu81e PE=1 SV=1	Q3UHX9	43 kDa	0	4	0	4	2	5	2	2	0.6	0.33
High mobility group protein HMGI-C OS=Mus musculus GN=Hmga2 PE=1 SV=1	P52927	12 kDa	0	4	0	2	2	5	2	2	0.6	0.14
Origin recognition complex subunit 2 OS=Mus musculus GN=Orc2 PE=1 SV=1	Q60862	66 kDa	4	0	0	4	6	2	2	2	0.6	0.33
CGG triplet repeat-binding protein 1 OS=Mus musculus GN=Cggbp1 PE=1 SV=1	Q8BHG9	19 kDa	3	0	0	2	4	2	2	2	0.6	0.26
Mitochondrial import inner membrane translocase subunit TIM50 OS=Mus musculus GN=Timm50 PE=1 SV=1	Q9D880	40 kDa	2	3	0	0	3	4	2	2	0.6	0.0068
Peptidyl-prolyl cis-trans isomerase-like 2 OS=Mus musculus GN=Ppil2 PE=1 SV=2	Q9D787	59 kDa	2	3	0	0	3	4	2	2	0.6	0.0068
Pre-mRNA-splicing factor ISY1 homolog OS=Mus musculus GN=Isy1 PE=1 SV=2	Q69ZQ2	33 kDa	0	4	0	1	2	5	2	2	0.6	0.065
Proliferating cell nuclear antigen OS=Mus musculus GN=Pcna PE=1 SV=2	P17918	29 kDa	3	0	0	3	4	2	2	2	0.6	0.39
Dolichyl-diphosphooligosaccharide--protein glycosyltransferase subunit 2 OS=Mus musculus GN=Rpn2 PE=1 SV=1	A2ACG7 (+1)	68 kDa	3	0	0	3	4	2	2	2	0.6	0.39
U4/U6 small nuclear ribonucleoprotein Prp31 OS=Mus musculus GN=Prpf31 PE=1 SV=3	Q8CCF0	55 kDa	3	0	0	2	4	2	2	2	0.6	0.26
Spliceosome-associated protein CWC15 homolog OS=Mus musculus GN=Cwc15 PE=1 SV=1	Q9JHS9	27 kDa	3	0	0	2	4	2	2	2	0.6	0.26
Polypyrimidine tract-binding protein 3 OS=Mus musculus GN=Ptbp3 PE=4 SV=1	G3UXA6 (+2)	60 kDa	0	4	0	0	2	5	2	2	0.6	0.018
Cell division cycle protein 20 homolog OS=Mus musculus GN=Cdc20 PE=1 SV=2	Q9JJ66	55 kDa	0	4	0	0	2	5	2	2	0.6	0.018
Probable JmjC domain-containing histone demethylation protein 2C OS=Mus musculus GN=Jmjd1c PE=1 SV=1	A0A0A0MQ98 (+3)	282 kDa	3	0	0	0	4	2	2	2	0.6	0.05
U1 small nuclear ribonucleoprotein C OS=Mus musculus GN=Snrpc PE=2 SV=1	Q62241	17 kDa	0	4	0	0	2	5	2	2	0.6	0.018
Eukaryotic translation initiation factor 4B OS=Mus musculus GN=Eif4b PE=1 SV=1	Q8BGD9	69 kDa	0	4	0	0	2	5	2	2	0.6	0.018
UPF0609 protein C4orf27 homolog OS=Mus musculus PE=1 SV=1	Q8CFE2	39 kDa	3	0	0	0	4	2	2	2	0.6	0.05
tRNA-dihydrouridine(47) synthase [NAD(P)(+)]-like OS=Mus musculus GN=Dus3l PE=2 SV=1	Q91XI1 (+1)	71 kDa	3	0	0	0	4	2	2	2	0.6	0.05
Pre-mRNA-processing factor 17 OS=Mus musculus GN=Cdc40 PE=2 SV=1	Q9DC48	65 kDa	0	4	0	0	2	5	2	2	0.6	0.018
TAF2 RNA polymerase II, TATA box binding protein (TBP)-associated factor OS=Mus musculus GN=Taf2 PE=1 SV=1	B9EJX5 (+1)	137 kDa	3	0	0	0	4	2	2	2	0.6	0.05
ATP synthase-coupling factor 6, mitochondrial OS=Mus musculus GN=Atp5j PE=1 SV=1	P97450	12 kDa	3	0	0	0	4	2	2	2	0.6	0.05
Pyruvate dehydrogenase E1 component subunit alpha, somatic form, mitochondrial OS=Mus musculus GN=Pdha1 PE=1 SV=1	P35486 (+1)	43 kDa	3	0	0	0	4	2	2	2	0.6	0.05
Protein arginine N-methyltransferase 1 OS=Mus musculus GN=Prmt1 PE=1 SV=1	Q3UIG8 (+1)	41 kDa	3	0	0	0	4	2	2	2	0.6	0.05
Mediator of RNA polymerase II transcription subunit 19 OS=Mus musculus GN=Med19 PE=1 SV=1	Q8C1S0	26 kDa	3	0	0	0	4	2	2	2	0.6	0.05
WW domain-binding protein 11 OS=Mus musculus GN=Wbp11 PE=1 SV=2	Q923D5	70 kDa	3	0	0	0	4	2	2	2	0.6	0.05
Protein Srsf11 OS=Mus musculus GN=Srsf11 PE=1 SV=1	E9Q6E5 (+2)	57 kDa	3	0	0	0	4	2	2	2	0.6	0.05
CWF19-like protein 1 OS=Mus musculus GN=Cwf19l1 PE=1 SV=2	Q8CI33	60 kDa	3	0	0	0	4	2	2	2	0.6	0.05
Probable ATP-dependent RNA helicase DDX5 OS=Mus musculus GN=Ddx5 PE=1 SV=2	Q61656 (+1)	69 kDa	38	52	31	47	54	68	36	28	0.5	< 0.00010
Heat shock cognate 71 kDa protein OS=Mus musculus GN=Hspa8 PE=1 SV=1	P63017	71 kDa	25	32	19	32	36	42	22	19	0.5	0.00051
Cell division cycle 5-like protein OS=Mus musculus GN=Cdc5l PE=1 SV=2	Q6A068	92 kDa	25	24	14	25	36	31	16	15	0.5	0.00024
rRNA 2'-O-methyltransferase fibrillarin OS=Mus musculus GN=Fbl PE=1 SV=2	P35550	34 kDa	22	23	8	34	31	30	9	21	0.5	0.0031
Histone H3 OS=Mus musculus GN=Hist2h3b PE=1 SV=1	B9EI85 (+1)	15 kDa	9	4	3	8	13	5	3	5	0.5	0.063
40S ribosomal protein S4, X isoform OS=Mus musculus GN=Rps4x PE=1 SV=2	P62702	30 kDa	19	10	8	16	27	13	9	10	0.5	0.0059
Nuclear RNA export factor 1 OS=Mus musculus GN=Nxf1 PE=1 SV=3	Q99JX7	70 kDa	10	17	5	18	14	22	6	11	0.5	0.0097
DEAD (Asp-Glu-Ala-Asp) box polypeptide 17, isoform CRA_a OS=Mus musculus GN=Ddx17 PE=1 SV=1	Q3U741 (+1)	73 kDa	24	34	18	28	34	44	21	17	0.5	< 0.00010
U4/U6.U5 tri-snRNP-associated protein 1 OS=Mus musculus GN=Sart1 PE=1 SV=1	Q9Z315	91 kDa	10	18	9	11	14	23	10	7	0.5	0.002
E3 SUMO-protein ligase RanBP2 OS=Mus musculus GN=Ranbp2 PE=1 SV=2	Q9ERU9	341 kDa	5	10	7	4	7	13	8	2	0.5	0.024
ELAV-like protein 1 OS=Mus musculus GN=Elavl1 PE=1 SV=2	P70372	36 kDa	7	8	3	10	10	10	3	6	0.5	0.052
Splicing factor, proline- and glutamine-rich OS=Mus musculus GN=Sfpq PE=1 SV=1	Q8VIJ6	75 kDa	9	6	3	11	13	8	3	7	0.5	0.072

40S ribosomal protein S13 OS=Mus musculus GN=Rps13 PE=1 SV=2	P62301	17 kDa	7	6	3	8	10	8	3	5	0.5	0.063
L-threonine 3-dehydrogenase, mitochondrial OS=Mus musculus GN=Tdh PE=2 SV=1	Q8K3F7	41 kDa	14	5	7	7	20	6	8	4	0.5	0.012
ATP synthase subunit gamma OS=Mus musculus GN=Atp5c1 PE=1 SV=1	Q8C2Q8 (+1)	30 kDa	6	7	5	5	9	9	6	3	0.5	0.043
Non-POU domain-containing octamer-binding protein OS=Mus musculus GN=Nono PE=1 SV=3	Q99K48	55 kDa	6	7	3	9	9	9	3	5	0.5	0.087
40S ribosomal protein S14 OS=Mus musculus GN=Rps14 PE=1 SV=3	P62264	16 kDa	3	10	4	6	4	13	5	4	0.5	0.043
General transcription factor II-I OS=Mus musculus GN=Gtf2i PE=1 SV=1	G3UYD0 (+2)	103 kDa	6	3	1	6	9	4	2	4	0.5	0.09
Transcription elongation factor A protein 3 OS=Mus musculus GN=Tcea3 PE=1 SV=3	P23881	39 kDa	3	9	5	3	4	12	6	2	0.5	0.03
Kinesin-like protein KIF2C OS=Mus musculus GN=Kif2c PE=1 SV=1	Q922S8	81 kDa	6	4	4	4	9	5	5	2	0.5	0.082
Histone deacetylase complex subunit SAP18 OS=Mus musculus GN=Sap18 PE=1 SV=1	O55128 (+1)	18 kDa	0	5	1	3	2	6	2	2	0.5	0.2
Voltage-dependent anion-selective channel protein 2 (Fragment) OS=Mus musculus GN=Vdac2 PE=1 SV=1	G3UX26 (+1)	30 kDa	3	7	4	3	4	9	5	2	0.5	0.054
Peroxiredoxin-1 OS=Mus musculus GN=Prdx1 PE=1 SV=1	P35700	22 kDa	4	5	3	4	6	6	3	2	0.5	0.09
Ribosomal protein L15 OS=Mus musculus GN=Gm10020 PE=3 SV=1	E9QAZ2 (+1)	24 kDa	4	1	0	2	6	2	2	2	0.5	0.069
60S ribosomal protein L11 OS=Mus musculus GN=Rpl11 PE=1 SV=4	Q9CXW4	20 kDa	0	6	0	5	2	8	2	3	0.5	0.18
Far upstream element-binding protein 2 OS=Mus musculus GN=Khsrp PE=1 SV=2	Q3U0V1	77 kDa	4	4	3	0	6	5	3	2	0.5	0.017
THO complex subunit 2 OS=Mus musculus GN=Thoc2 PE=1 SV=1	B1AZI6	183 kDa	4	2	0	4	6	3	2	2	0.5	0.12
39S ribosomal protein L14, mitochondrial OS=Mus musculus GN=Mrpl14 PE=1 SV=1	Q9D1I6	16 kDa	3	3	0	0	4	4	2	2	0.5	0.0025
DnaJ homolog subfamily A member 1 OS=Mus musculus GN=Dnaja1 PE=1 SV=1	P63037	45 kDa	2	4	0	3	3	5	2	2	0.5	0.068
Zinc finger protein 281 OS=Mus musculus GN=Znf281 PE=1 SV=1	Q99LI5	97 kDa	0	5	0	3	2	6	2	2	0.5	0.13
Protein Nup153 OS=Mus musculus GN=Nup153 PE=1 SV=1	E9Q3G8	152 kDa	3	3	0	3	4	4	2	2	0.5	0.068
Glycerol-3-phosphate dehydrogenase OS=Mus musculus GN=Gpd2 PE=1 SV=1	A2AQR0 (+1)	83 kDa	5	0	0	4	7	2	2	2	0.5	0.2
40S ribosomal protein S23 OS=Mus musculus GN=Rps23 PE=1 SV=3	P62267	16 kDa	0	6	0	4	2	8	2	2	0.5	0.12
Tricarboxylate transport protein, mitochondrial OS=Mus musculus GN=Slc25a1 PE=1 SV=1	Q8JZU2	34 kDa	3	3	0	0	4	4	2	2	0.5	0.0025
Polycomb protein EED OS=Mus musculus GN=Eed PE=1 SV=1	Q921E6	50 kDa	0	5	0	4	2	6	2	2	0.5	0.2
DNA-directed RNA polymerases I, II, and III subunit RPABC1 OS=Mus musculus GN=Polr2e PE=2 SV=1	Q80UW8	25 kDa	0	5	0	2	2	6	2	2	0.5	0.069
Multifunctional protein ADE2 OS=Mus musculus GN=Paics PE=1 SV=4	Q9DCL9	47 kDa	4	0	0	2	6	2	2	2	0.5	0.14
Nuclear pore complex protein Nup160 OS=Mus musculus GN=Nup160 PE=1 SV=2	Q9Z0W3	158 kDa	4	0	0	2	6	2	2	2	0.5	0.14
Small nuclear ribonucleoprotein G OS=Mus musculus GN=Snrpg PE=1 SV=1	P62309	8 kDa	4	1	0	0	6	2	2	2	0.5	0.0068
GTP-binding nuclear protein Ran OS=Mus musculus GN=Ran PE=1 SV=3	P62827	24 kDa	3	3	0	0	4	4	2	2	0.5	0.0025
Keratin, type II cytoskeletal 1 OS=Mus musculus GN=Krt1 PE=1 SV=4	P04104	66 kDa	0	5	0	0	2	6	2	2	0.5	0.0068
Nucleoporin SEH1 OS=Mus musculus GN=Seh1l PE=2 SV=1	Q8R2U0	40 kDa	0	5	0	0	2	6	2	2	0.5	0.0068
Krueppel-like factor 5 OS=Mus musculus GN=Klf5 PE=2 SV=2	Q9Z0Z7	50 kDa	0	5	0	0	2	6	2	2	0.5	0.0068
Small nuclear ribonucleoprotein E OS=Mus musculus GN=Snrpe PE=1 SV=1	P62305	11 kDa	0	5	0	0	2	6	2	2	0.5	0.0068
Mitochondrial 2-oxoglutarate/malate carrier protein OS=Mus musculus GN=Slc25a11 PE=1 SV=1	Q5SX53 (+1)	34 kDa	4	0	0	0	6	2	2	2	0.5	0.018
Protein CMSS1 OS=Mus musculus GN=Cmss1 PE=2 SV=1	Q9CZT6	32 kDa	4	0	0	0	6	2	2	2	0.5	0.018
Destrin OS=Mus musculus GN=Dstn PE=1 SV=3	Q9R0P5 (+1)	19 kDa	4	0	0	0	6	2	2	2	0.5	0.018
Expressed sequence AI987944 OS=Mus musculus GN=AI987944 PE=2 SV=1	Q7TPX5	48 kDa	0	4	0	0	2	6	2	2	0.5	0.0068
ATP-dependent RNA helicase DDX39A OS=Mus musculus GN=Ddx39a PE=1 SV=1	Q8VDW0	49 kDa	4	0	0	0	6	2	2	2	0.5	0.018
116 kDa U5 small nuclear ribonucleoprotein component OS=Mus musculus GN=Eftud2 PE=2 SV=1	O08810	109 kDa	51	68	31	47	73	88	36	28	0.4	< 0.00010
Poly(U)-binding-splicing factor PUF60 OS=Mus musculus GN=Puf60 PE=1 SV=2	Q3UEB3	60 kDa	47	55	31	30	67	71	36	18	0.4	< 0.00010
Pre-mRNA-processing factor 19 OS=Mus musculus GN=Prpf19 PE=1 SV=1	Q99KP6	55 kDa	33	33	16	26	47	43	18	16	0.4	< 0.00010
ATP-dependent RNA helicase DDX3X OS=Mus musculus GN=Ddx3x PE=1 SV=3	Q62167	73 kDa	35	43	21	30	50	56	24	18	0.4	< 0.00010
WD40 repeat-containing protein SMU1 OS=Mus musculus GN=Smu1 PE=2 SV=2	Q3UKJ7	58 kDa	26	31	17	22	37	40	20	13	0.4	< 0.00010
Heterogeneous nuclear ribonucleoprotein A3 OS=Mus musculus GN=Hnrnpa3 PE=1 SV=1	A2AL12 (+1)	34 kDa	15	23	9	16	21	30	10	10	0.4	0.00013
Elongation factor 1-alpha 1 OS=Mus musculus GN=Eef1a1 PE=1 SV=3	P10126	50 kDa	16	19	10	12	23	25	11	7	0.4	0.00014
ATP synthase subunit alpha, mitochondrial OS=Mus musculus GN=Atp5a1 PE=1 SV=1	Q03265	60 kDa	20	13	10	13	28	17	11	8	0.4	0.00062
Microfibrillar-associated protein 1 OS=Mus musculus GN=Mfap1 PE=1 SV=1	Q9CQU1	52 kDa	12	25	13	11	17	32	15	7	0.4	0.00013
Targeting protein for Xklp2 OS=Mus musculus GN=Tpx2 PE=1 SV=1	A2APB8	86 kDa	7	23	5	17	10	30	6	10	0.4	0.0017
Kinesin-like protein KIF22 OS=Mus musculus GN=Kif22 PE=2 SV=2	Q3V300	73 kDa	17	14	5	17	24	18	6	10	0.4	0.0011
DNA helicase OS=Mus musculus GN=Mcm5 PE=1 SV=1	Q52KC3	82 kDa	9	15	6	8	13	19	7	5	0.4	0.00087
40S ribosomal protein S7 OS=Mus musculus GN=Rps7 PE=2 SV=1	P62082	22 kDa	10	12	4	12	14	16	5	7	0.4	0.0066
Pre-mRNA-splicing factor SPF27 OS=Mus musculus GN=Bcas2 PE=1 SV=1	Q9D287	26 kDa	11	11	3	12	16	14	3	7	0.4	0.0042
DNA replication licensing factor MCM3 OS=Mus musculus GN=Mcm3 PE=1 SV=2	P25206	92 kDa	10	11	6	9	14	14	7	5	0.4	0.007
Enhancer of rudimentary homolog OS=Mus musculus GN=Erh PE=1 SV=1	G3UW85	14 kDa	5	12	6	4	7	16	7	2	0.4	0.0051
Small nuclear ribonucleoprotein Sm D3 OS=Mus musculus GN=Snrpd3 PE=1 SV=1	P62320	14 kDa	6	9	5	5	9	12	6	3	0.4	0.015
Mitotic checkpoint protein BUB3 OS=Mus musculus GN=Bub3 PE=1 SV=2	Q9WVA3	37 kDa	5	9	4	5	7	12	5	3	0.4	0.016
Succinate dehydrogenase [ubiquinone] flavoprotein subunit, mitochondrial OS=Mus musculus GN=Sdha PE=1 SV=1	Q8K2B3	73 kDa	9	5	5	2	13	6	6	2	0.4	0.0053
60S ribosomal protein L7-like 1 OS=Mus musculus GN=Rpl7l1 PE=1 SV=1	Q9D8M4	29 kDa	10	4	0	8	14	5	2	5	0.4	0.0096
Signal recognition particle subunit SRP72 OS=Mus musculus GN=Srp72 PE=1 SV=1	F8VQC1	75 kDa	8	4	3	6	11	5	3	4	0.4	0.046
THO complex subunit 4 OS=Mus musculus GN=Alyref PE=1 SV=3	O08583	27 kDa	4	7	0	6	6	9	2	4	0.4	0.018
Cyclin-dependent kinase 11B OS=Mus musculus GN=Cdk11b PE=1 SV=2	P24788	92 kDa	4	7	0	6	6	9	2	4	0.4	0.018
Survival of motor neuron-related-splicing factor 30 OS=Mus musculus GN=Smndc1 PE=1 SV=1	Q8BGT7	27 kDa	6	6	0	7	9	8	2	4	0.4	0.018
ATP-dependent RNA helicase DHX8 OS=Mus musculus GN=Dhx8 PE=2 SV=1	A2A4P0	143 kDa	6	7	0	7	9	9	2	4	0.4	0.0097
Splicing factor 3B subunit 4 OS=Mus musculus GN=Sf3b4 PE=1 SV=1	Q8QZY9	44 kDa	0	10	0	7	2	13	2	4	0.4	0.054
Telomeric repeat-binding factor 1 OS=Mus musculus GN=Terf1 PE=1 SV=1	P70371	48 kDa	4	4	0	4	6	5	2	2	0.4	0.035
Transcription elongation factor, mitochondrial OS=Mus musculus GN=Tefm PE=1 SV=1	Q5SSK3	42 kDa	4	5	0	5	6	6	2	3	0.4	0.034
pre-mRNA 3' end processing protein WDR33 OS=Mus musculus GN=Wdr33 PE=1 SV=1	Q8K4P0	145 kDa	3	5	0	1	4	6	2	2	0.4	0.002
Cleavage and polyadenylation specificity factor subunit 2 OS=Mus musculus GN=Cpsf2 PE=1 SV=1	O35218	88 kDa	0	7	0	3	2	9	2	2	0.4	0.035
Aurora kinase A OS=Mus musculus GN=Aurka PE=1 SV=1	P97477	45 kDa	2	5	0	0	3	6	2	2	0.4	0.00092
40S ribosomal protein S26 OS=Mus musculus GN=Rps26 PE=1 SV=3	P62855 (+1)	13 kDa	5	3	0	0	7	4	2	2	0.4	0.00034
LIM domain and actin-binding protein 1 OS=Mus musculus GN=Lima1 PE=1 SV=3	Q9ERG0	84 kDa	4	3	0	0	6	4	2	2	0.4	0.00092
Coronin-1C OS=Mus musculus GN=Coro1c PE=1 SV=2	Q9WUM4	53 kDa	1	6	0	0	2	8	2	2	0.4	0.00092
LanC-like protein 2 (Fragment) OS=Mus musculus GN=Lancl2 PE=1 SV=1	F6RJV6 (+1)	50 kDa	4	3	0	0	6	4	2	2	0.4	0.00092
BUB3-interacting and GLEBS motif-containing protein ZNF207 OS=Mus musculus GN=Znf207 PE=2 SV=1	Q9JMD0	53 kDa	2	5	0	0	3	6	2	2	0.4	0.00092
40S ribosomal protein S15 OS=Mus musculus GN=Rps15 PE=1 SV=2	P62843	17 kDa	4	3	0	0	6	4	2	2	0.4	0.00092
60S ribosomal protein L22-like 1 OS=Mus musculus GN=Rpl22l1 PE=1 SV=1	Q9D7S7	14 kDa	0	7	0	0	2	9	2	2	0.4	0.00092
Glutathione S-transferase Mu 2 OS=Mus musculus GN=Gstm2 PE=1 SV=2	P15626 (+1)	26 kDa	0	6	0	0	2	8	2	2	0.4	0.0025
40S ribosomal protein S24 OS=Mus musculus GN=Rps24 PE=1 SV=1	P62849	15 kDa	0	6	0	0	2	8	2	2	0.4	0.0025
40S ribosomal protein S27-like OS=Mus musculus GN=Rps27l PE=3 SV=3	Q6ZWY3	9 kDa	0	6	0	0	2	8	2	2	0.4	0.0025
Zinc finger matrin-type protein 5 OS=Mus musculus GN=Zmat5 PE=2 SV=1	Q9CQR5	20 kDa	0	6	0	0	2	8	2	2	0.4	0.0025
ATP-binding cassette sub-family B member 7, mitochondrial OS=Mus musculus GN=Abcb7 PE=1 SV=3	Q61102	83 kDa	5	0	0	0	7	2	2	2	0.4	0.0068
Succinate dehydrogenase [ubiquinone] iron-sulfur subunit, mitochondrial OS=Mus musculus GN=Sdhb PE=1 SV=1	Q9CQA3	32 kDa	5	0	0	0	7	2	2	2	0.4	0.0068
Splicing factor 3B subunit 3 OS=Mus musculus GN=Sf3b3 PE=1 SV=1	Q921M3	136 kDa	96	103	53	44	137	134	61	27	0.3	< 0.00010
Actin, cytoplasmic 1 OS=Mus musculus GN=Actb PE=1 SV=1	P60710 (+1)	42 kDa	32	33	13	20	46	43	15	12	0.3	< 0.00010
Poly(rC)-binding protein 1 OS=Mus musculus GN=Pcbp1 PE=1 SV=1	P60335	37 kDa	24	21	8	11	34	27	9	7	0.3	< 0.00010
U2 small nuclear ribonucleoprotein A' OS=Mus musculus GN=Snrpa1 PE=1 SV=2	P57784	28 kDa	20	19	8	13	28	25	9	8	0.3	< 0.00010
p21-activated protein kinase-interacting protein 1 OS=Mus musculus GN=Pak1ip1 PE=1 SV=2	Q9DCE5	42 kDa	14	23	6	11	20	30	7	7	0.3	< 0.00010
Splicing factor U2AF 65 kDa subunit OS=Mus musculus GN=U2af2 PE=1 SV=3	P26369	54 kDa	16	16	6	10	23	21	7	6	0.3	< 0.00010
Glutathione S-transferase Mu 1 OS=Mus musculus GN=Gstm1 PE=1 SV=2	P10649	26 kDa	18	11	7	6	26	14	8	4	0.3	< 0.00010
Cleavage and polyadenylation specificity factor subunit 1 OS=Mus musculus GN=Cpsf1 PE=1 SV=1	Q9EPU4	161 kDa	9	14	6	5	13	18	7	3	0.3	0.00025
U4/U6.U5 tri-snRNP-associated protein 2 OS=Mus musculus GN=Usp39 PE=1 SV=2	Q3TIX9	65 kDa	8	8	2	6	11	10	2	4	0.3	0.0029
U1 small nuclear ribonucleoprotein 70 kDa OS=Mus musculus GN=Snrnp70 PE=1 SV=2	Q62376	52 kDa	6	8	4	3	9	10	5	2	0.3	0.0053
Protein PML OS=Mus musculus GN=Pml PE=1 SV=3	Q60953	98 kDa	8	5	3	4	11	6	3	2	0.3	0.0097
Calcium-binding mitochondrial carrier protein Aralar2 OS=Mus musculus GN=Slc25a13 PE=1 SV=1	Q9QXX4	74 kDa	8	4	0	6	11	5	2	4	0.3	0.0098
Developmental pluripotency-associated protein 4 OS=Mus musculus GN=Dppa4 PE=1 SV=2	Q8CCG4	33 kDa	9	7	0	7	13	9	2	4	0.3	0.0014
Crooked neck-like protein 1 OS=Mus musculus GN=Crnkl1 PE=1 SV=1	P63154	83 kDa	9	4	2	5	13	5	2	3	0.3	0.0097
RNA-binding protein 39 OS=Mus musculus GN=Rbm39 PE=1 SV=2	Q8VH51	59 kDa	5	8	0	6	7	10	2	4	0.3	0.0051
Peptidyl-prolyl cis-trans isomerase-like 1 OS=Mus musculus GN=Ppil1 PE=2 SV=1	Q9D0W5	18 kDa	7	4	0	1	10	5	2	2	0.3	0.00013
Casein kinase II subunit alpha OS=Mus musculus GN=Csnk2a1 PE=1 SV=2	Q60737	45 kDa	3	6	0	2	4	8	2	2	0.3	0.0031
Small nuclear ribonucleoprotein Sm D2 OS=Mus musculus GN=Snrpd2 PE=1 SV=1	P62317	14 kDa	6	7	0	4	9	9	2	2	0.3	0.001
Dimethyladenosine transferase 1, mitochondrial OS=Mus musculus GN=Tfb1m PE=1 SV=1	Q8JZM0	39 kDa	7	3	0	1	10	4	2	2	0.3	0.00033
Splicing factor 3B subunit 6 OS=Mus musculus GN=Sf3b6 PE=1 SV=1	P59708	15 kDa	6	5	0	0	9	6	2	2	0.3	< 0.00010
Hexokinase OS=Mus musculus GN=Hk2 PE=1 SV=1	E9Q5B5 (+1)	99 kDa	9	0	0	1	13	2	2	2	0.3	0.00083
Probable ATP-dependent RNA helicase DDX46 OS=Mus musculus GN=Ddx46 PE=1 SV=1	F8WHR6 (+1)	117 kDa	2	8	0	0	3	10	2	2	0.3	< 0.00010

Myc-associated zinc finger protein OS=Mus musculus GN=Maz PE=4 SV=1	F8VPK3 (+1)	49 kDa	4	7	0	0	6	9	2	2	0.3	< 0.00010
MCG55033 OS=Mus musculus GN=Gm10250 PE=4 SV=1	G3X9L6 (+1)	19 kDa	5	5	0	0	7	6	2	2	0.3	< 0.00010
40S ribosomal protein S30 OS=Mus musculus GN=Fau PE=1 SV=1	P62862	7 kDa	6	5	0	0	9	6	2	2	0.3	< 0.00010
SAP30-binding protein OS=Mus musculus GN=Sap30bp PE=1 SV=2	Q02614	34 kDa	4	7	0	0	6	9	2	2	0.3	< 0.00010
Splicing factor 3B subunit 5 OS=Mus musculus GN=Sf3b5 PE=1 SV=1	Q923D4	10 kDa	7	4	0	0	10	5	2	2	0.3	< 0.00010
Uncharacterized protein C19orf43 homolog OS=Mus musculus PE=1 SV=1	Q9D735	18 kDa	5	6	0	0	7	8	2	2	0.3	< 0.00010
Pre-mRNA-splicing factor 38B OS=Mus musculus GN=Prpf38b PE=1 SV=1	Q80SY5	64 kDa	5	4	0	0	7	5	2	2	0.3	0.00012
Cytochrome c oxidase subunit NDUFA4 OS=Mus musculus GN=Ndufa4 PE=1 SV=2	Q62425	9 kDa	3	6	0	0	4	8	2	2	0.3	0.00012
Heterogeneous nuclear ribonucleoprotein H2 OS=Mus musculus GN=Hnrnph2 PE=1 SV=1	P70333	49 kDa	0	10	0	0	2	13	2	2	0.3	< 0.00010
Pre-mRNA-processing-splicing factor 8 OS=Mus musculus GN=Prpf8 PE=1 SV=2	Q99PV0	274 kDa	132	135	25	56	188	175	29	34	0.2	< 0.00010
U5 small nuclear ribonucleoprotein 200 kDa helicase OS=Mus musculus GN=Snrnp200 PE=1 SV=1	Q6P4T2	245 kDa	89	106	25	49	127	138	29	30	0.2	< 0.00010
Pre-mRNA-splicing factor ATP-dependent RNA helicase DHX15 OS=Mus musculus GN=Dhx15 PE=1 SV=2	O35286	91 kDa	72	77	17	24	102	100	20	14	0.2	< 0.00010
Protein Sf3b2 OS=Mus musculus GN=Sf3b2 PE=1 SV=1	Q3UJB0	98 kDa	58	62	20	25	83	81	23	15	0.2	< 0.00010
Protein RCC2 OS=Mus musculus GN=Rcc2 PE=1 SV=1	Q8BK67	56 kDa	39	44	12	10	55	57	14	6	0.2	< 0.00010
Kinesin-like protein KIFC1 OS=Mus musculus GN=Kifc1 PE=1 SV=2	Q9QWT9	74 kDa	23	23	7	12	33	30	8	7	0.2	< 0.00010
RNA-binding protein 10 OS=Mus musculus GN=Rbm10 PE=1 SV=1	Q99KG3	103 kDa	16	24	7	7	23	31	8	4	0.2	< 0.00010
ADP/ATP translocase 2 OS=Mus musculus GN=Slc25a5 PE=1 SV=3	P51881	33 kDa	22	26	6	11	31	34	7	7	0.2	< 0.00010
Pre-mRNA-processing factor 6 OS=Mus musculus GN=Prpf6 PE=1 SV=1	Q91YR7	107 kDa	15	25	4	12	21	32	5	7	0.2	< 0.00010
Nuclear pore complex protein Nup155 OS=Mus musculus GN=Nup155 PE=1 SV=1	Q99P88	155 kDa	15	19	3	7	21	25	3	4	0.2	< 0.00010
Splicing factor 45 OS=Mus musculus GN=Rbm17 PE=1 SV=1	Q8JZX4	45 kDa	13	16	5	5	18	21	6	3	0.2	< 0.00010
SNW domain-containing protein 1 OS=Mus musculus GN=Snw1 PE=1 SV=1	A0A0B4J1E2 (+1)	61 kDa	10	19	5	5	14	25	6	3	0.2	< 0.00010
Ribosomal RNA small subunit methyltransferase NEP1 OS=Mus musculus GN=Emg1 PE=1 SV=1	O35130	27 kDa	7	18	2	9	10	23	2	5	0.2	< 0.00010
DNA-directed RNA polymerase II subunit RPB2 OS=Mus musculus GN=Polr2b PE=1 SV=2	Q8CFI7	134 kDa	8	14	3	5	11	18	3	3	0.2	< 0.00010
Small nuclear ribonucleoprotein-associated protein B OS=Mus musculus GN=Snrpb PE=1 SV=1	P27048	24 kDa	7	14	2	6	10	18	2	4	0.2	0.0001
MCG18410, isoform CRA_a OS=Mus musculus GN=Ddx23 PE=1 SV=1	D3Z0M9	95 kDa	8	12	3	3	11	16	3	2	0.2	< 0.00010
Pleiotropic regulator 1 OS=Mus musculus GN=Plrg1 PE=1 SV=1	D3Z4V1 (+1)	56 kDa	10	13	0	6	14	17	2	4	0.2	< 0.00010
40S ribosomal protein S20 OS=Mus musculus GN=Rps20 PE=1 SV=1	P60867	13 kDa	9	8	0	6	13	10	2	4	0.2	0.00033
U2 snRNP-associated SURP motif-containing protein OS=Mus musculus GN=U2surp PE=1 SV=3	Q6NV83	118 kDa	8	10	0	2	11	13	2	2	0.2	< 0.00010
Phosphate carrier protein, mitochondrial OS=Mus musculus GN=Slc25a3 PE=1 SV=1	Q8VEM8	40 kDa	11	6	0	6	16	8	2	4	0.2	0.00033
U4/U6 small nuclear ribonucleoprotein Prp4 OS=Mus musculus GN=Prpf4 PE=1 SV=1	Q9DAW6	58 kDa	10	10	0	5	14	13	2	3	0.2	< 0.00010
Pre-mRNA-splicing factor RBM22 OS=Mus musculus GN=Rbm22 PE=1 SV=1	Q8BHS3	47 kDa	7	8	0	2	10	10	2	2	0.2	< 0.00010
SURP and G-patch domain-containing protein 1 OS=Mus musculus GN=Sugp1 PE=1 SV=1	Q8CH02	73 kDa	7	10	0	0	10	13	2	2	0.2	< 0.00010
U1 small nuclear ribonucleoprotein A (Fragment) OS=Mus musculus GN=Snrpa PE=1 SV=1	D3Z0S6 (+1)	27 kDa	10	8	0	6	14	10	2	4	0.2	0.00016
Catenin delta-1 OS=Mus musculus GN=Ctnnd1 PE=1 SV=1	D3Z2H2 (+15)	99 kDa	9	9	0	0	13	12	2	2	0.2	< 0.00010
Programmed cell death protein 7 OS=Mus musculus GN=Pdcd7 PE=1 SV=1	Q3U2X6	54 kDa	10	7	0	0	14	9	2	2	0.2	< 0.00010
ATP-dependent RNA helicase DDX42 OS=Mus musculus GN=Ddx42 PE=1 SV=3	Q810A7	102 kDa	7	12	0	0	10	16	2	2	0.2	< 0.00010
PHD finger-like domain-containing protein 5A OS=Mus musculus GN=Phf5a PE=1 SV=1	P83870	12 kDa	4	9	0	3	6	12	2	2	0.2	0.00036
Poly(rC)-binding protein 2 OS=Mus musculus GN=Pcbp2 PE=1 SV=1	Q61990	38 kDa	13	10	0	7	18	13	2	4	0.2	< 0.00010
Pre-mRNA-splicing factor SYF1 OS=Mus musculus GN=Xab2 PE=1 SV=1	Q9DCD2	100 kDa	6	6	0	3	9	8	2	2	0.2	0.00081
PAX3- and PAX7-binding protein 1 OS=Mus musculus GN=Paxbp1 PE=1 SV=3	P58501	105 kDa	6	7	0	0	9	9	2	2	0.2	< 0.00010
Bifunctional lysine-specific demethylase and histidyl-hydroxylase MINA OS=Mus musculus GN=Mina PE=1 SV=2	Q8CD15	54 kDa	11	2	0	0	16	3	2	2	0.2	< 0.00010
Protein FRG1 OS=Mus musculus GN=Frg1 PE=1 SV=2	P97376	29 kDa	4	10	0	0	6	13	2	2	0.2	< 0.00010
UPF0428 protein CXorf56 homolog OS=Mus musculus PE=1 SV=1	Q8VDP2	26 kDa	4	9	0	0	6	12	2	2	0.2	< 0.00010
Voltage-dependent anion-selective channel protein 1 OS=Mus musculus GN=Vdac1 PE=1 SV=3	Q60932	32 kDa	6	7	0	0	9	9	2	2	0.2	< 0.00010
Transcriptional repressor CTCF OS=Mus musculus GN=Ctcf PE=1 SV=2	Q61164	84 kDa	0	11	0	0	2	14	2	2	0.2	< 0.00010
Splicing factor 3B subunit 1 OS=Mus musculus GN=Sf3b1 PE=1 SV=1	G5E866 (+1)	146 kDa	128	101	16	19	182	131	18	11	0.1	< 0.00010
Steroid hormone receptor ERR2 OS=Mus musculus GN=Esrrb PE=1 SV=1	E9QKA2 (+2)	49 kDa	17	21	3	2	24	27	3	2	0.1	< 0.00010
Splicing factor 1 OS=Mus musculus GN=Sf1 PE=1 SV=1	D3YZC9	62 kDa	17	23	3	0	24	30	3	2	0.1	< 0.00010
Intron-binding protein aquarius OS=Mus musculus GN=Aqr PE=1 SV=1	A2AQA7 (+1)	162 kDa	13	15	0	4	18	19	2	2	0.1	< 0.00010
60S ribosomal protein L38 OS=Mus musculus GN=Rpl38 PE=3 SV=3	Q9JJI8	8 kDa	14	15	0	0	20	19	2	2	0.1	< 0.00010
U2 small nuclear ribonucleoprotein B'' OS=Mus musculus GN=Snrpb2 PE=1 SV=1	Q9CQI7	25 kDa	15	13	0	4	21	17	2	2	0.1	< 0.00010
Glutathione S-transferase P 1 OS=Mus musculus GN=Gstp1 PE=1 SV=2	P19157	24 kDa	9	17	0	0	13	22	2	2	0.1	< 0.00010
Cell division cycle and apoptosis regulator protein 1 OS=Mus musculus GN=Ccar1 PE=1 SV=1	Q8CH18	132 kDa	12	17	0	0	17	22	2	2	0.1	< 0.00010
Proline/serine-rich coiled-coil protein 1 OS=Mus musculus GN=Psrc1 PE=1 SV=2	Q9D0P7	35 kDa	13	12	0	3	18	16	2	2	0.1	< 0.00010
Tuftelin-interacting protein 11 OS=Mus musculus GN=Tfip11 PE=1 SV=1	Q9ERA6	96 kDa	15	11	0	0	21	16	2	2	0.1	< 0.00010
RNA-binding protein 40 OS=Mus musculus GN=Rnpc3 PE=2 SV=2	Q3UZ01	58 kDa	11	12	0	0	16	16	2	2	0.1	< 0.00010
RNA-binding protein 25 OS=Mus musculus GN=Rbm25 PE=1 SV=2	B2RY56	100 kDa	11	11	0	0	16	14	2	2	0.1	< 0.00010
Cilia- and flagella-associated protein 20 OS=Mus musculus GN=Cfap20 PE=1 SV=1	Q8BTU1	23 kDa	11	12	0	0	16	16	2	2	0.1	< 0.00010
Splicing factor U2AF 35 kDa subunit OS=Mus musculus GN=U2af1 PE=1 SV=4	Q9D883	28 kDa	12	12	0	0	17	16	2	2	0.1	< 0.00010
Cysteine and glycine-rich protein 2 OS=Mus musculus GN=Csrp2 PE=1 SV=3	P97314	21 kDa	11	11	0	0	16	14	2	2	0.1	< 0.00010
Splicing factor 3A subunit 1 OS=Mus musculus GN=Sf3a1 PE=1 SV=1	Q8K4Z5	89 kDa	52	52	6	9	74	68	7	5	0.09	< 0.00010
Small nuclear ribonucleoprotein Sm D1 OS=Mus musculus GN=Snrpd1 PE=1 SV=1	P62315	13 kDa	17	15	0	0	24	19	2	2	0.09	< 0.00010
U5 small nuclear ribonucleoprotein 40 kDa protein OS=Mus musculus GN=Snrnp40 PE=1 SV=1	Q6PE01	39 kDa	22	22	0	5	31	29	2	3	0.08	< 0.00010
Splicing factor 3A subunit 2 OS=Mus musculus GN=Sf3a2 PE=1 SV=1	G3UVU2	51 kDa	17	18	0	0	24	23	2	2	0.08	< 0.00010
RNA-binding protein 5 OS=Mus musculus GN=Rbm5 PE=1 SV=1	Q91YE7	92 kDa	13	23	0	0	18	30	2	2	0.08	< 0.00010
ADP/ATP translocase 1 OS=Mus musculus GN=Slc25a4 PE=1 SV=4	P48962	33 kDa	16	24	0	4	23	31	2	2	0.08	< 0.00010
Protein Dhx35 OS=Mus musculus GN=Dhx35 PE=1 SV=1	A2ACQ1	79 kDa	20	24	0	3	28	31	2	2	0.07	< 0.00010
Probable ATP-dependent RNA helicase DDX41 OS=Mus musculus GN=Ddx41 PE=1 SV=2	Q91VN6	70 kDa	36	25	0	3	51	32	2	2	0.05	< 0.00010
Calcium homeostasis endoplasmic reticulum protein OS=Mus musculus GN=Cherp PE=1 SV=1	G5E8I8 (+1)	106 kDa	27	27	0	2	38	35	2	2	0.05	< 0.00010
Splicing factor 3A subunit 3 OS=Mus musculus GN=Sf3a3 PE=2 SV=2	Q9D554	59 kDa	28	29	0	0	40	38	2	2	0.05	< 0.00010

Table S2.

Primer sequences

Name	Sequences	Application	Reference or PrimerBank ID
Zfp296-F1	TCCAGTGTGGCAGACAGTAC	Genotyping	
Zfp296-R1	GGGCAGCACTGCTCACTGG	Genotyping, RT-PCR	
Zfp296-F2 (Neo-F)	CCTTCTATCGCCTTCTTGACG	Genotyping	
Zfp296-F3	CGCGTAGATCCCGATACCG	Genotyping, RT-PCR	12843135a1
Zfp296-RT-F	TTAGGGGCCATCATCGCTTTC	qRT-PCR	294997323c3
Zfp296-RT-R	GCCTCTGGGGTATCTAGGTGT	qRT-PCR	294997323c3
Tbp-F	GAAGAACAATCCAGACTAGCAGCA	qRT-PCR	Kelee et al., 2011
Tbp-R	CCTTATAGGGAACTTCACATCACAG	qRT-PCR	Kelee et al., 2011
Suv39h1-F	TGTCAACCATAGTTGTGATCC	qRT-PCR	Alder et al., 2010
Suv39h1-R	ATTCGGGTACTCTCCATGTC	qRT-PCR	Alder et al., 2010
Suv39h2-F	ATCTACGAATGCAACTCAAGGTG	qRT-PCR	Alder et al., 2010
Suv39h2-R	CCACAGCCATTGCTAGTTCTAA	qRT-PCR	Alder et al., 2010
GLP-F	CAGATGGAGAAACAAATGGGTCT	qRT-PCR	Alder et al., 2010
GLP-R	TTTGCTTCCCCACTTCTGTGT	qRT-PCR	Alder et al., 2010
G9a-F	TCACCCTGACTGACAATGAG	qRT-PCR	Alder et al., 2010
G9a-R	AGACAGGAACAACAGAACAC	qRT-PCR	Alder et al., 2010
Eset-F	GAGGCCGGGGAGAGGCTGAA	qRT-PCR	Alder et al., 2010t
Eset-R	TAGCTGGTCGGCGAAGCCCT	qRT-PCR	Alder et al., 2010
Gapdh-F	TGTGTCCGTCGTGGATCTGA	qRT-PCR	Suzuki et al., 2003
Gapdh-R	CCTGCTTCACCACCTTCTTGA	qRT-PCR	Suzuki et al., 2003
Actb-F	AGTGTGACGTTGACATCCGTA	qRT-PCR	Huntington et al., 2007
Actb-R	GCCAGAGCAGTAATCTCCTTCT	qRT-PCR	Huntington et al., 2007
Jmjd1a-F	CACATTTAGGTTCCCAGTCACA	qRT-PCR	Zhao et al., 2013
Jmjd1a-R	GCCACGATGTTAACACAGGA	qRT-PCR	Zhao et al., 2013
Jmjd2a-F	GACCACACTCTGCCCACAC	qRT-PCR	Zhao et al., 2013
Jmjd2a-R	TCCTGGGGTATTTCCAGACA	qRT-PCR	Zhao et al., 2013
Jmjd2b-F	GGCTTTAACTGCGCTGAGTC	qRT-PCR	Zhao et al., 2013
Jmjd2b-R	GTGTGGTCCAGCACTGTGAG	qRT-PCR	Zhao et al., 2013
Jmjd2c-F	CACGGAGGACATGGATCTCT	qRT-PCR	Zhao et al., 2013
Jmjd2c-R	CGAAGGGAATGCCATACTTC	qRT-PCR	Zhao et al., 2013
Major Sat-F	TGGAATATGGCGAGAAAACTG	ChIP qPCR	Bulut-Karslioglu et al., 2012
Major Sat-R	AGGTCCTTCAGTGGGCATTT	ChIP qPCR	Bulut-Karslioglu et al., 2012
L1_promoter-F	ACTGCGGTACATAGGGAAGC	ChIP qPCR	Bulut-Karslioglu et al., 2014
L1_promoter-R	TGTGATCCACTCACCAGAGG	ChIP qPCR	Bulut-Karslioglu et al., 2014
L1_ORF1-F	CACTCCCACCCCACCTAGT	ChIP qPCR	Bulut-Karslioglu et al., 2014
L1_ORF1-R	TAACTCTTTAGCAGTGCTCTCCTGT	ChIP qPCR	Bulut-Karslioglu et al., 2014
L1_ORF2-F	ACCTGGACGAAATGGACAAA	ChIP qPCR	Bulut-Karslioglu et al., 2014
L1_ORF2-R	CATCTGGTCCTGGGCTTTT	ChIP qPCR	Bulut-Karslioglu et al., 2014
L1_3’UTR-F	CCAGCAAACACAGAAGTGGA	ChIP qPCR	Bulut-Karslioglu et al., 2014
L1_3’UTR-R	CCGACTAGGCCATCTTTTGA	ChIP qPCR	Bulut-Karslioglu et al., 2014
IAP-F	GCACCCTCAAAGCCTATCTTA	ChIP qPCR	Bulut-Karslioglu et al., 2014
IAP-R	TCCCTTGGTCAGTCTGGATTT	ChIP qPCR	Bulut-Karslioglu et al., 2014
IAPEY3-F	ACAGAGGAGGACAACTGCTC	ChIP qPCR	Bulut-Karslioglu et al., 2014
IAPEY3-R	AACCTTACACAGGCAAAAGC	ChIP qPCR	Bulut-Karslioglu et al., 2014
ETnERV2-F	ACAAATTCAGTATGGGCATC	ChIP qPCR	Bulut-Karslioglu et al., 2014
ETnERV2-R	GGGTACTGTTAAGACCCACA	ChIP qPCR	Bulut-Karslioglu et al., 2014
MMETn_mid-F	GGCTGGGAGGATGAGAAAA	ChIP qPCR	Bulut-Karslioglu et al., 2014
MMETn_mid-R	ATCCTTGCTCTCACCCAAAA	ChIP qPCR	Bulut-Karslioglu et al., 2014
Brd2-F	CCTACGCTGTCTTGGGACTC	ChIP qPCR	Bulut-Karslioglu et al., 2014
Brd2-R	AGGCCAGTTGGGGCTATAAT	ChIP qPCR	Bulut-Karslioglu et al., 2014
Tpsg1-F	CCCTGTGTTGTCACATCCTG	ChIP qPCR	Bulut-Karslioglu et al., 2014
Tpsg1-R	CTGTGTCCCTGGGTCTCCTA	ChIP qPCR	Bulut-Karslioglu et al., 2014

Table S3.

Antibodies

Name		Dilution	Application
Anti-Zfp296	raised in this study	1: 100	IF
Anti-Zfp296	raised in this study	1: 5000	WB
Anti-H3	Abcam; ab1791	1: 40000	WB
Anti-H3K9me1	Active Motif; 39681	1:200	IF
Anti-H3K9me1	Active Motif; 39681	1: 5000	WB
Anti-H3K9me2	Upstate; #07-441	1: 300	IF
Anti-H3K9me2	Abcam; ab1220	1: 5000	WB
Anti-H3K9me3	Abcam; ab8898	1: 200	IF
Anti-H3K9me3	Abcam; ab8898	1: 5000	WB
Anti-H3K9me3	Abcam; ab8898	1: 500	ChIP
Anti-H3K27me3	Upstate; #07-449	1: 300	IF
Anti-H3K27me3	Upstate; #07-449	1: 5000	WB
Anti-TRA98	Tanaka et al., 1997	1: 1000	IF
Anti-PGC7	R&D Systems; AF2566	1: 100	IF
Anti-Oct3/4	Santa Cruz; sc-5279 (clone C-10)	1: 100	IF
Anti-Nanog	ReproCell; RCAB0001P	1: 100	IF
Anti-MVH	Toyooka et al., 2000	1: 1000	IF
Anti-Kit	Chemicon; CBL1360	1: 50	IF
Anti-E-cadherin	R&D Systems; AF748	1: 100	IF
Anti-phospho Histone H3 (Ser10)	Upstate; #05-806	1: 500	IF
Anti-cleaved Caspase-3 (Asp175)	Cell Signaling; #9661	1: 200	IF
Anti-Flag tag	Sigma; F3165 (clone M2)	1: 1000	IF
Anti-Myc tag	MBL; M047-3 (clone PL14)	1: 10000	WB
Anti-GFP	Nacalai; 04404-26 (clone GF090R)	1: 5000	WB
Anti-β-tubulin	Sigma; T4026 (clone TUB 2.1)	1: 10000	WB
Anti-CD4-FITC	BD pharmingen; 553046 (clone RM4-5)	1: 100	FCM
Anti-CD8a-PE	BD pharmingen; 553032 (clone 53-6.7)	1: 100	FCM
Anti-TCRβ-FITC	BD pharmingen; 553170 (clone H57-597)	1: 100	FCM
Anti-TCRγδ-PE	BD pharmingen; 553178 (clone GL3)	1: 100	FCM
Anti-goat IgG-Alexa Fluor 488	Invitrogen; A-11055	1: 200	IF
Anti-mouse IgG_1-Alexa Fluor 488, 647	Invitrogen; A-21121, A-21240	1: 200	IF
Anti-mouse IgG_{2a}-Alexa Fluor 488	Invitrogen; A-21131	1: 200	IF
Anti-mouse IgG_{2b}-Alexa Fluor 488, 568	Invitrogen; A-21141, A-21144	1: 200	IF
Anti-mouse IgG-Alexa Fluor 647	Invitrogen; A-31571	1: 200	IF
Anti-rabbit IgG-Alexa Fluor 488, 568	Invitrogen; A-11008, A-11011	1: 200	IF
Anti-rabbit IgG-Alexa Fluor 594	Invitrogen; A-21207	1: 200	IF
Anti-rat IgG-Alexa Fluor 647	Invitrogen; A-21247	1: 200	IF
Anti-mouse IgG-HRP	MBL; 330	1: 10000	WB
Anti-rabbit IgG-HRP	Cell Signaling; #7071	1: 10000	WB
